# Supplementary material for: Organocatalytic Ring-Opening Polymerization of ε-Caprolactone Using bis(N-(N′-butylimidazolium)alkane Dicationic Ionic Liquids as the Metal-Free Catalysts: Polymer Synthesis, Kinetics and DFT Mechanistic Study
Source: Polymers (Basel). 2021 Dec 8;13(24):4290. doi: 10.3390/polym13244290 (PMC8705680; doi:10.3390/polym13244290)
Supplement: Supplementary file 1 [file polymers-13-04290-s001.zip › polymers-1468962-supplementary.pdf]

Organocatalytic ring-opening polymerization of *ε*-caprolactone using *bis*(*N*-(*N*'-butylimidazolium)alkane dicationic ionic liquids as the metal-free catalysts: Polymer synthesis, kinetics and DFT mechanistic study

Nathaporn Cheechana [1,2], Wachara Benchaphanthawee [1,2], Natthapol Akkravijitkul [1,2], Puracheth Rithchumpon [1,2], Thiti Junpirom [1], Wanich Limwanich [5], Winita Punyodom [1,3,4], Nawee Kungwan [1,3,4], Chanisorn Ngaojampa [1], Praput Thavornyutikarn [1] and Puttinan Meepowpan [1,3,4,*]

[1]Department of Chemistry, Faculty of Science, Chiang Mai University, 239 Huay Kaew Road, Chiang Mai 50200, Thailand
[2]Graduate School, Chiang Mai University, 239 Huay Kaew Road, Chiang Mai 50200, Thailand
[3]Center of Excellence for Innovation in Chemistry (PERCH-CIC), Faculty of Science, Chiang Mai University, 239 Huay Kaew Road, Chiang Mai 50200, Thailand
[4]Center of Excellence in Materials Science and Technology, Chiang Mai University, 239 Huay Kaew Road, Chiang Mai 50200, Thailand
[5]Faculty of Sciences and Agricultural Technology, Rajamangala University of Technology Lanna, 128 Huay Kaew Road, Chiang Mai, 50300, Thailand

Phone +66 5394 3341 5, Fax +66 5389 2277, [*]E-Mail: pmeepowpan@gmail.com

Table of contents

1. FT-IR, ^{1}H-NMR and ^{13}C-NMR Spectra

Fig. S1. FT-IR spectrum of 1,4-*bis*[Bim] (**6a**)……4
Fig. S2. FT-IR spectrum of 1,6-*bis*[Bim] (**6b**)……4
Fig. S3. FT-IR spectrum of 1,8-*bis*[Bim] (**6c**)……5
Fig. S4. FT-IR spectrum of 1,10-*bis*[Bim] (**6d**)……5
Fig. S5. FT-IR spectrum of 1,4-*bis*[Bim][Br] (**7a**)……6
Fig. S6. FT-IR spectrum of 1,6-*bis*[Bim][Br] (**7b**)……6
Fig. S7. FT-IR spectrum of 1,8-*bis*[Bim][Br] (**7c**)……7
Fig. S8. FT-IR spectrum of 1,10-*bis*[Bim][Br] (**7d**)……7
Fig. S9. FT-IR spectrum of 1,4-*bis*[Bim][PF_6] (**2a**)……8
Fig. S10. FT-IR spectrum of 1,6-*bis*[Bim][PF_6] (**2b**)……8
Fig. S11. FT-IR spectrum of 1,8-*bis*[Bim][PF_6] (**2c**)……9
Fig. S12. FT-IR spectrum of 1,10-*bis*[Bim][PF_6] (**2d**)……9
Fig. S13. ^{1}H-NMR spectrum of 1,4-*bis*[Bim] (**6a**) in MeOH-d_4……10
Fig. S14. ^{1}H-NMR spectrum of 1,6-*bis*[Bim] (**6b**) in $CDCl_3$……10
Fig. S15. ^{1}H-NMR spectrum of 1,8-*bis*[Bim] (**6c**) in $CDCl_3$……11
Fig. S16. ^{1}H-NMR spectrum of 1,10-*bis*[Bim] (**6d**) in $CDCl_3$……11
Fig. S17. ^{1}H-NMR spectrum of 1,4-*bis*[Bim][Br] (**7a**) in MeOH-d_4……12
Fig. S18. ^{1}H-NMR spectrum of 1,6-*bis*[Bim][Br] (**7b**) in MeOH-d_4……12

Fig. S19. ^{1}H-NMR spectrum of 1,8-*bis*[Bim][Br] (**7c**) in MeOH-d_4……………………….13

Fig. S20. ^{1}H-NMR spectrum of 1,10-*bis*[Bim][Br] (**7d**) in MeOH-d_4………………………13

Fig. S21. ^{1}H-NMR spectrum of 1,4-*bis*[Bim][PF_6] (**2a**) in MeOH-d_4………………………14

Fig. S22. ^{1}H-NMR spectrum of 1,6-*bis*[Bim][PF_6] (**2b**) in MeOH-d_4………………………14

Fig. S23. ^{1}H-NMR spectrum of 1,8-*bis*[Bim][PF_6] (**2c**) in MeOH-d_4……………………........15

Fig. S24. ^{1}H-NMR spectrum of 1,10-*bis*[Bim][PF_6] (**2d**) in MeOH-d_4……………………..15

Fig. S25. ^{13}C-NMR spectrum of 1,4-*bis*[Bim] (**6a**) in MeOH-d_4…………………………...16

Fig. S26. ^{13}C-NMR spectrum of 1,6-*bis*[Bim] (**6b**) in MeOH-d_4…………………………...16

Fig. S27. ^{13}C-NMR spectrum of 1,8-*bis*[Bim] (**6c**) in MeOH-d_4……………………………17

Fig. S28. ^{13}C-NMR spectrum of 1,10-*bis*[Bim] (**6d**) in MeOH-d_4…………………………17

Fig. S29. ^{13}C-NMR spectrum of 1,4-*bis*[Bim][Br] (**7a**) in MeOH-d_4…………………........18

Fig. S30. ^{13}C-NMR spectrum of 1,6-*bis*[Bim][Br] (**7b**) in MeOH-d_4…………………........18

Fig. S31. ^{13}C-NMR spectrum of 1,8-*bis*[Bim][Br] (**7c**) in MeOH-d_4…………………........19

Fig. S32. ^{13}C-NMR spectrum of 1,10-*bis*[Bim][Br] (**7d**) in MeOH-d_4……………………...19

Fig. S33. ^{13}C-NMR spectrum of 1,4-*bis*[Bim][PF_6] (**2a**) in MeOH-d_4……………………...20

Fig. S34. ^{13}C-NMR spectrum of 1,6-*bis*[Bim][PF_6] (**2b**) in MeOH-d_4……………………...20

Fig. S35. ^{13}C-NMR spectrum of 1,8-*bis*[Bim][PF_6] (**2c**) in MeOH-d_4……………………...21

Fig. S36. ^{13}C-NMR spectrum of 1,10-*bis*[Bim][PF_6] (**2d**) in MeOH-d_4……………….........21

2. Kinetic studies of the ROP of *ε*-caprolactone catalyzed by the synthesized DILs catalysts with *n*-dodecanol initiator

Fig. S37. ^{1}H-NMR spectrum of kinetic studies the ROP of CL……………………………..22
using 1,4-*bis*[Bim][PF_6] (**2a**) as catalyst with 1.0 mol% of 1-dodecanol at 150 °C

Fig. S38. ^{1}H-NMR spectrum of kinetic studies the ROP of CL……………………………..23
using 1,6-*bis*[Bim][PF_6] (**2b**) as catalyst with 1.0 mol% of 1-dodecanol at 150 °C

Fig. S39. ^{1}H-NMR spectrum of kinetic studies the ROP of CL……………………………..24
using 1,8-*bis*[Bim][PF_6] (**2c**) as catalyst with 1.0 mol% of 1-dodecanol at 150 °C

Fig. S40. ^{1}H-NMR spectrum of kinetic studies the ROP of CL……………………………..25
using 1,10-*bis*[Bim][PF_6] (**2a**) as catalyst with 1.0 mol% of 1-dodecanol at 150 °C

3. Differential scanning calorimetry thermogram of synthesized poly(*ε*-caprolactone)

Fig. S41. DSC curves of PCL (entry 25) obtained from the ROP of CL catalyzed by…..........26
1,4-*bis*[Bim][PF_6] (**2a**) with 1.0 mol% of 1-dodecanol

4. **Table S1.** Percentage of cell viability obtained from the cytotoxicity testing of the synthesized DILs catalysts (**2a**–**2d**) and DMSO at a concentration of 100 µg/mL. ……………………..…..26

5. Density Function Theory Calculations…………………………………………………………..27

1. FT-IR, ^{1}H-NMR and ^{13}C-NMR spectra

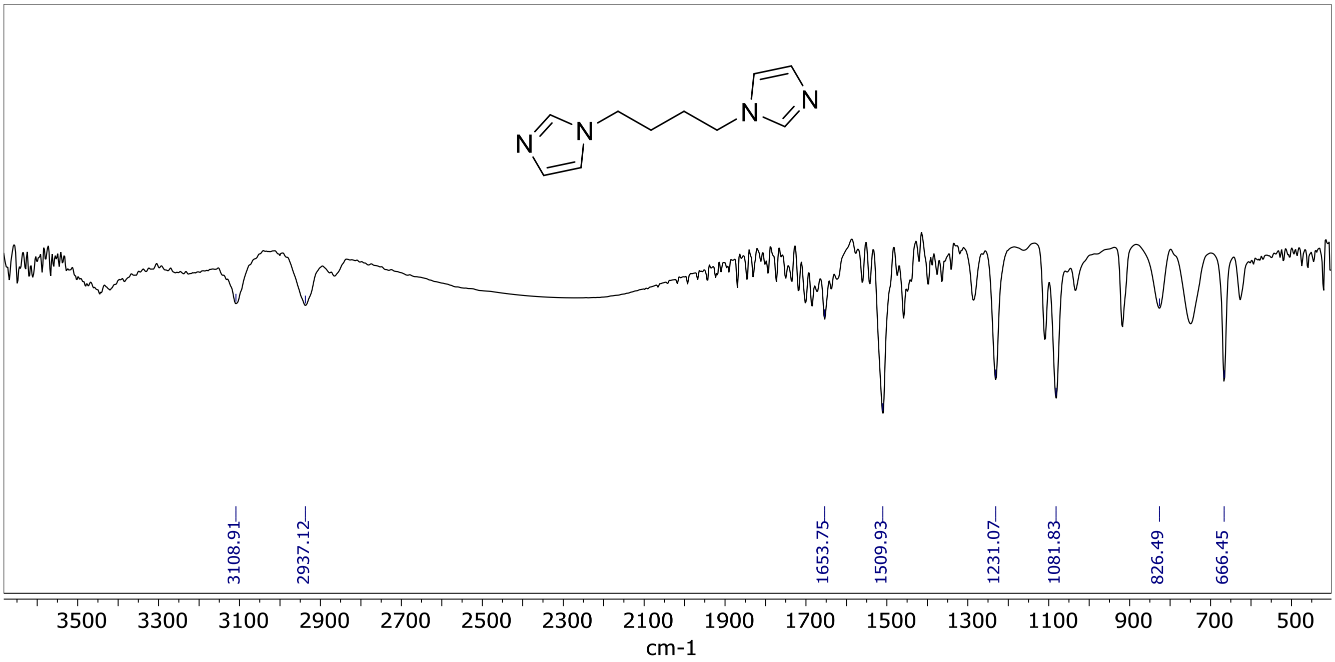

Fig. S1. FT-IR spectrum of 1,4-*bis*[Bim] (**6a**)

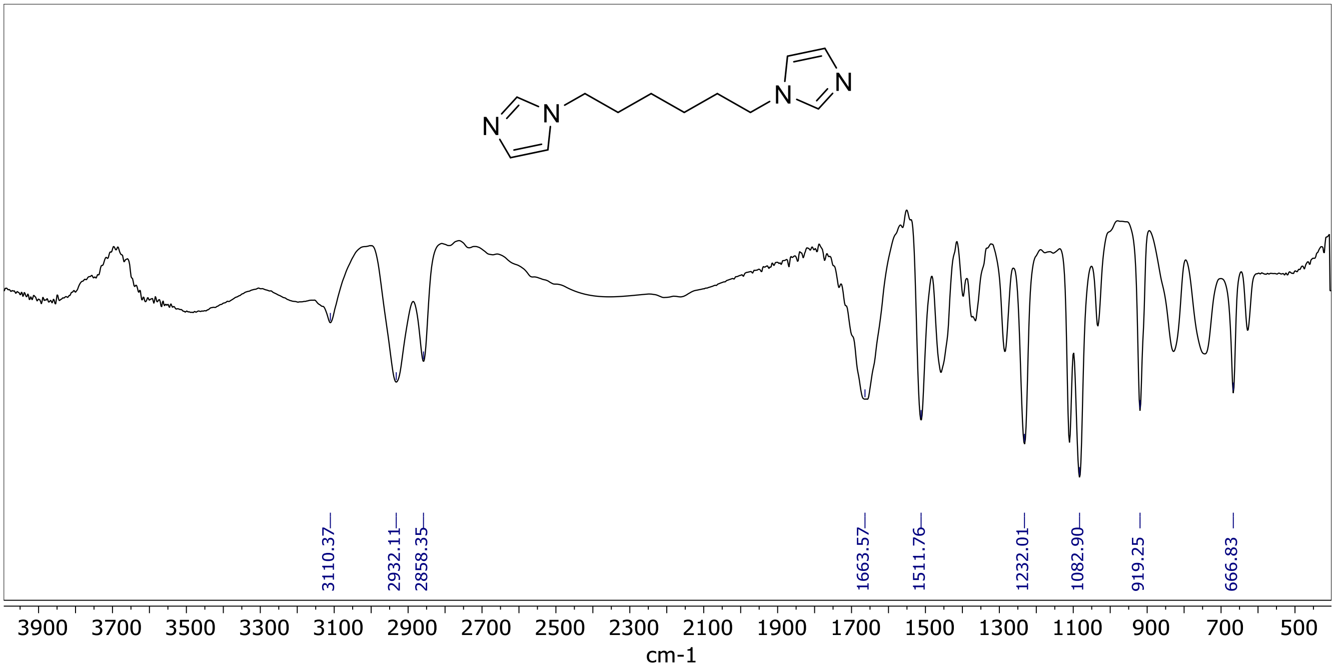

Fig. S2. FT-IR spectrum of 1,6-*bis*[Bim] (**6b**)

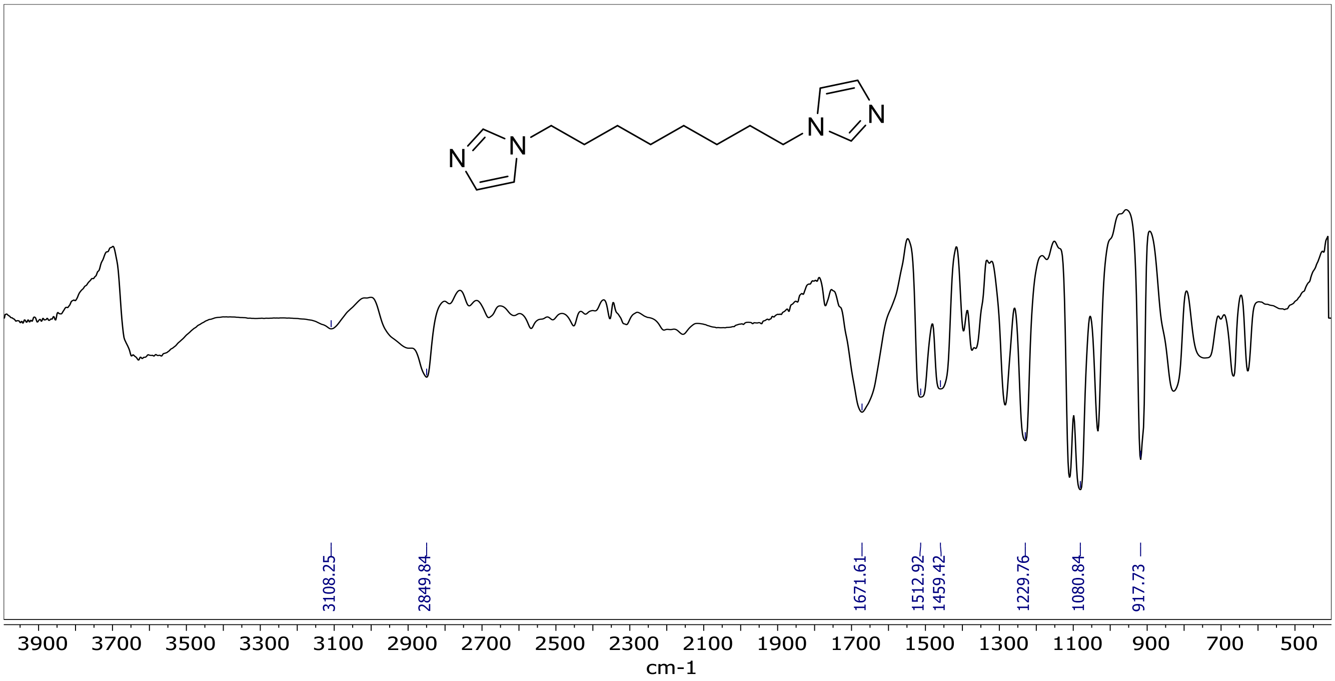

Fig. S3. FT-IR spectrum of 1,8-*bis*[Bim] (**6c**)

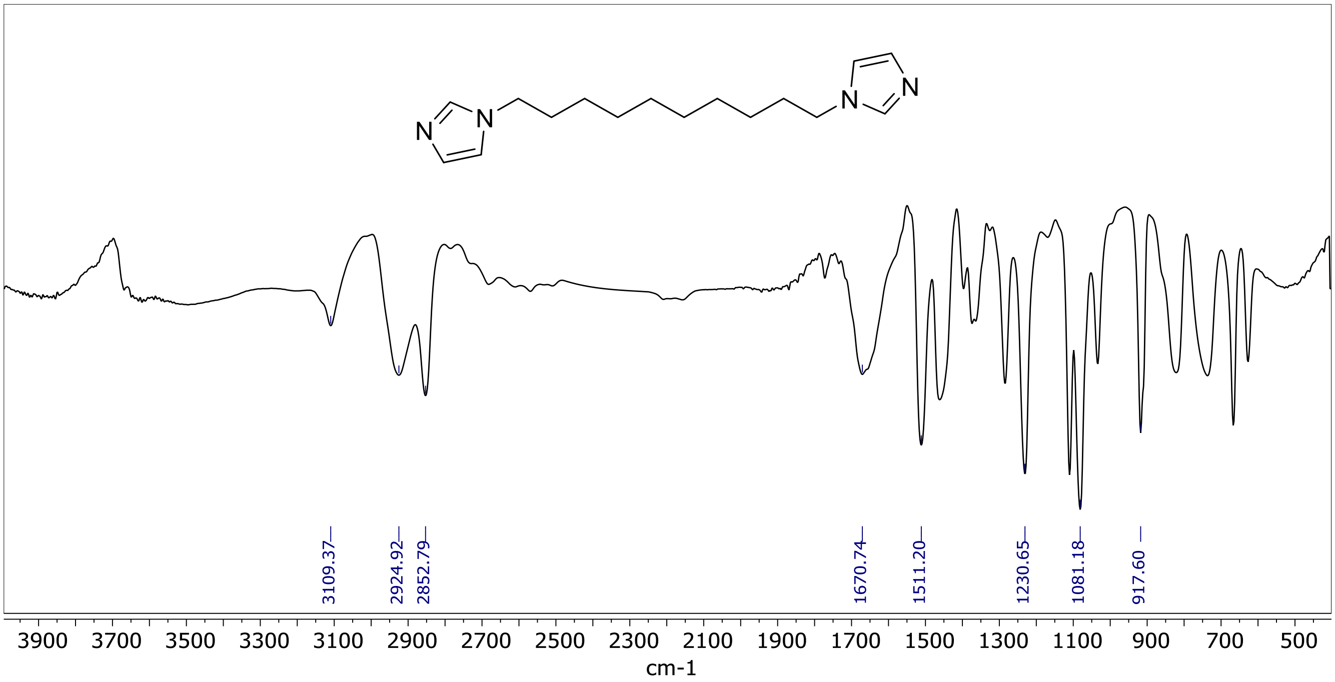

Fig. S4. FT-IR spectrum of 1,10-*bis*[Bim] (**6d**)

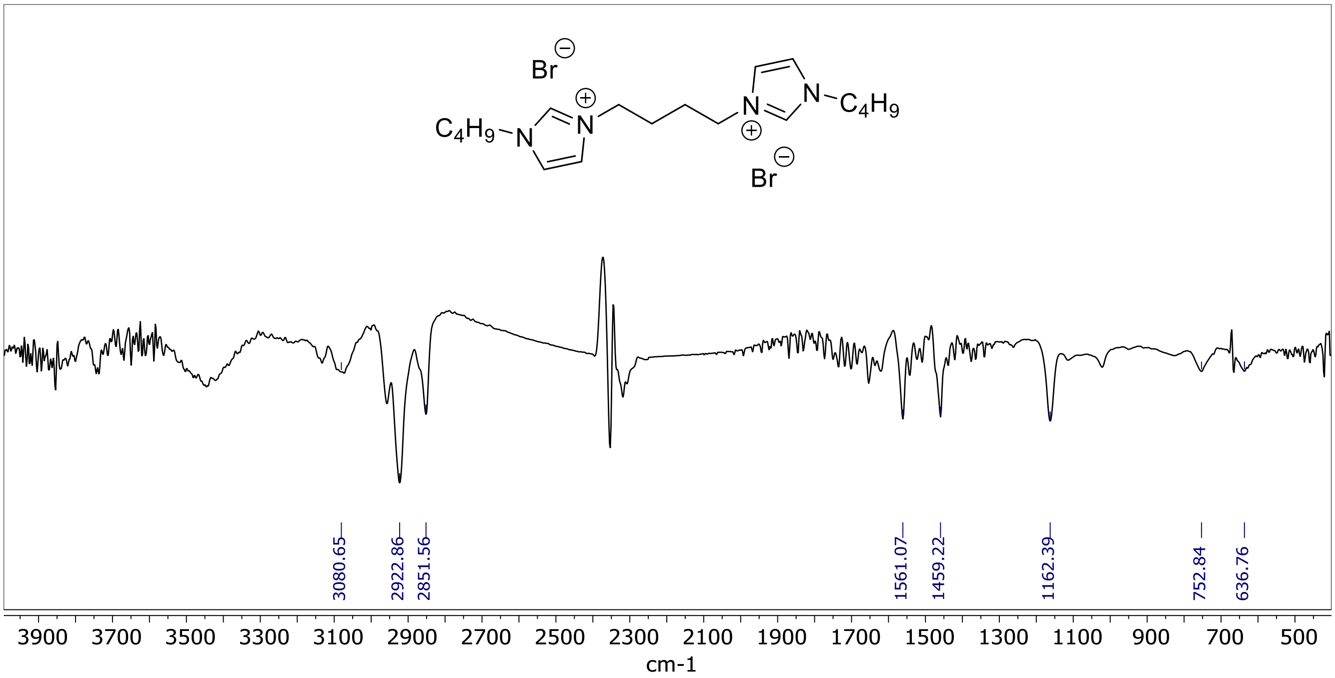

Fig. S5. FT-IR spectrum of 1,4-*bis*[Bim][Br] (**7a**)

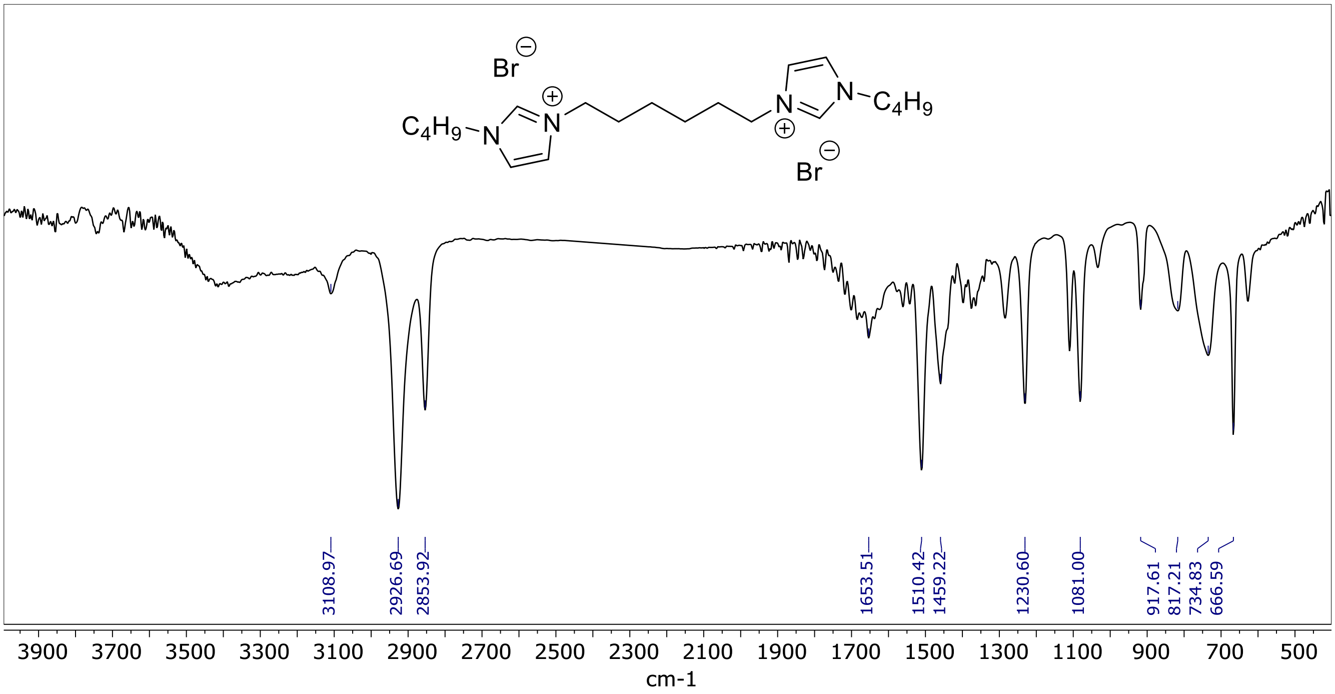

Fig. S6. FT-IR spectrum of 1,6-*bis*[Bim][Br] (**7b**)

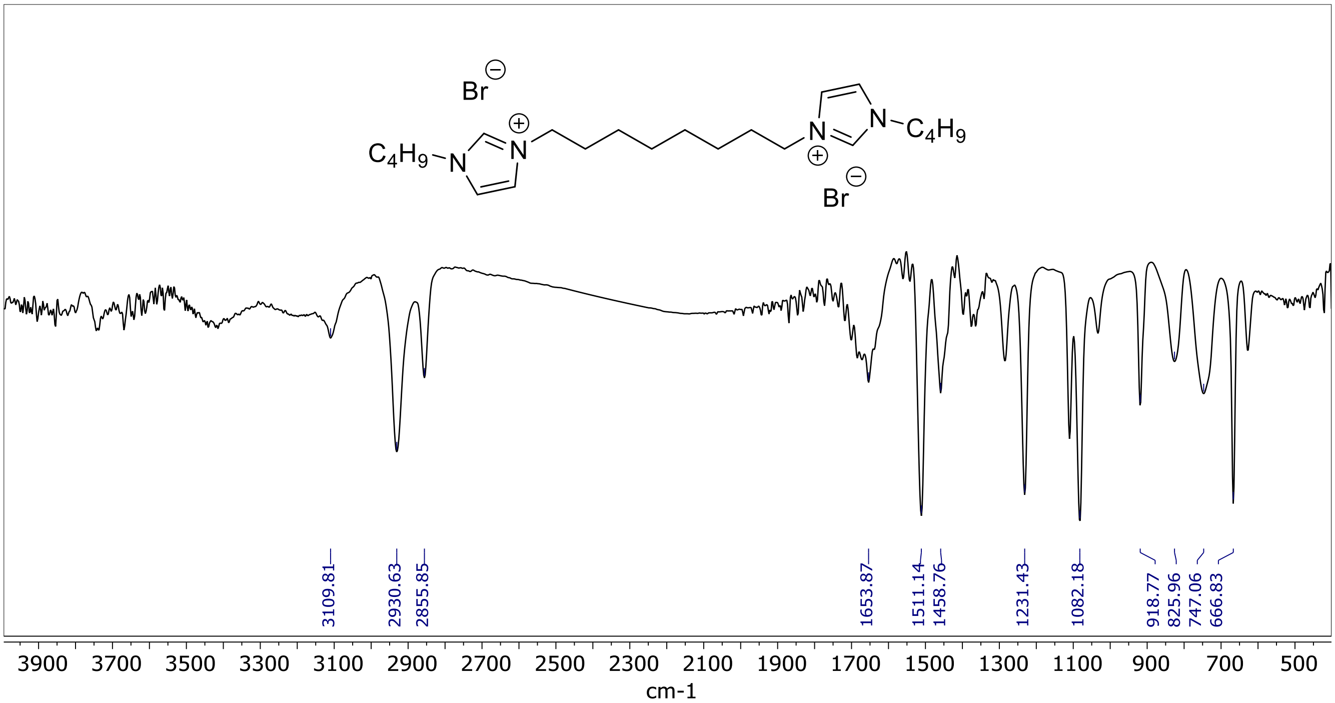

Fig. S7. FT-IR spectrum of 1,8-*bis*[Bim][Br] (**7c**)

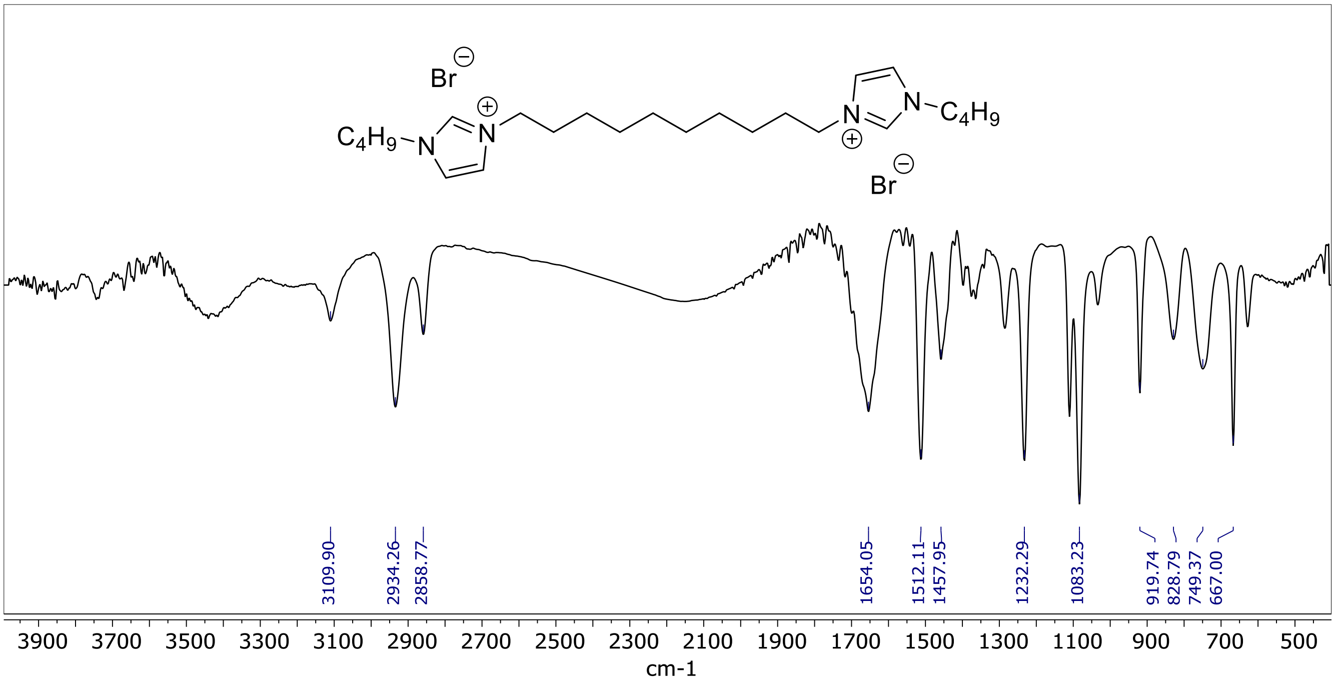

Fig. S8. FT-IR spectrum of 1,10-*bis*[Bim][Br] (**7d**)

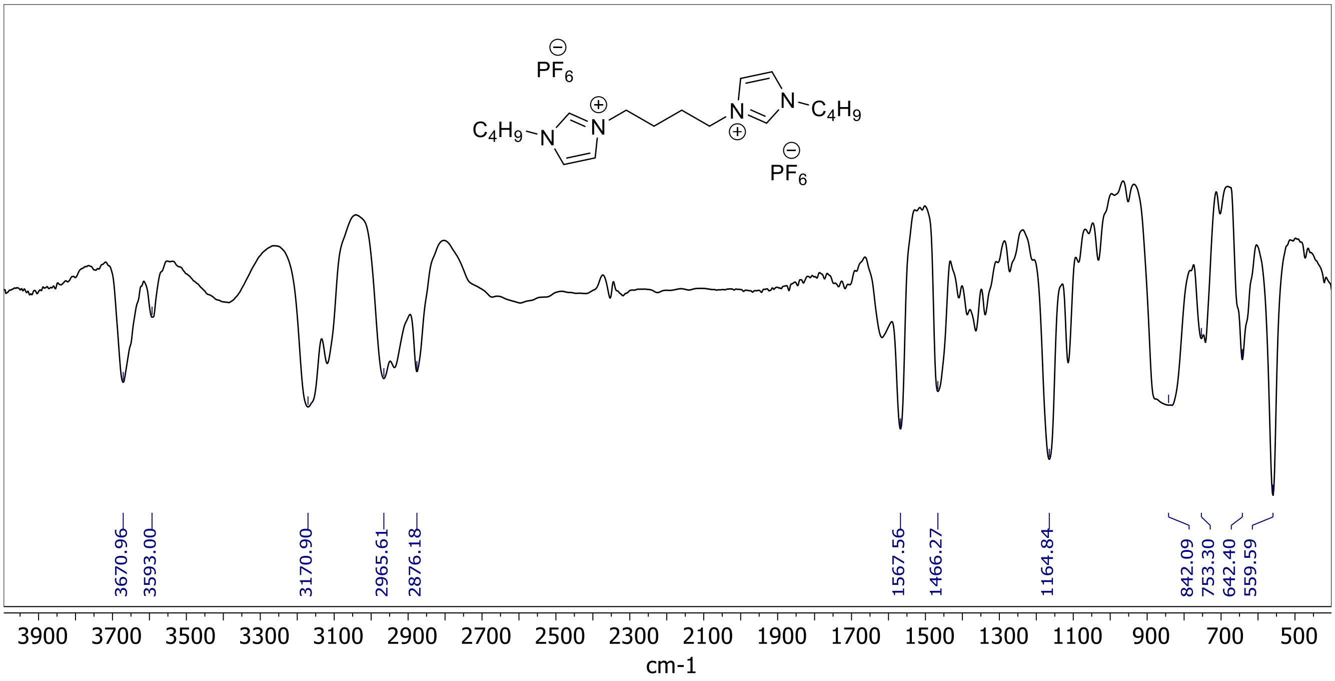

Fig. S9. FT-IR spectrum of 1,4-*bis*[Bim][PF_6] (**2a**)

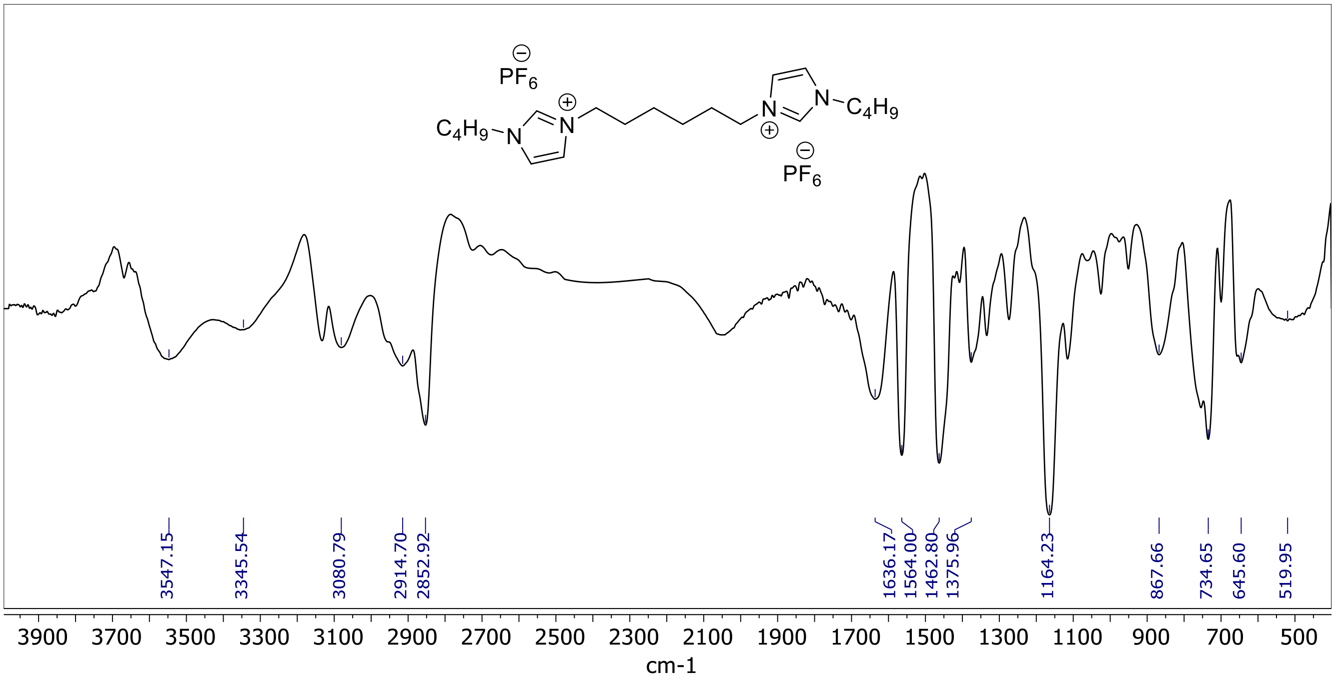

Fig. S10. FT-IR spectrum of 1,6-*bis*[Bim][PF_6] (**2b**)

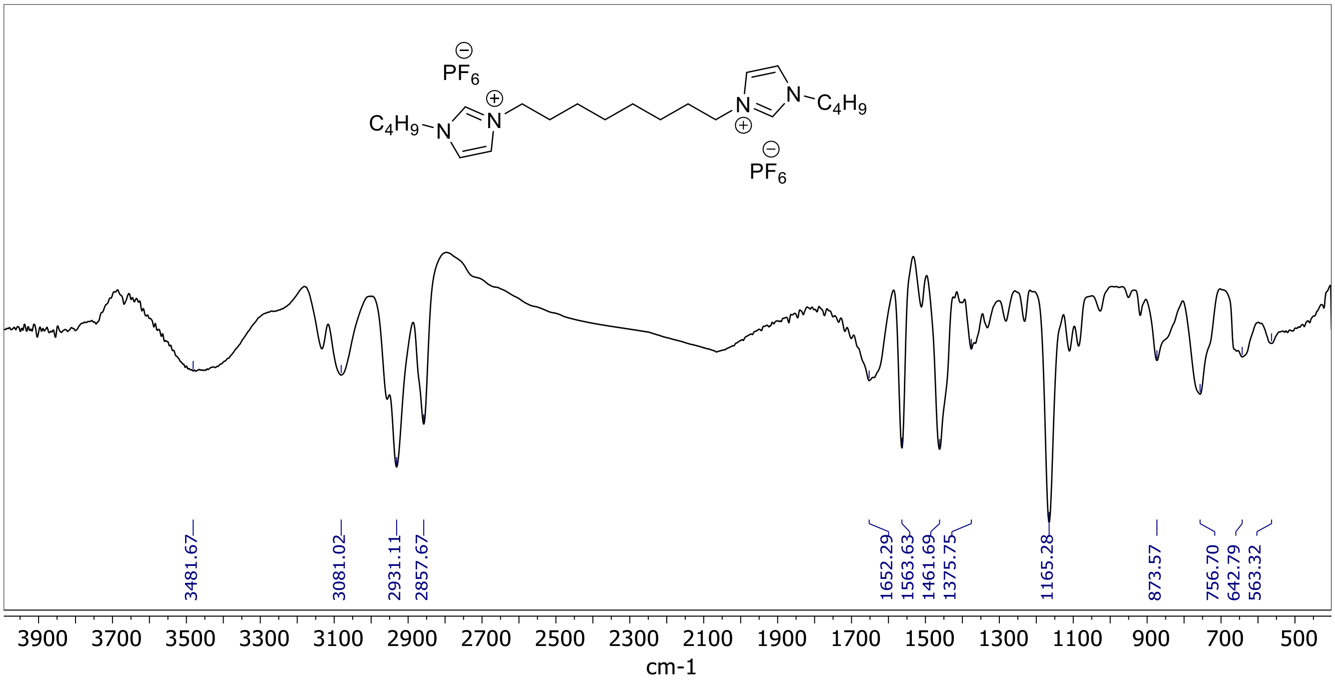

Fig. S11. FT-IR spectrum of 1,8-*bis*[Bim][PF_6] (**2c**)

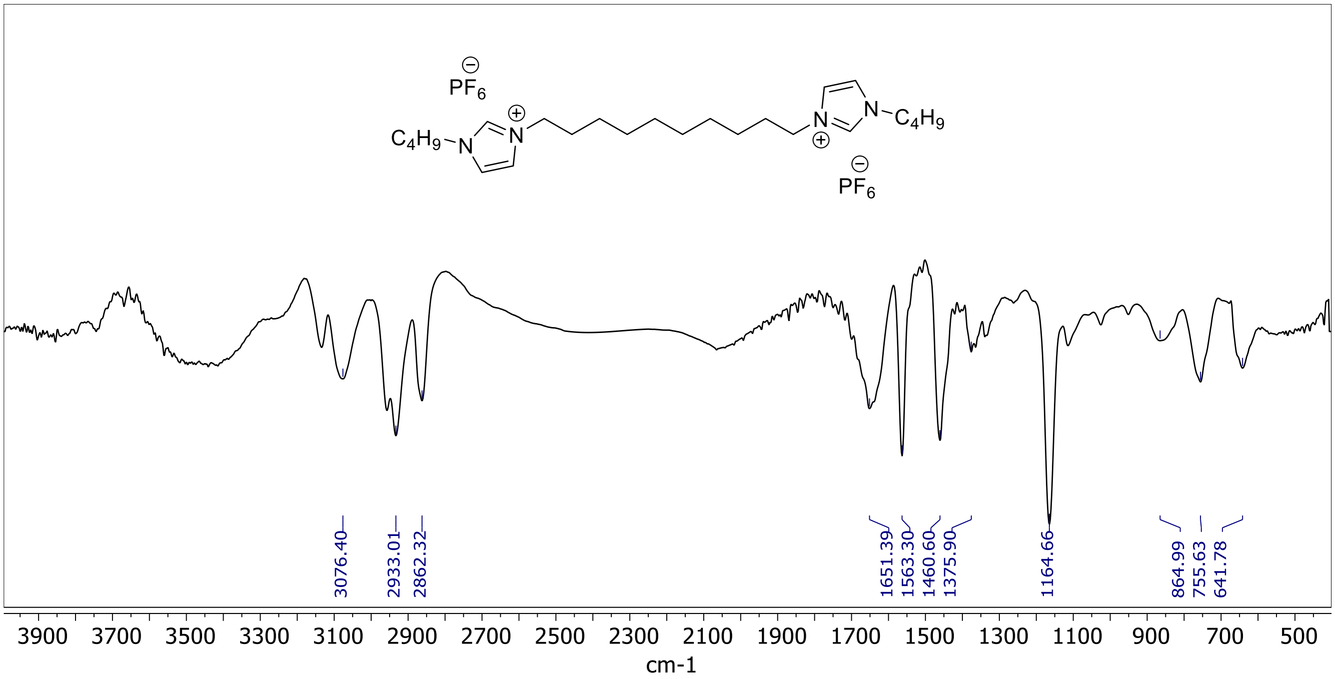

Fig. S12. FT-IR spectrum of 1,10-*bis*[Bim][PF_6] (**2d**)

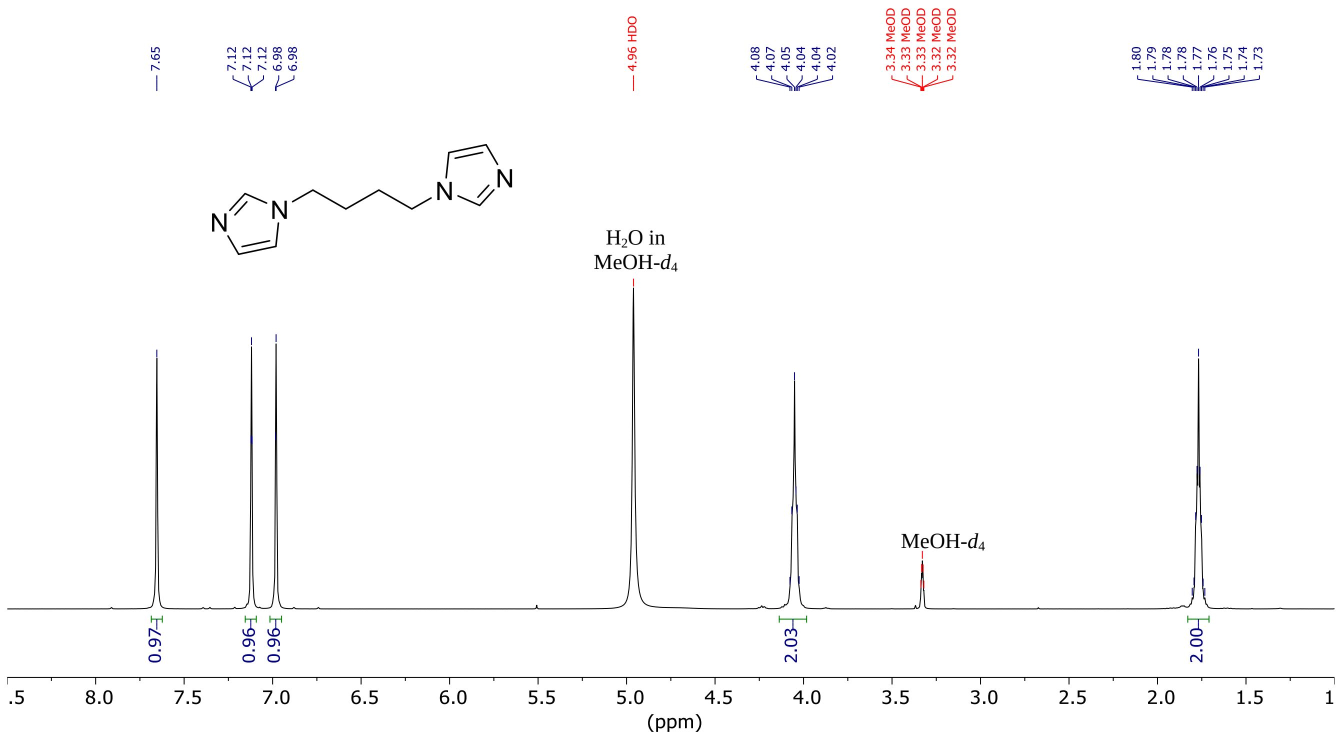

Fig. S13. ^{1}H-NMR spectrum of 1,4-*bis*[Bim] (**6a**) in MeOH-d_4

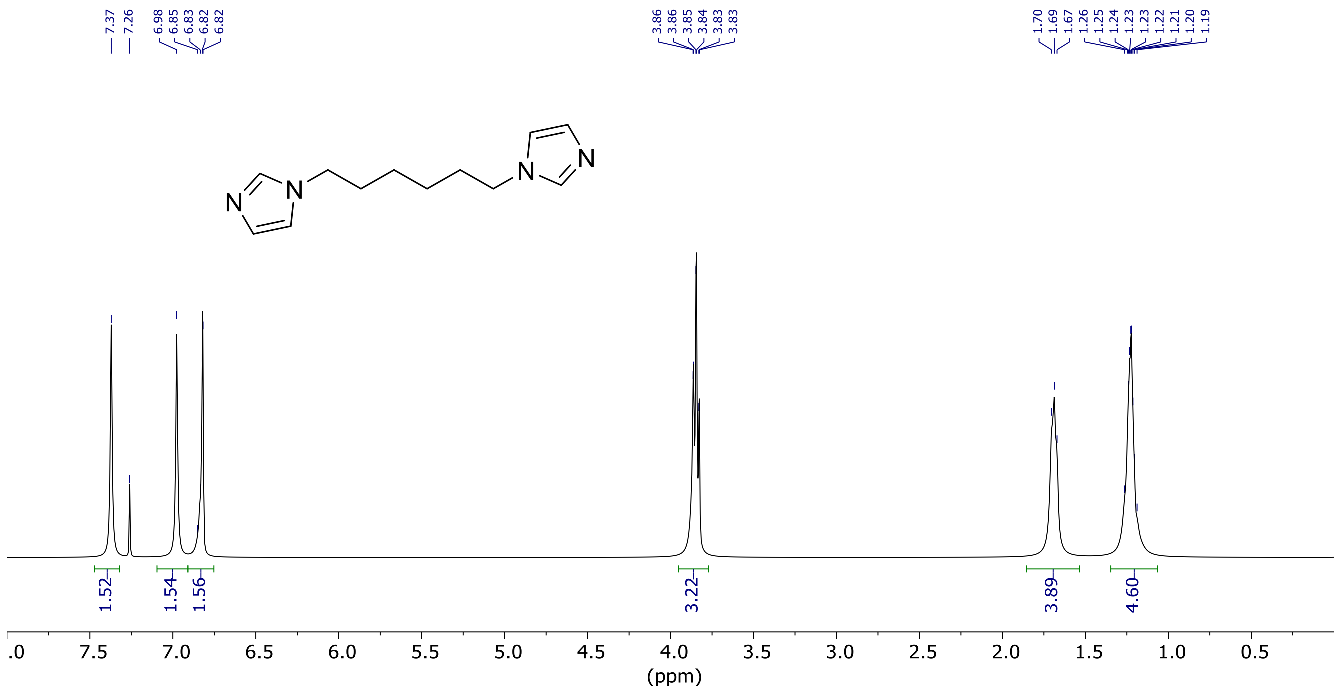

Fig. S14. ^{1}H-NMR spectrum of 1,6-*bis*[Bim] (**6b**) in $CDCl_3$

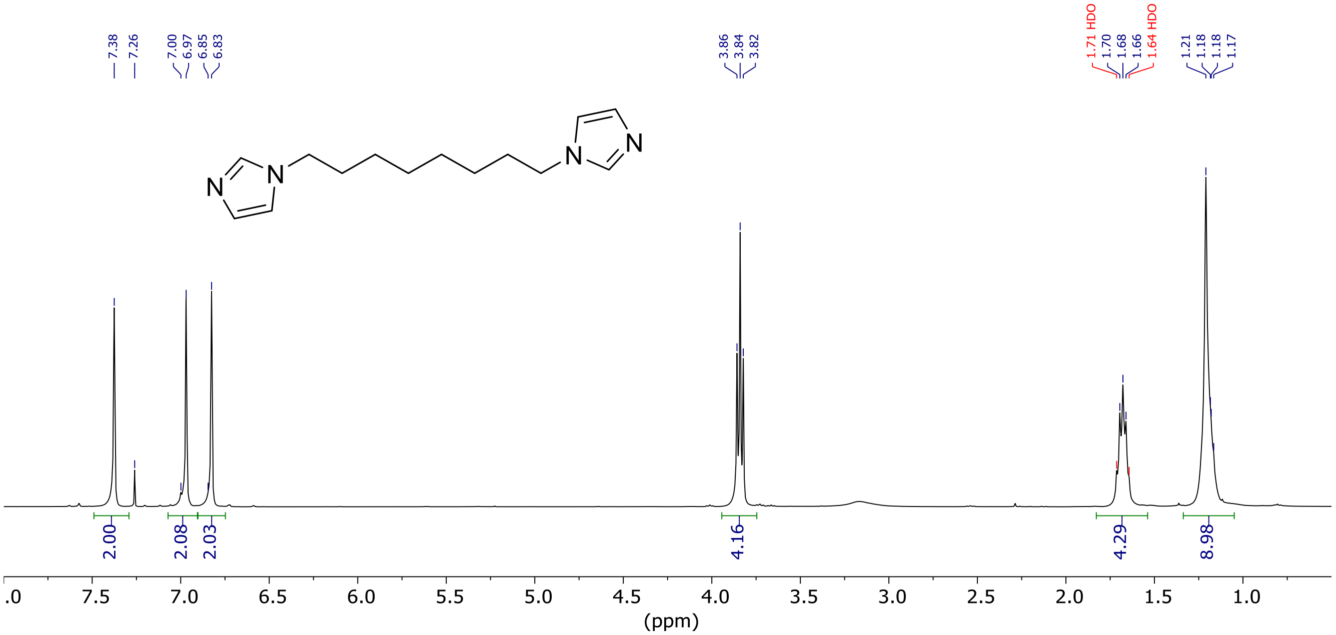

Fig. S15. ^{1}H-NMR spectrum of 1,8-*bis*[Bim] (**6c**) in $CDCl_3$

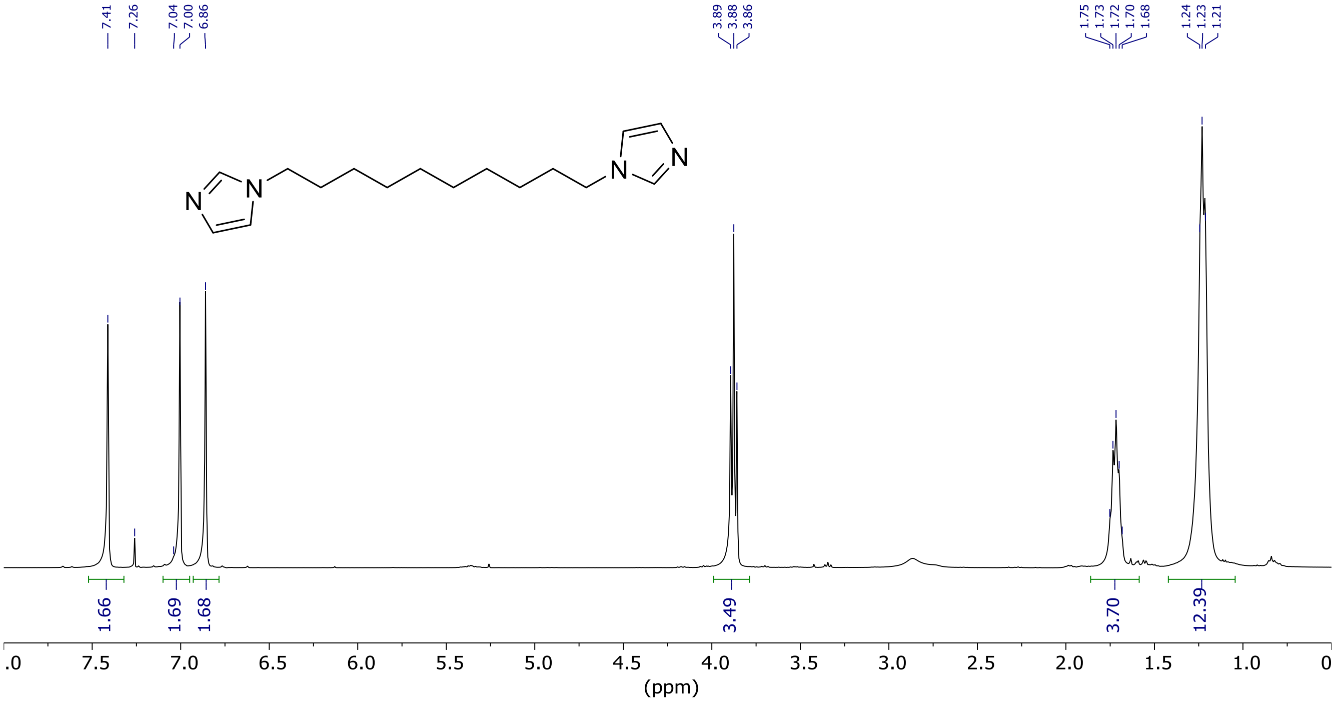

Fig. S16. ^{1}H-NMR spectrum of 1,10-*bis*[Bim] (**6d**) in $CDCl_3$

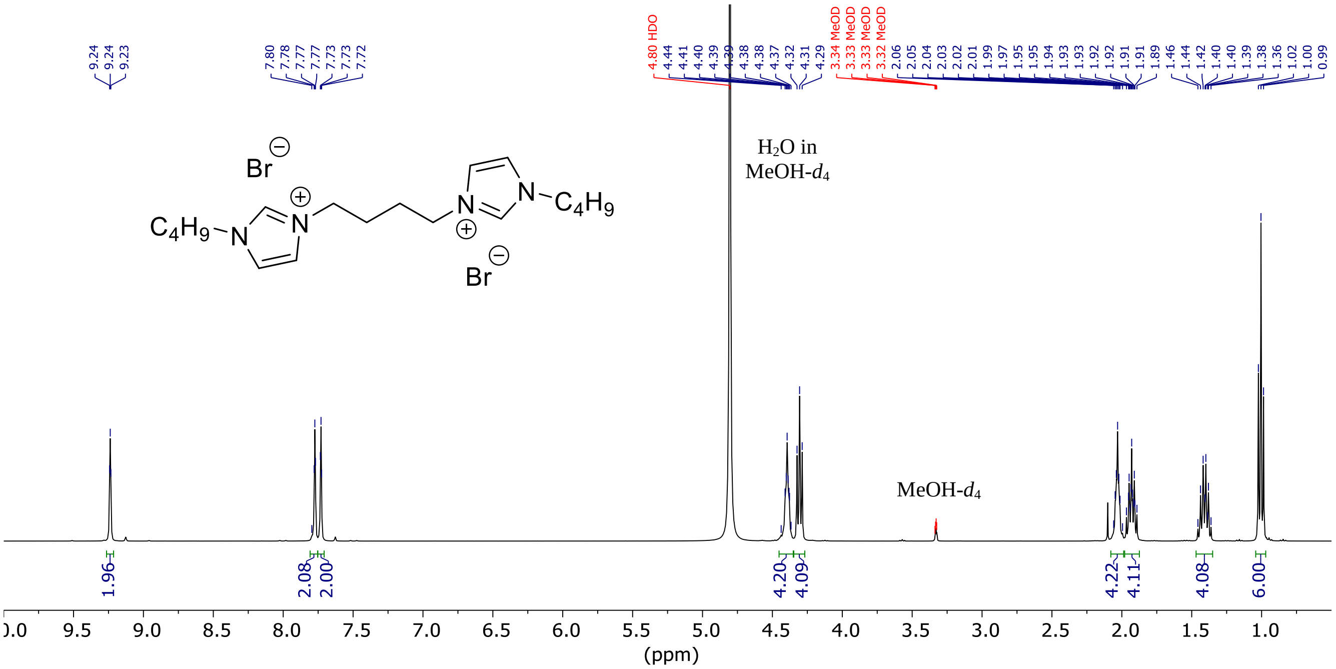

Fig. S17. ^{1}H-NMR spectrum of 1,4-*bis*[Bim][Br] (**7a**) in MeOH-d_4

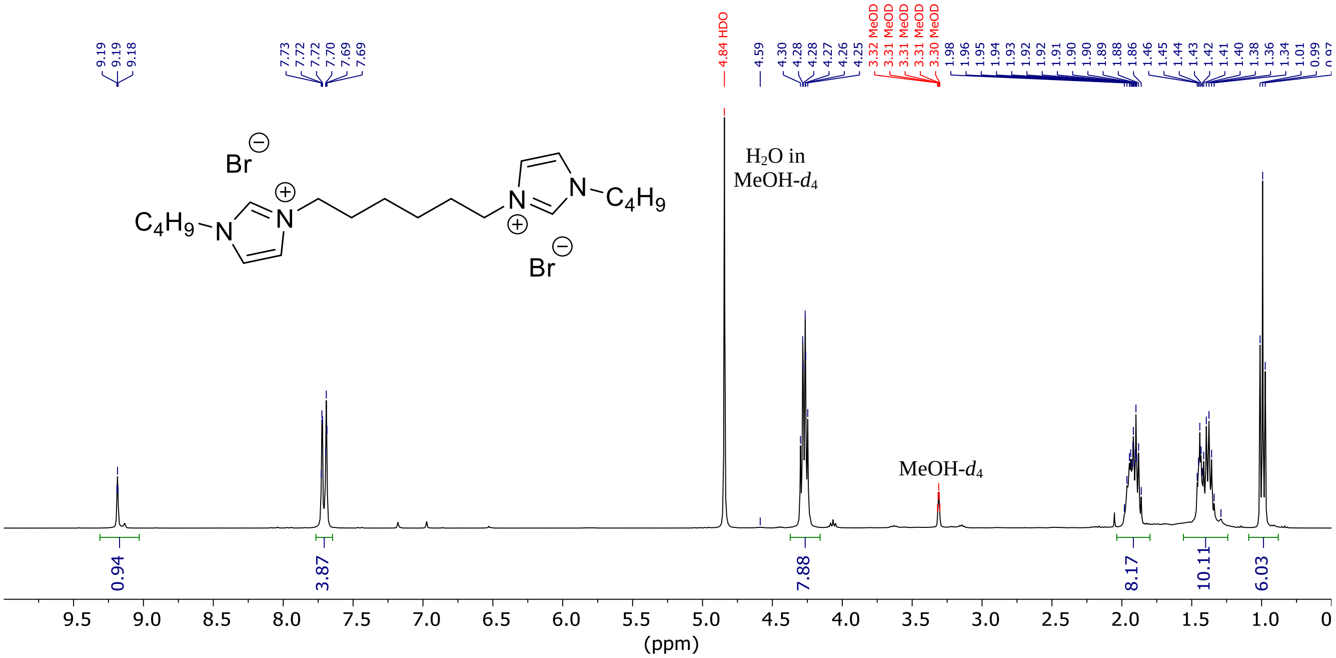

Fig. S18. ^{1}H-NMR spectrum of 1,6-*bis*[Bim][Br] (**7b**) on MeOH-d_4

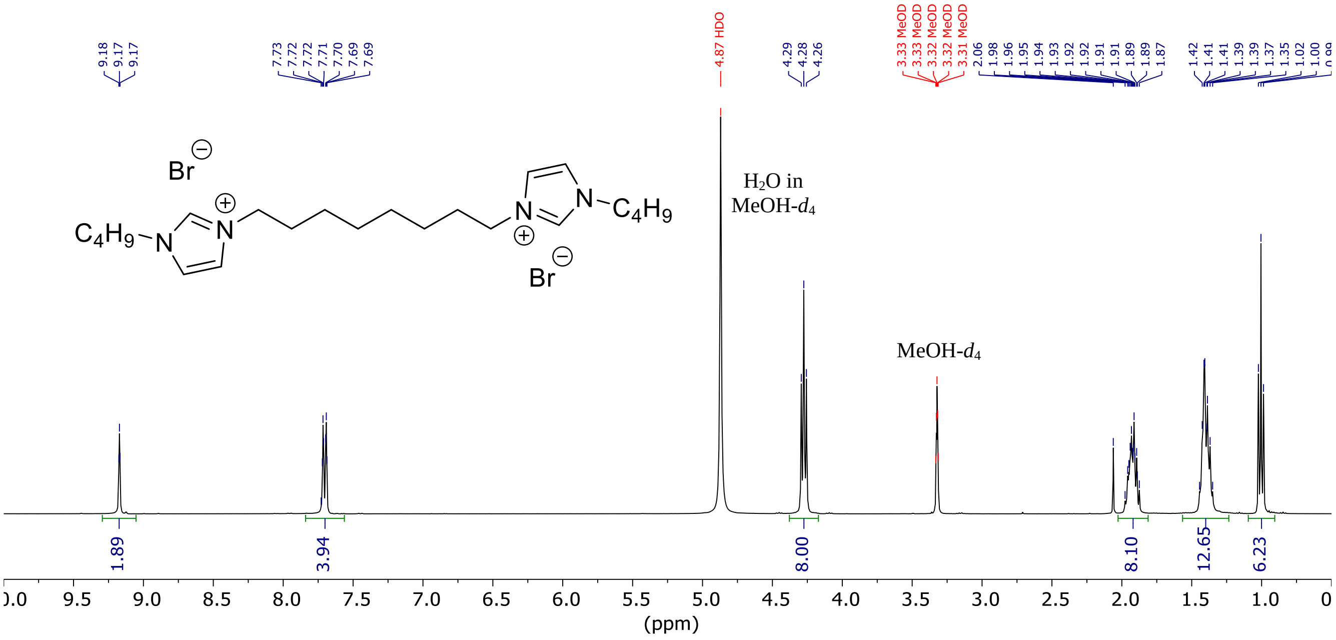

Fig. S19. ^{1}H-NMR spectrum of 1,8-*bis*[Bim][Br] (**7c**) in MeOH-d_4

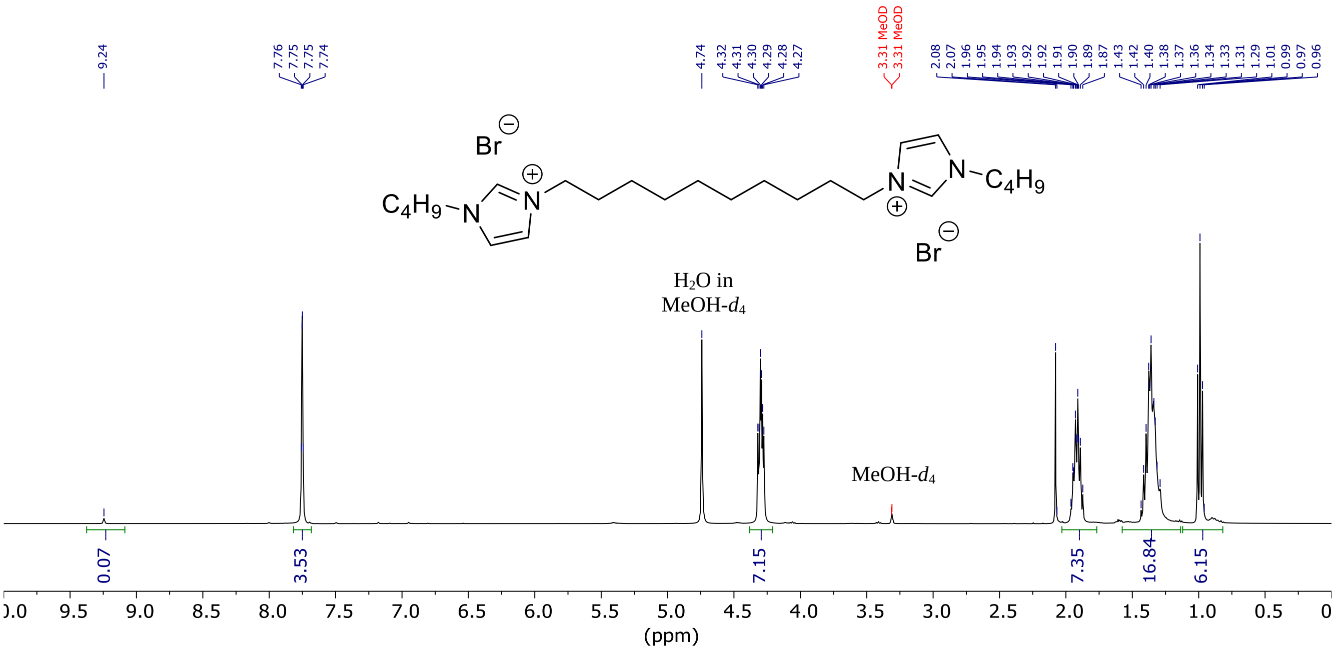

Fig. S20. ^{1}H-NMR spectrum of 1,10-*bis*[Bim][Br] (**7d**) in MeOD-d_4

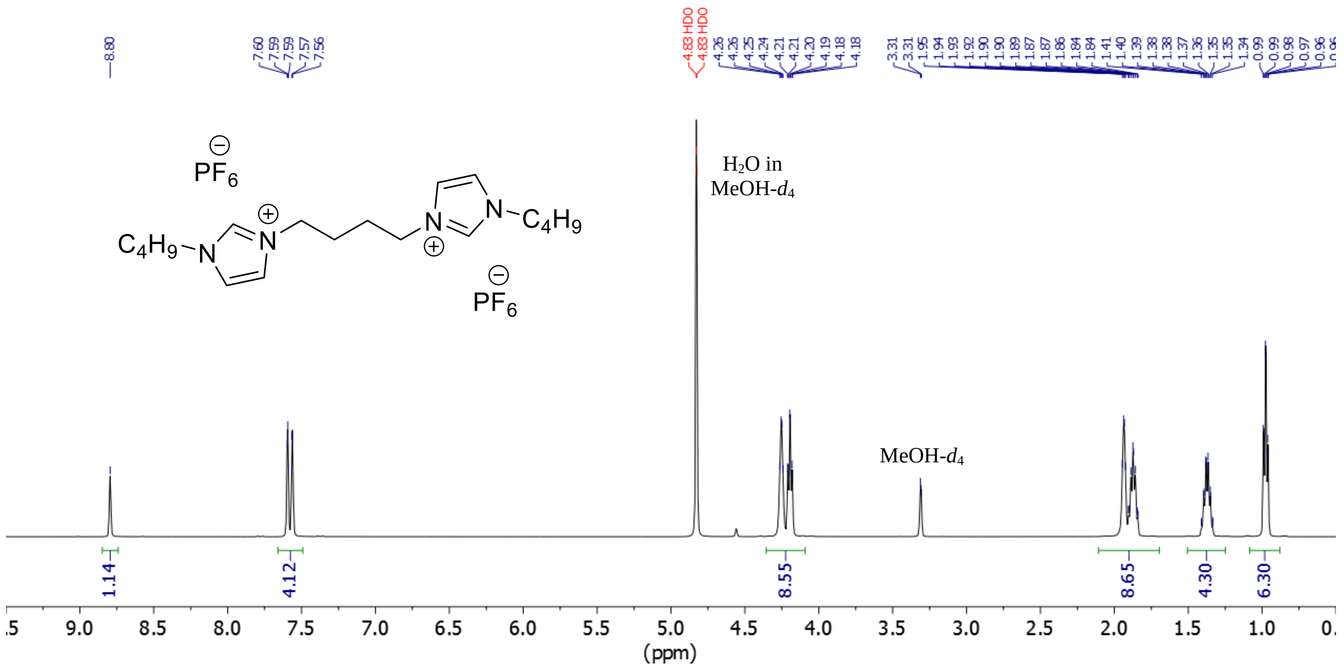

Fig. S21. ^{1}H-NMR spectrum of 1,4-*bis*[Bim][PF_6] (**2a**) in MeOH-d_4

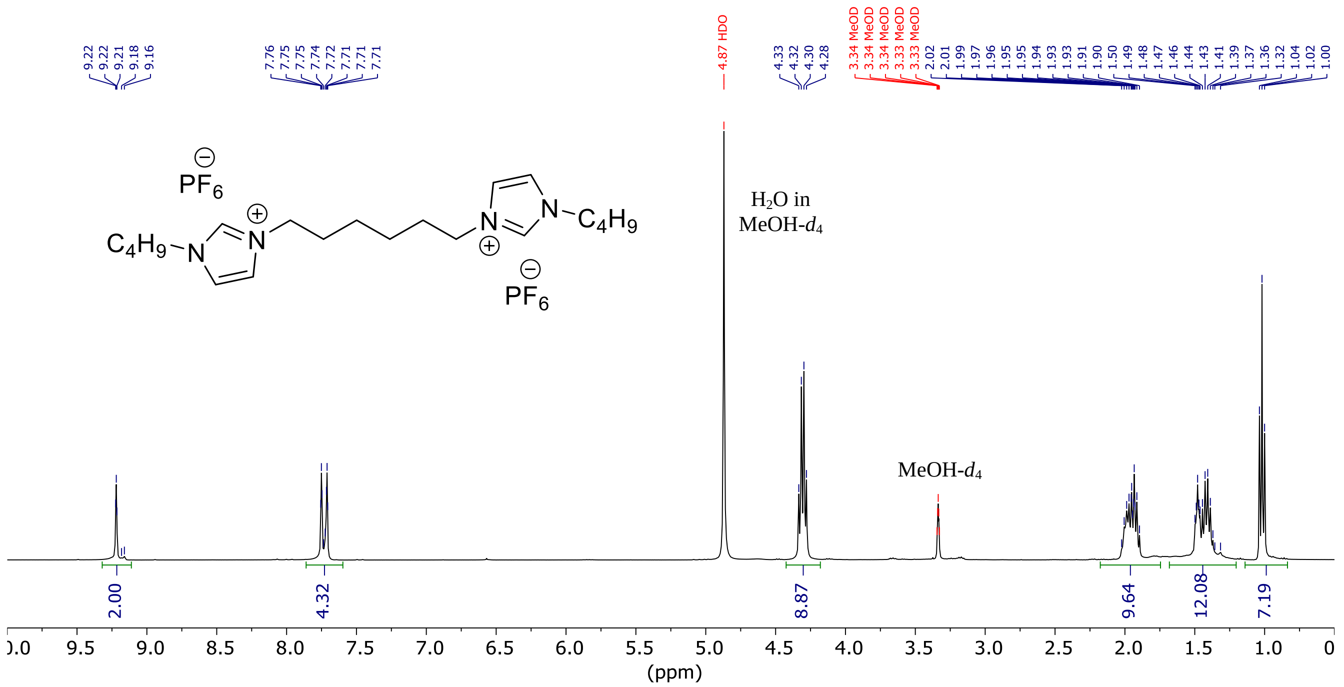

Fig. S22. ^{1}H-NMR spectrum of 1,6-*bis*[Bim][PF_6] (**2b**) in MeOD-d_4

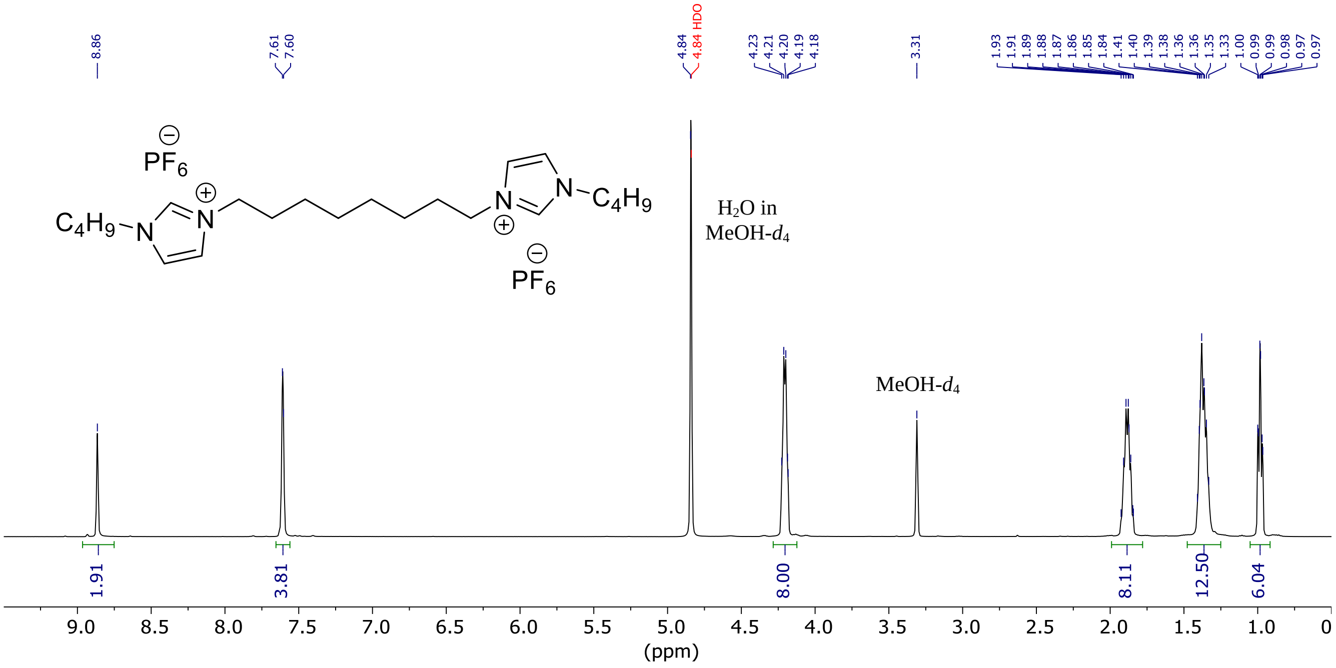

Fig. S23. ^{1}H-NMR spectrum of 1,8-*bis*[Bim][PF_6] (**2c**) in MeOH-d_4

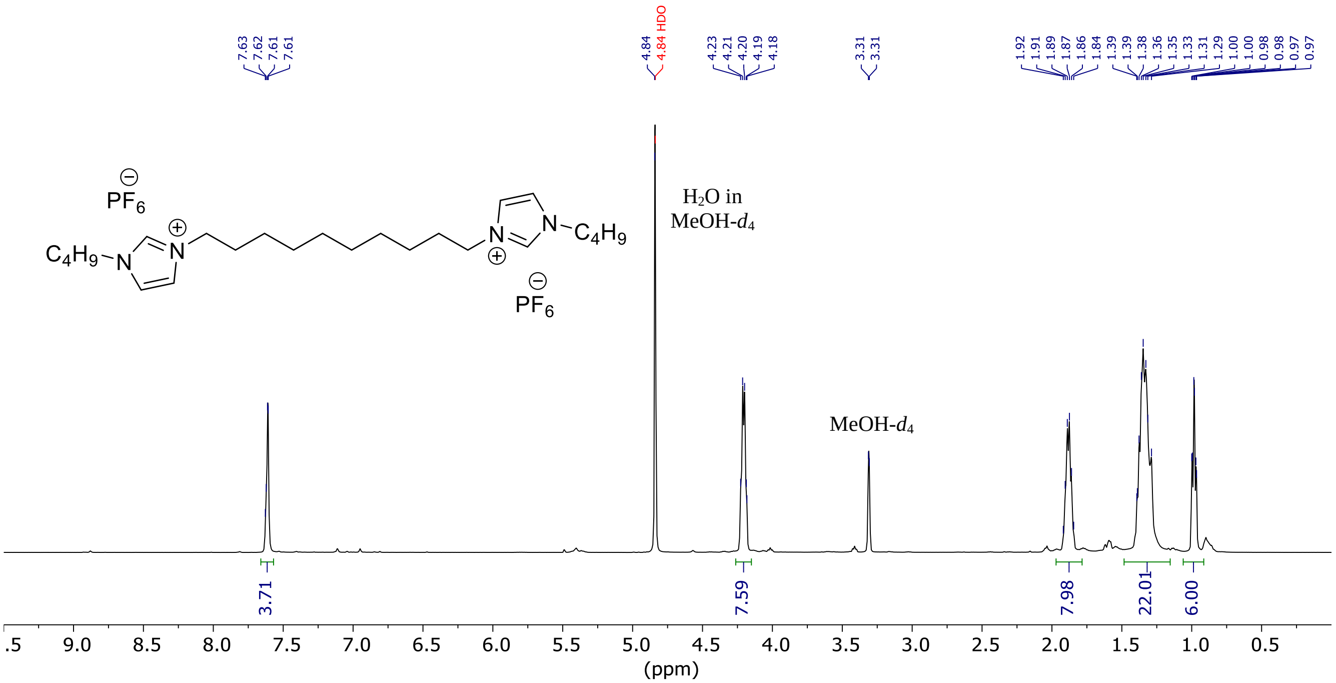

Fig. S24. ^{1}H-NMR spectrum of 1,10-*bis*[Bim][PF_6] (**2d**) in MeOH-d_4

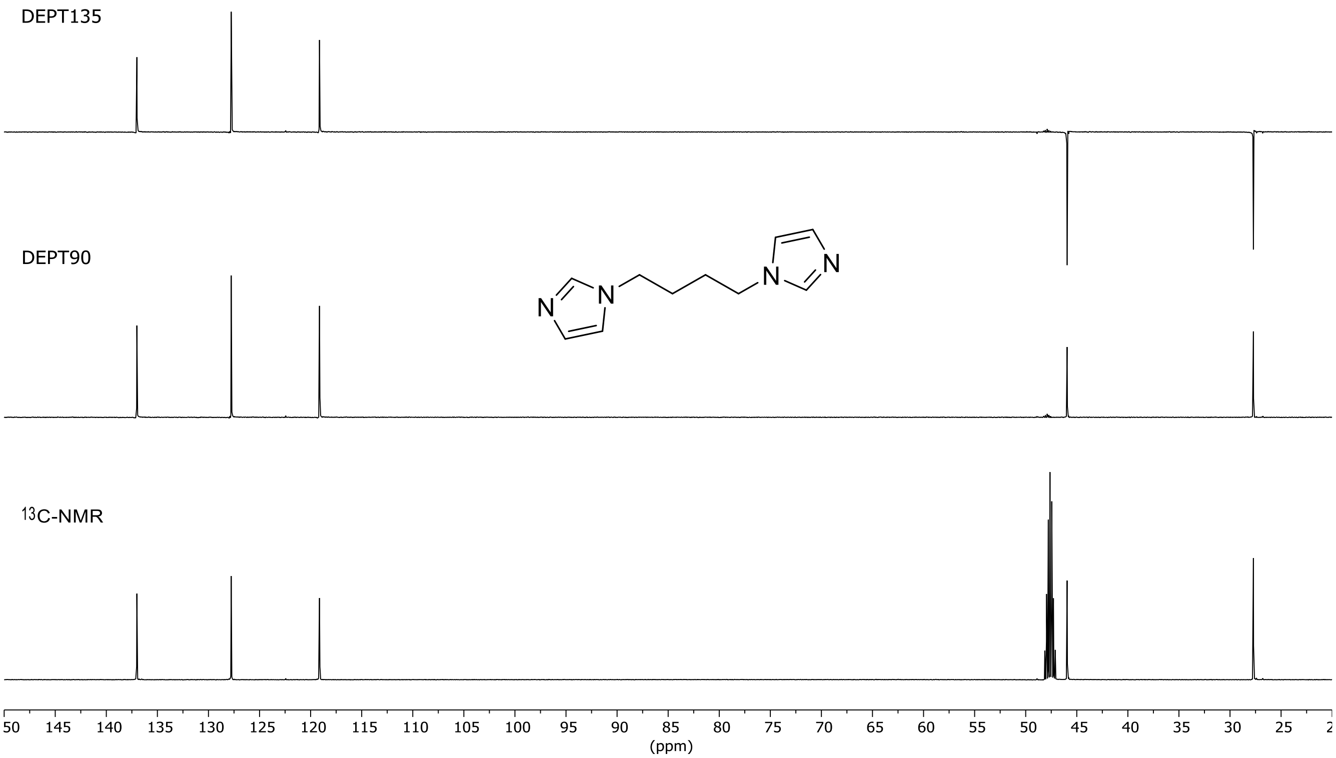

Fig. S25. ^{13}C-NMR spectrum of 1,4-*bis*[Bim] (**6a**) in MeOH-d_4

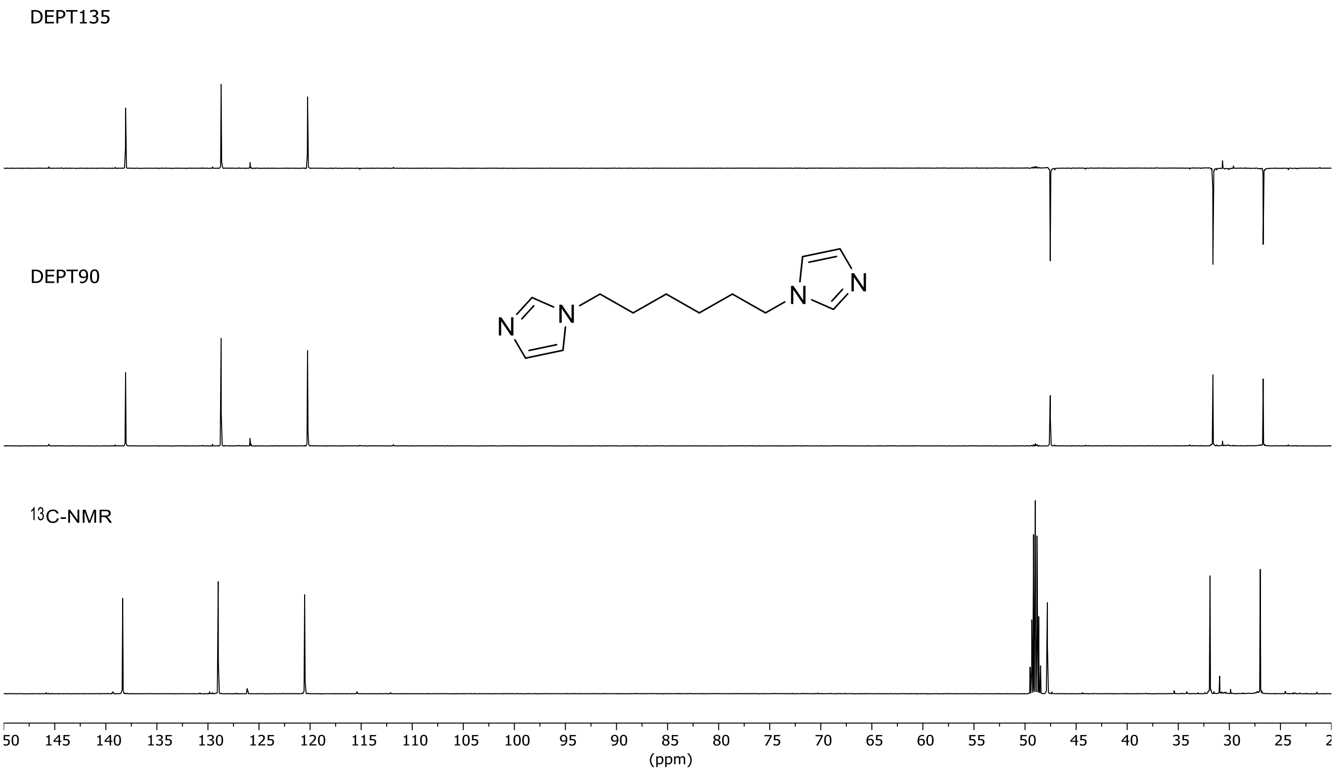

Fig. S26. ^{13}C-NMR spectrum of 1,6-*bis*[Bim] (**6b**) in MeOH-d_4

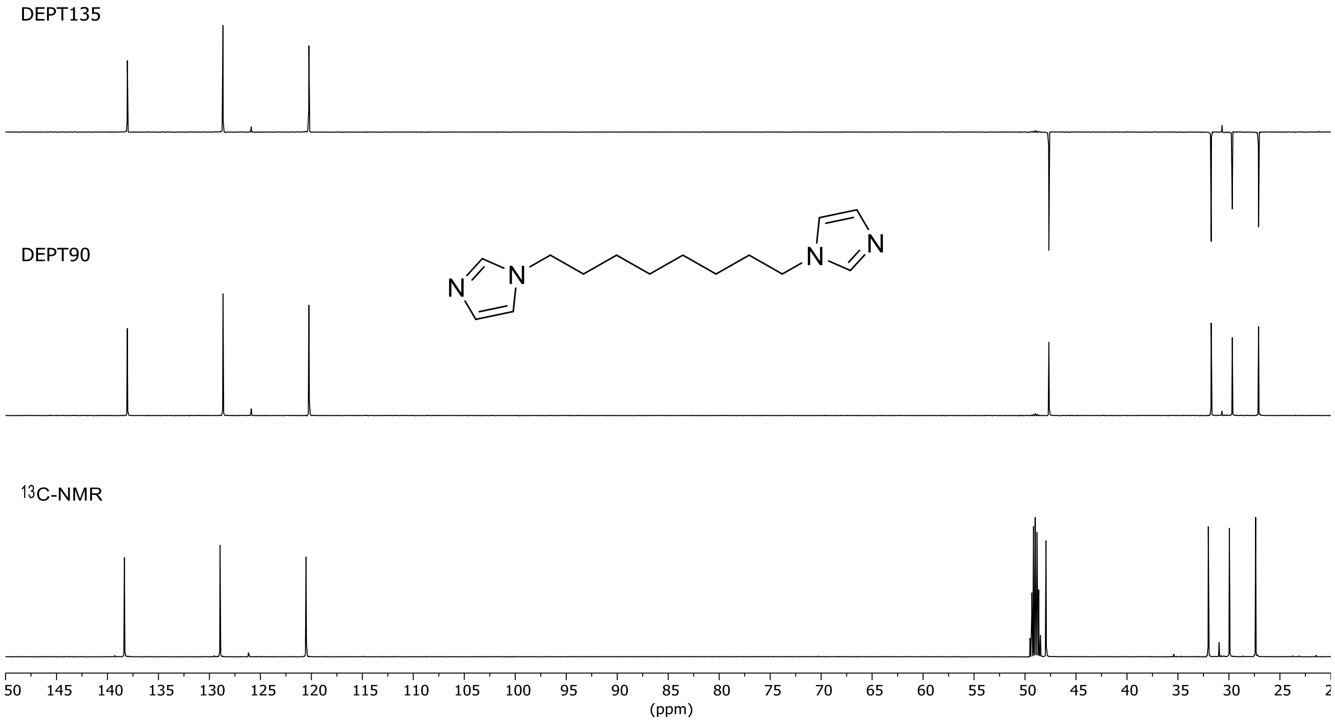

Fig. S27. ^{13}C-NMR spectrum of 1,8-*bis*[Bim] (**6c**) in MeOH-d_4

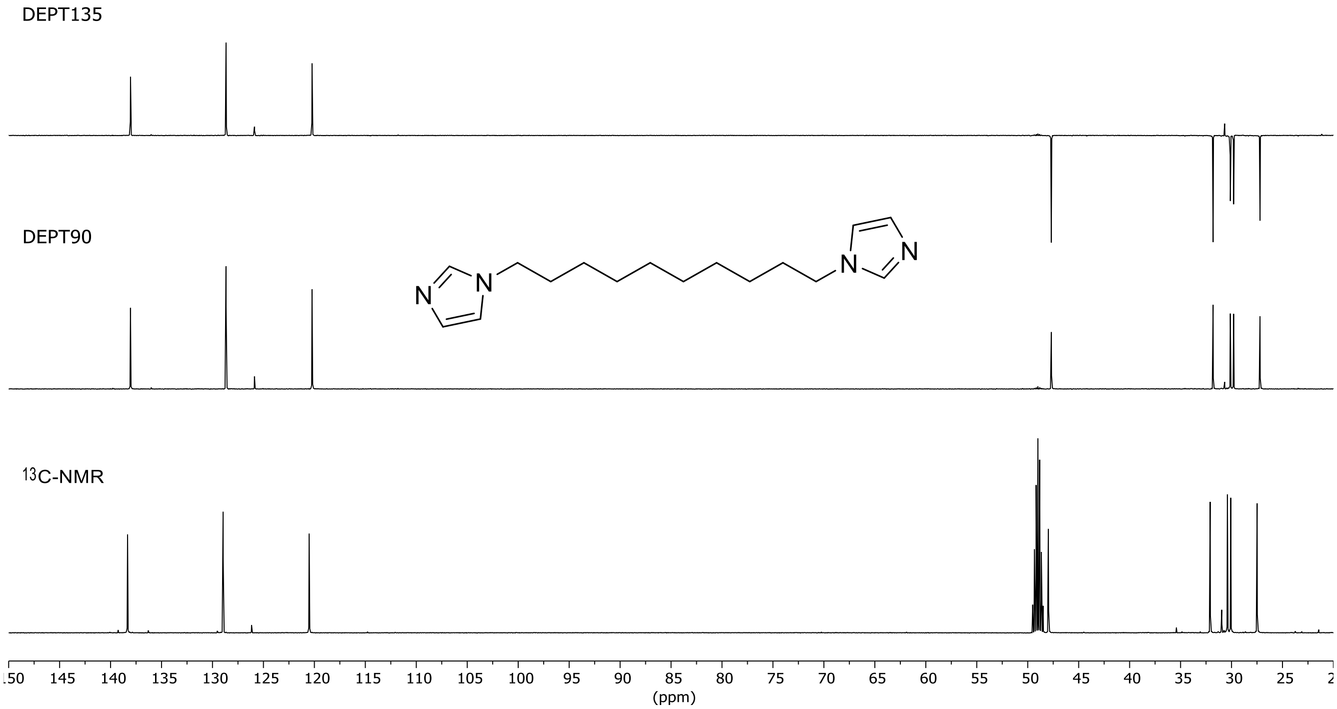

Fig. S28. ^{13}C-NMR spectrum of 1,10-*bis*[Bim] (**6d**) in MeOH-d_4

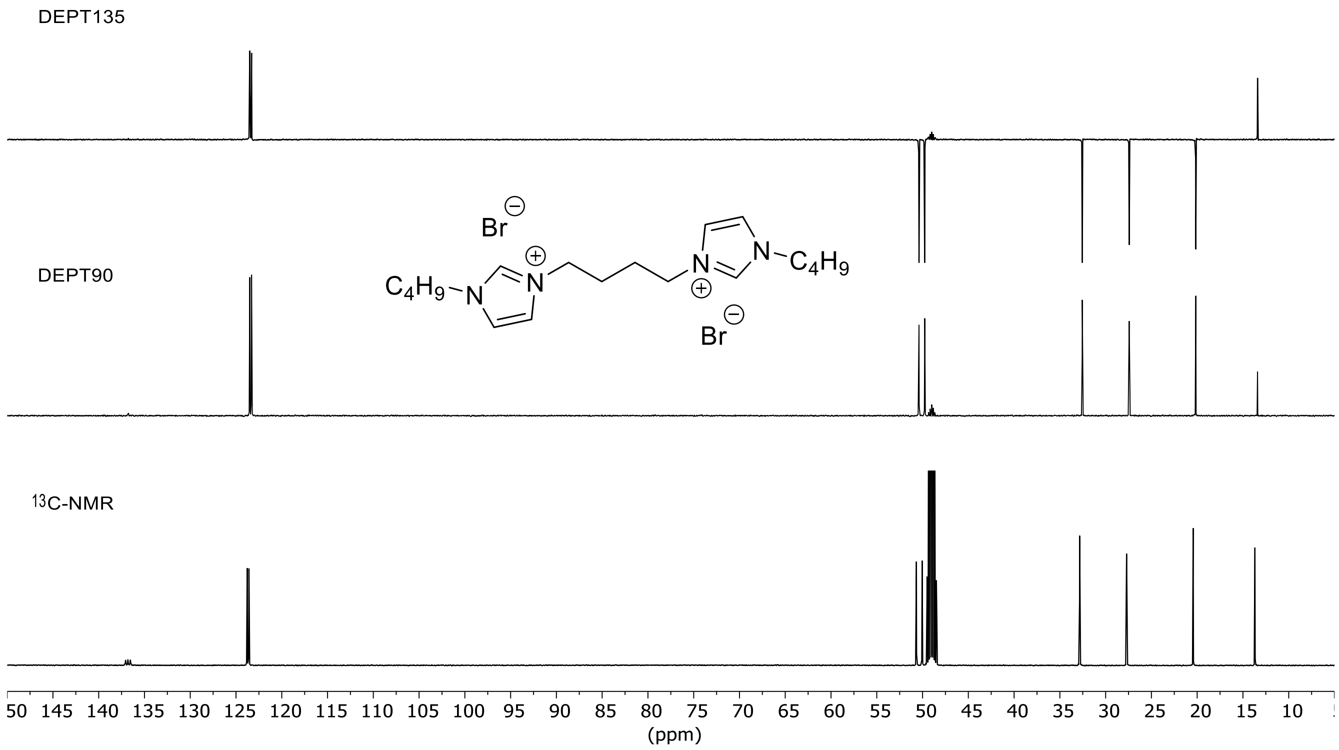

Fig. S29. ^{13}C-NMR spectrum of 1,4-*bis*[Bim][Br] (**7a**) in MeOH-d_4

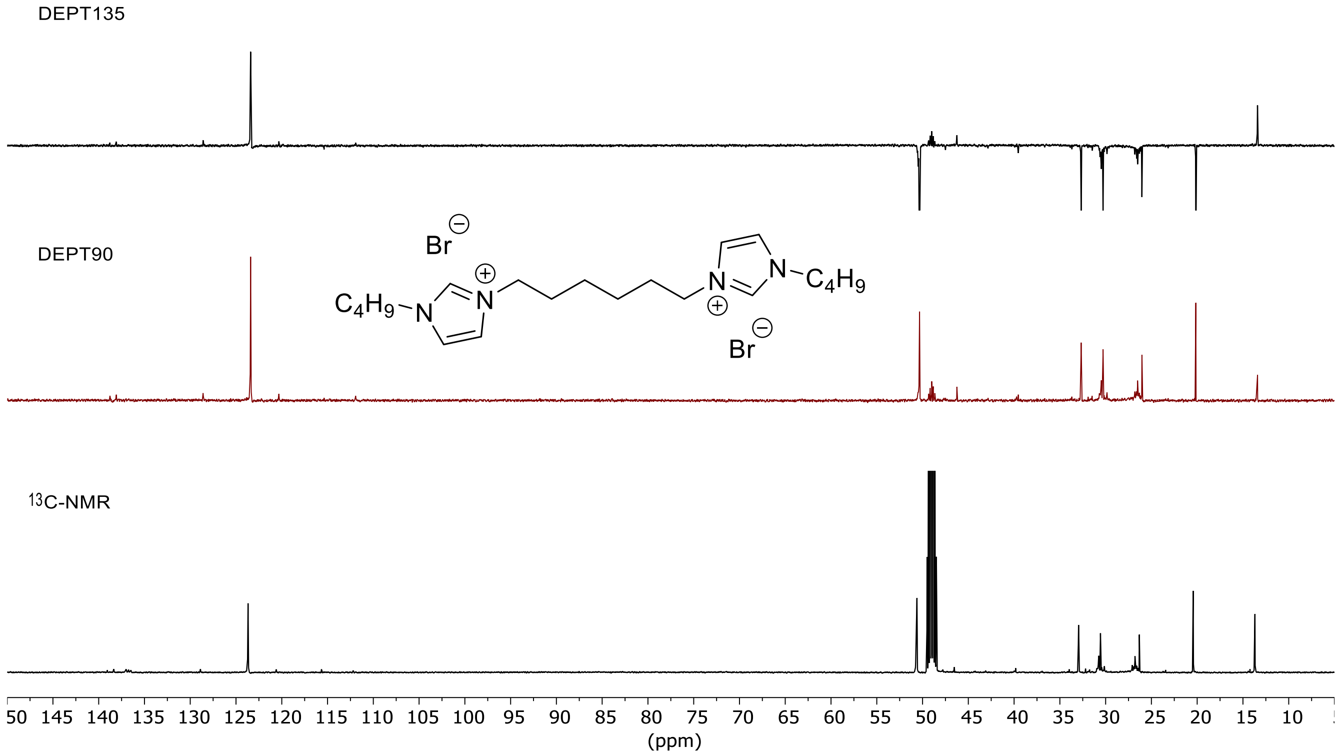

Fig. S30. ^{13}C-NMR spectrum of 1,6-*bis*[Bim][Br] (**7b**) in MeOH-d_4

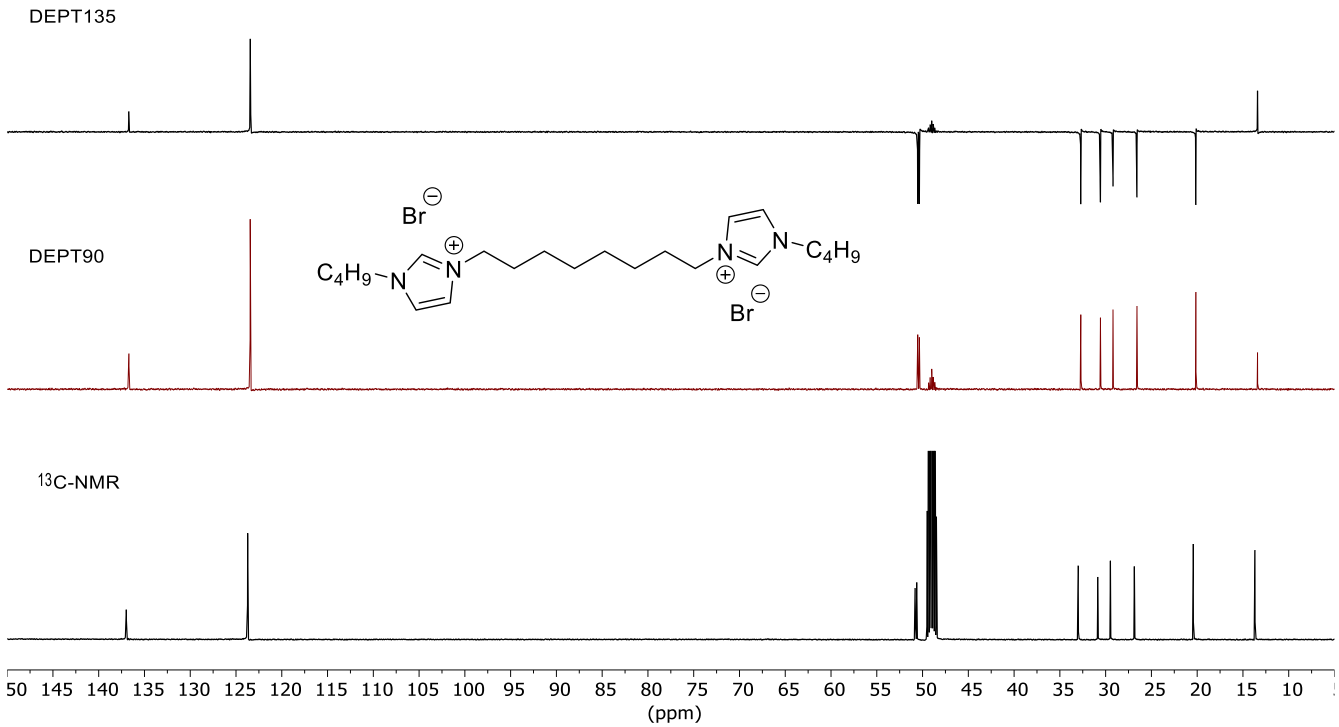

Fig. S31. ^{13}C-NMR spectrum of 1,8-*bis*[Bim][Br] (**7c**) in MeOH-d_4

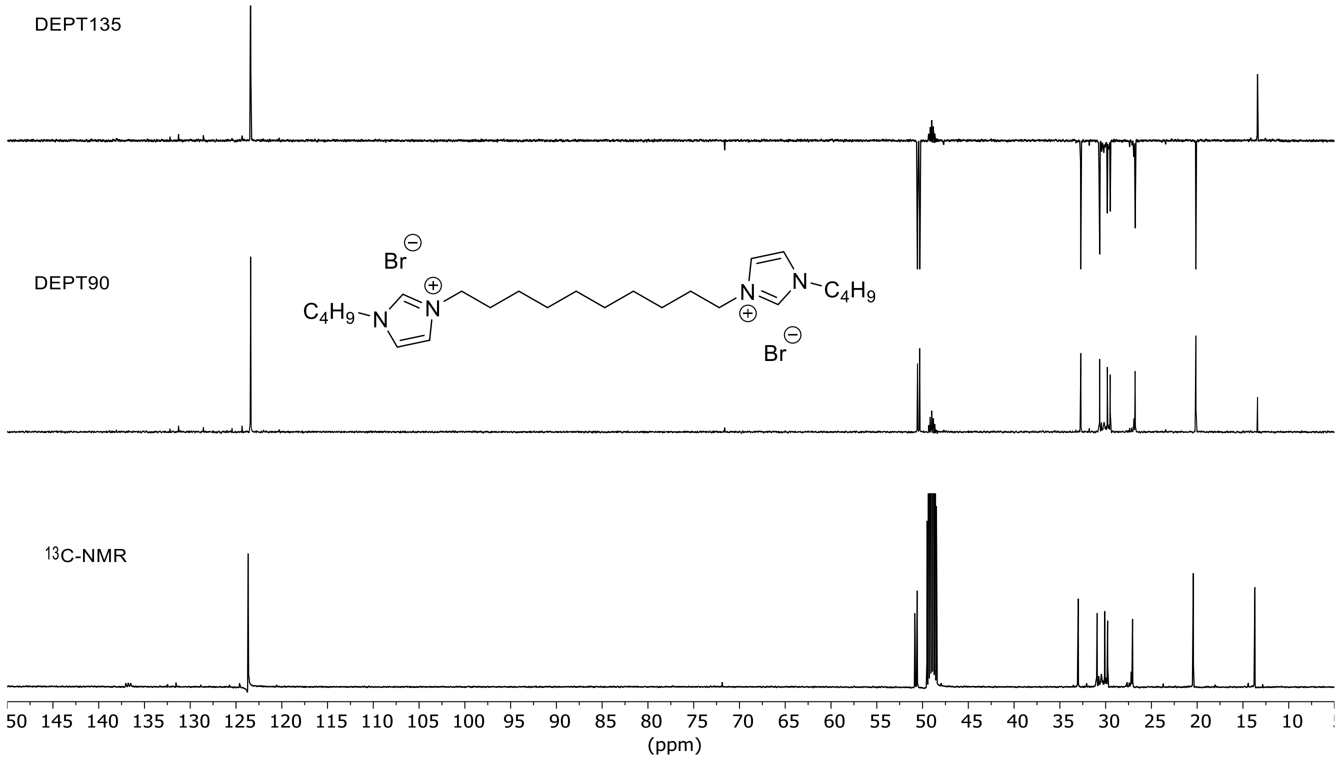

Fig. S32. ^{13}C-NMR spectrum of 1,10-*bis*[Bim][Br] (**7d**) in MeOH-d_4

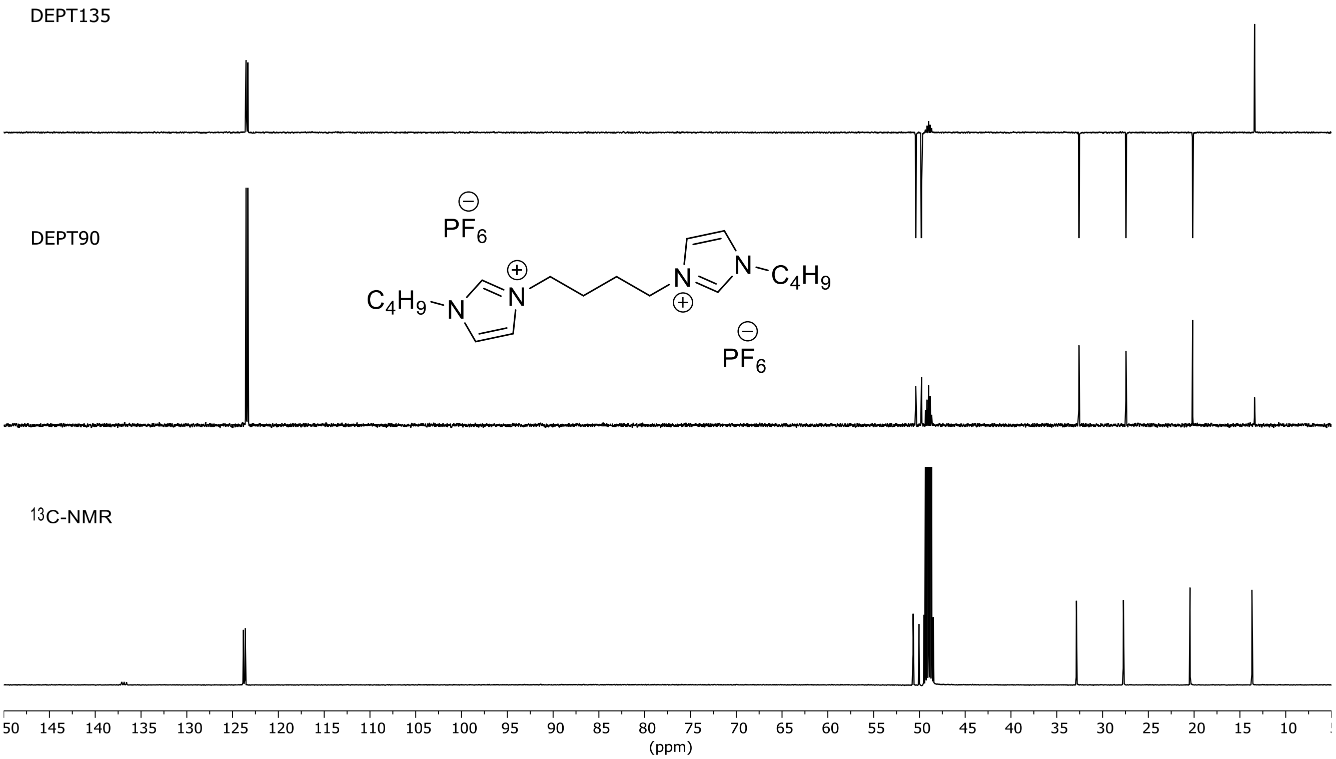

Fig. S33. ^{13}C-NMR spectrum of 1,4-*bis*[Bim][PF_6] (**2a**) in MeOH-d_4

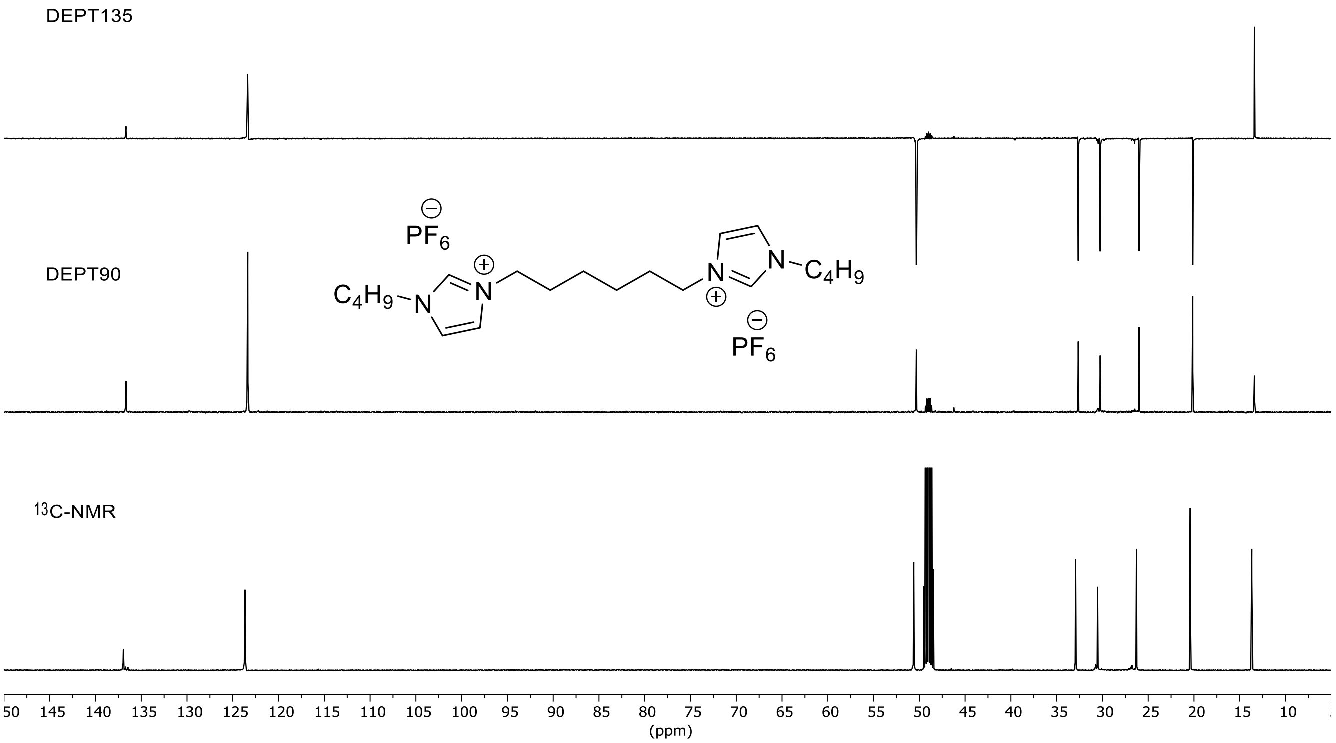

Fig. S34. ^{13}C-NMR spectrum of 1,6-*bis*[Bim][PF_6] (**2b**) in MeOH-d_4

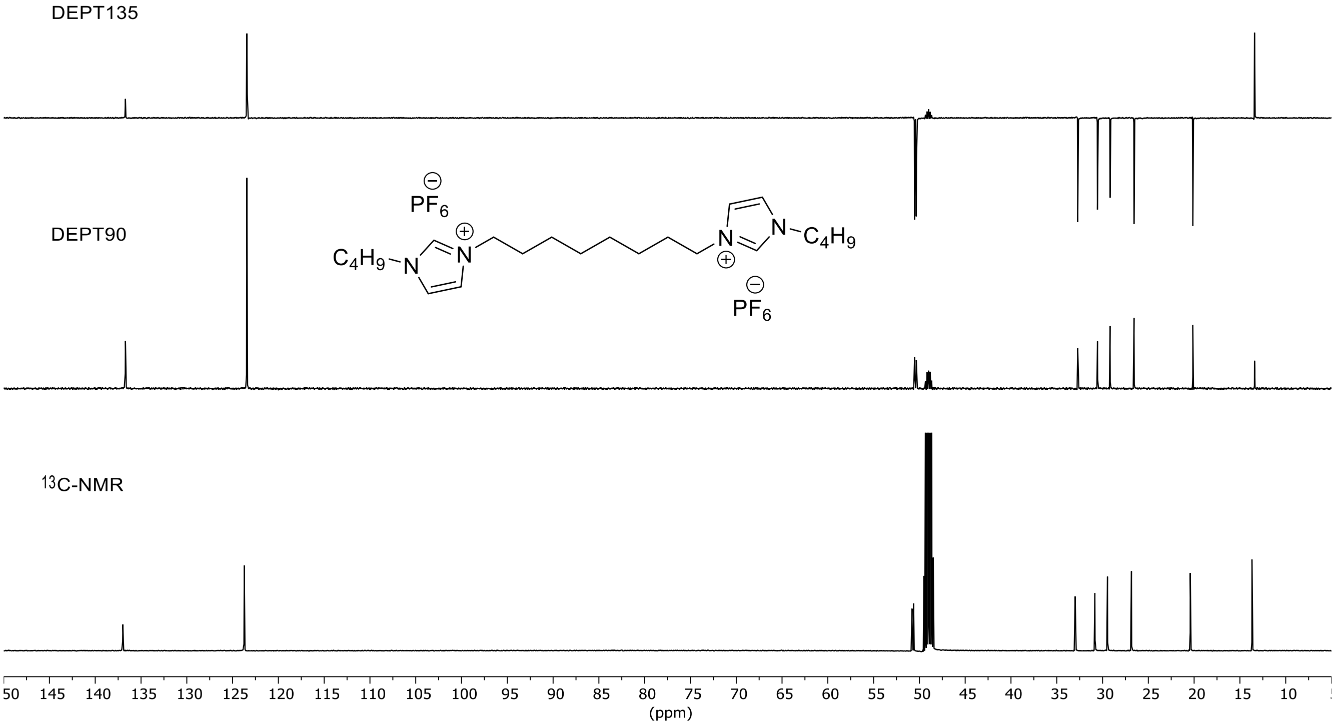

Fig. S35. ^{13}C-NMR spectrum of 1,8-*bis*[Bim][PF$_6$] (**2c**) in MeOH-d_4

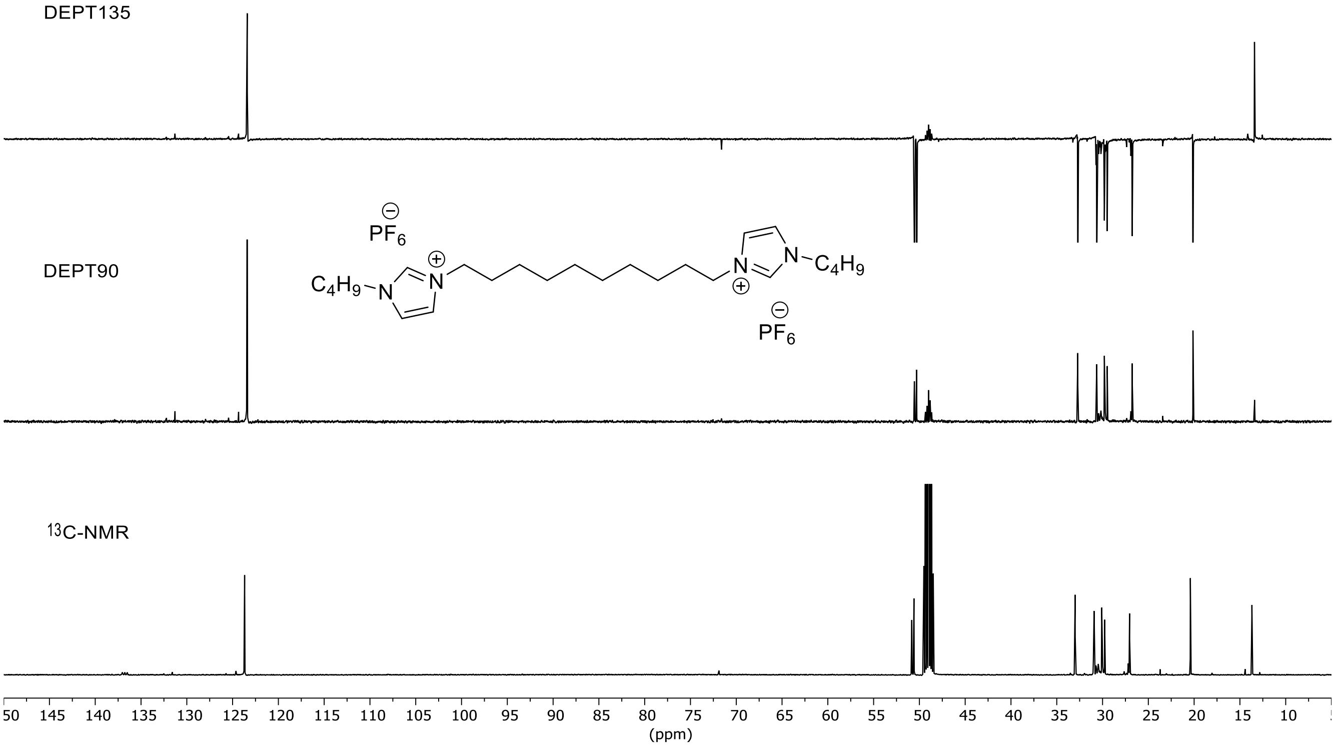

Fig. S36. ^{13}C-NMR spectrum of 1,10-*bis*[Bim][PF$_6$] (**2d**) in MeOH-d_4

2. Kinetic studies of the ROP of *ε*-caprolactone catalyzed by the synthesized DILs catalysts with *n*-dodecanol initiator

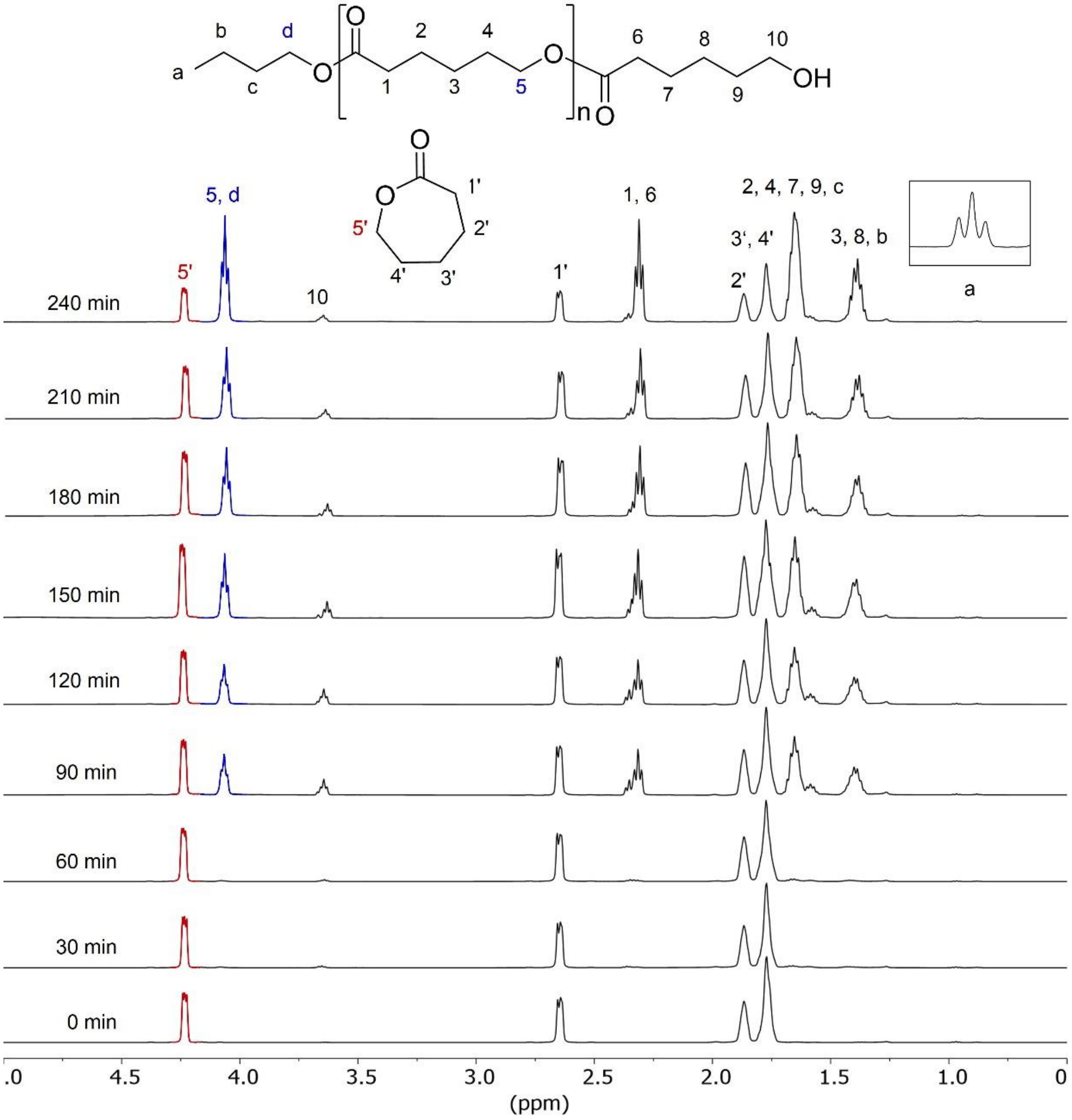

Fig. S37. ^{1}H-NMR spectrum of kinetic studies the ROP of CL using 1,4-*bis*[Bim][PF_6] (**2a**) as catalyst with 1.0 mol% of 1-dodecanol at 150 °C.

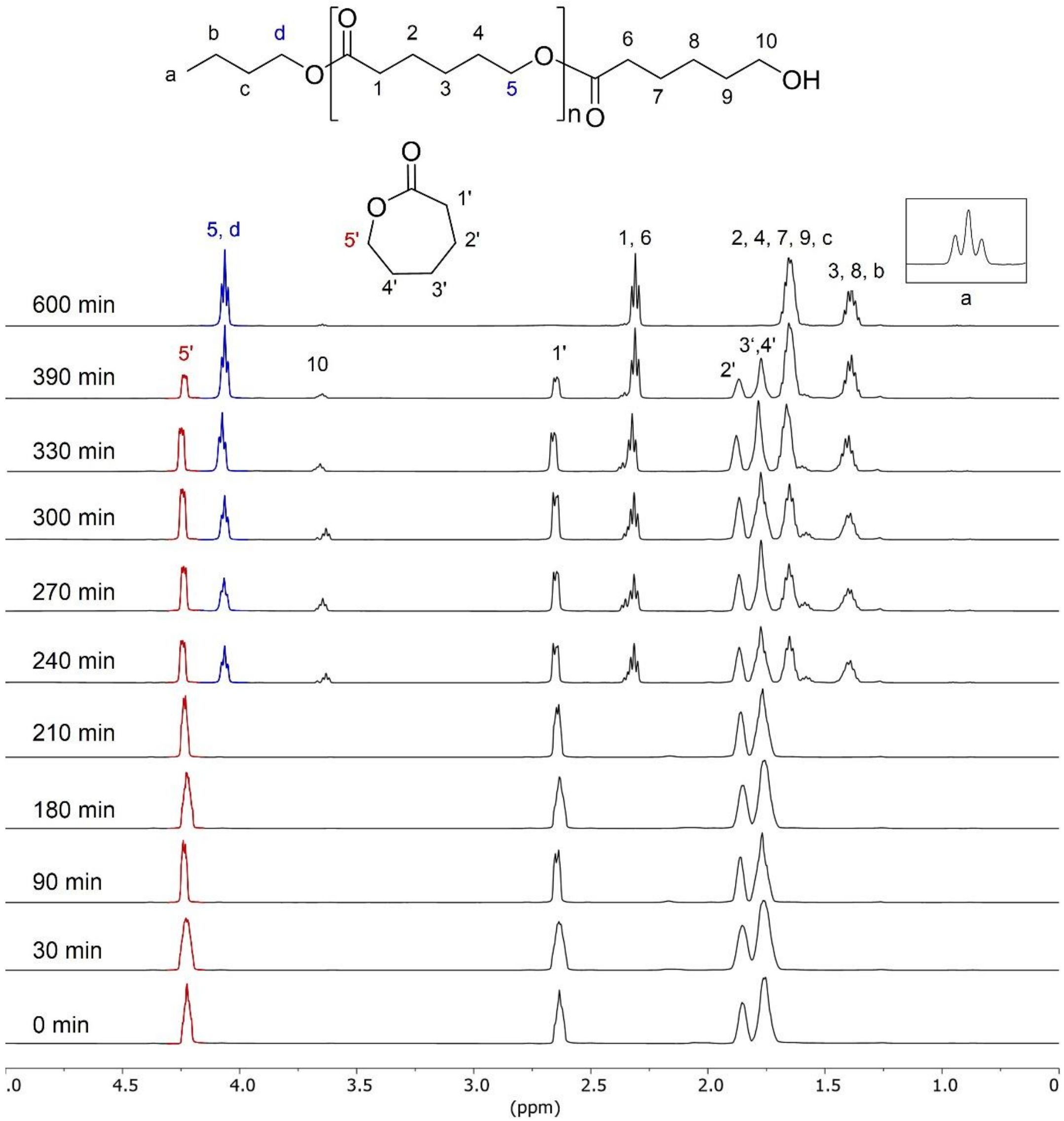

Fig. S38. ^{1}H-NMR spectrum of kinetic studies the ROP of CL using 1,6-*bis*[Bim][PF_6] (**2b**) as catalyst with 1.0 mol% of 1-dodecanol at 150 °C.

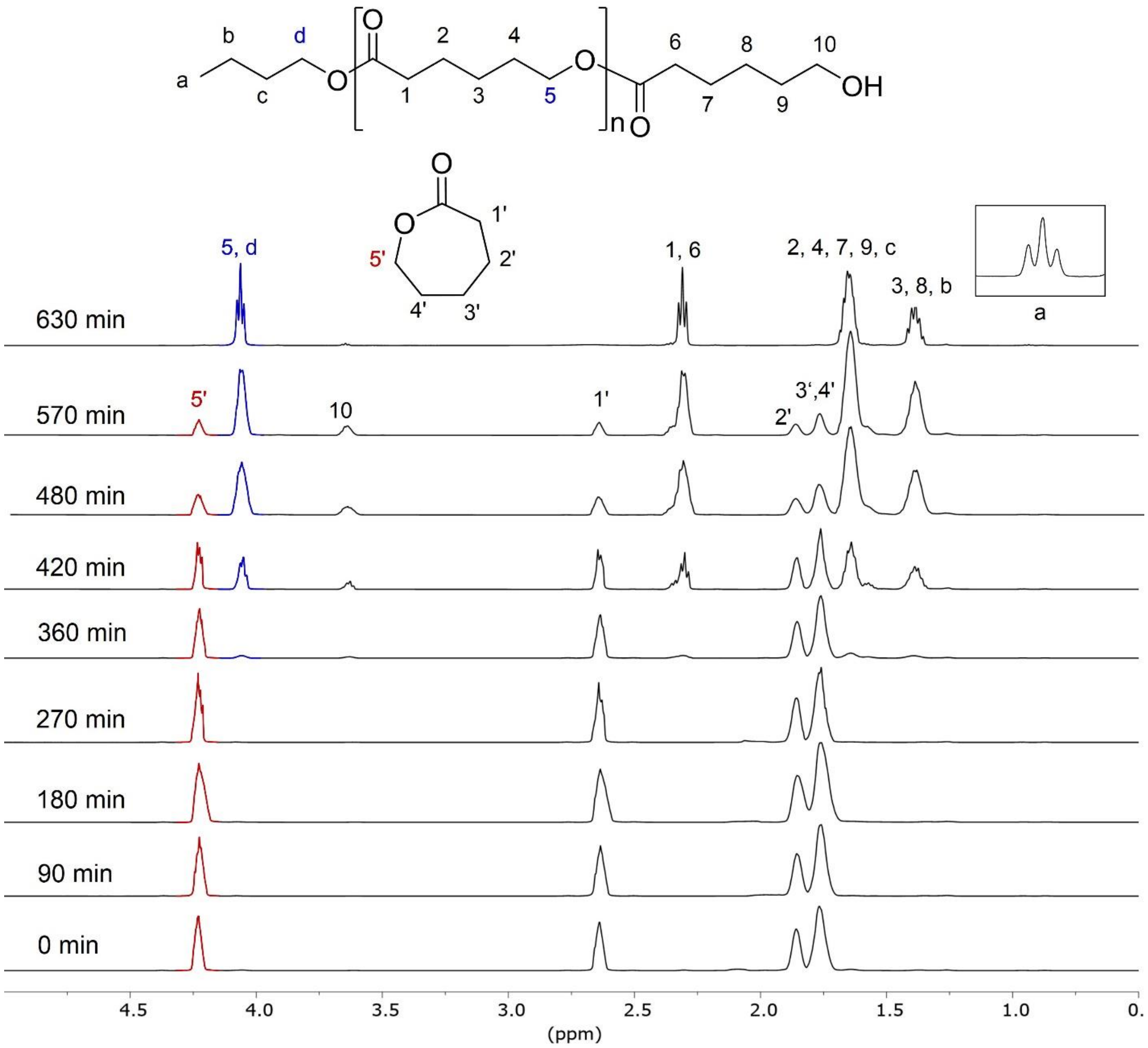

Fig. S39. ^{1}H-NMR spectrum of kinetic studies the ROP of CL using 1,8-*bis*[Bim][PF_6] (**2c**) as catalyst with 1.0 mol% of 1-dodecanol at 150 °C.

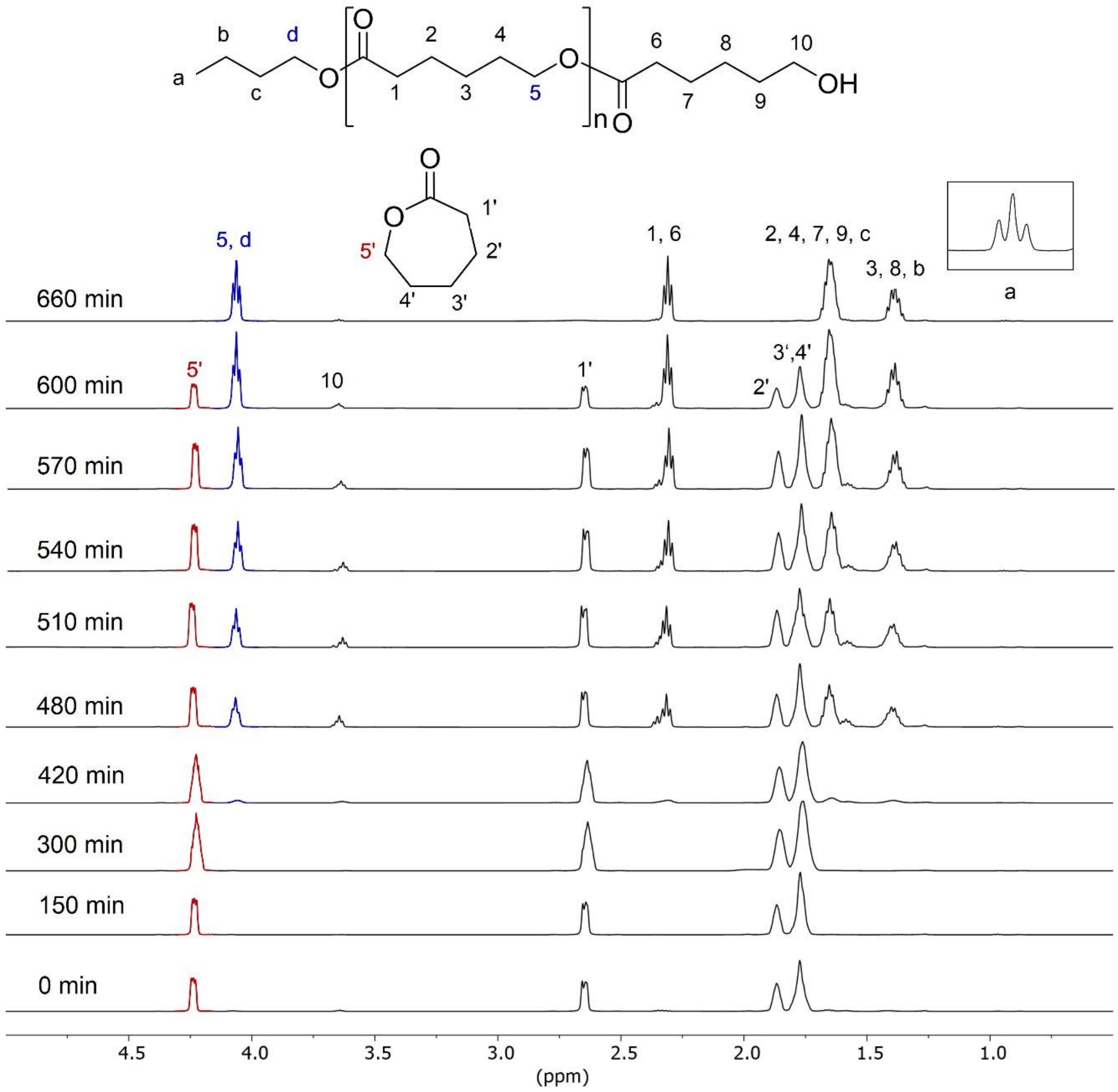

Fig. S40. ^{1}H-NMR spectrum of kinetic studies the ROP of CL using 1,10-*bis*[Bim][PF_6] (**2d**) as catalyst with 1.0 mol% of 1-dodecanol at 150 °C.

3. Differential scanning calorimetry thermogram of synthesized poly(*ε*-caprolactone)

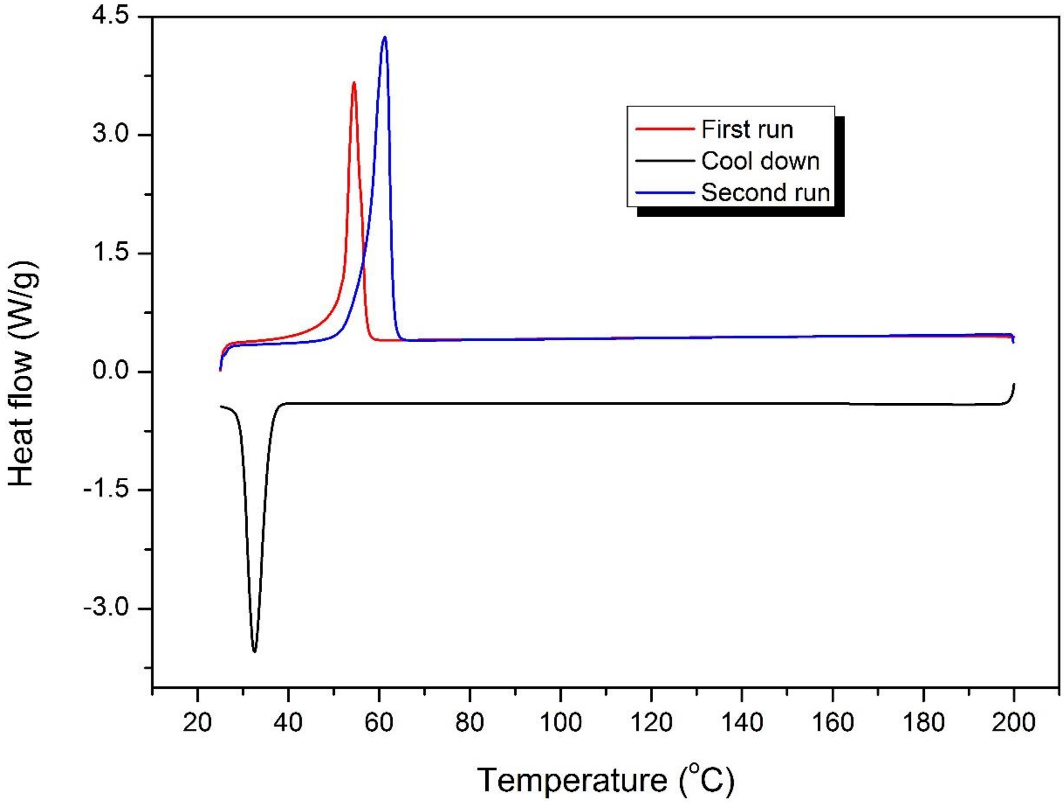

Fig. **S41**. DSC curves of PCL (entry 25) obtained from the ROP of CL catalyzed by 1,4-*bis*[Bim][PF_6] (**2a**) with 1.0 mol% of 1-dodecanol.

4. Table S1. Percentage cell viability obtained from the cytotoxicity testing of the synthesized DILs catalysts and DMSO at a concentration of 100 μg/mL.

Entry	DILs	Concentration	% Cell viability
1	1,4-*bis*[Bim][PF_6] (**2a**)	100 μg/mL	109.050
2	1,6-*bis*[Bim][PF_6] (**2b**)	100 μg/mL	107.391
3	1,8-*bis*[Bim][PF_6] (**2c**)	100 μg/mL	102.866
4	1,10-*bis*[Bim][PF_6] (**2d**)	100 μg/mL	94.005
5	1% DMSO	100 μg/mL	97.587

5. Density Function Theory Calculations

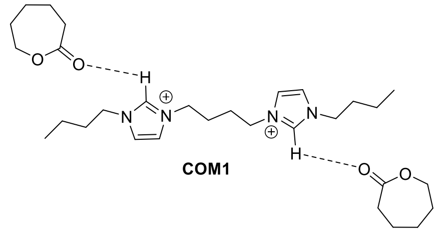

COM1

Atom	Bond	Angle	Dihedral	X	Y	Z
C				4.248335	-0.35857	-1.28414
H	1.084274			4.151304	0.147637	-0.3302
C	2.211196	163.3236		4.979674	-1.61325	-2.95157
H	1.07851	157.1829	3.553108	5.625881	-2.30908	-3.46286
C	1.362724	72.11482	-174.627	3.88063	-0.93611	-3.38815
H	1.078248	130.4546	178.2643	3.40249	-0.92728	-4.35454
N	1.341795	72.30075	0.205496	3.433724	-0.16011	-2.33173
N	1.338952	108.6683	-0.22341	5.194427	-1.23771	-1.63742
C	1.480529	125.6157	-179.215	6.277936	-1.7371	-0.76073
H	1.09356	106.9926	-131.814	7.21045	-1.65327	-1.32577
H	1.092645	106.6607	-16.943	6.341092	-1.0465	0.083634
C	1.530948	112.8554	105.4572	6.043642	-3.1733	-0.28507
H	1.097386	109.5224	58.33965	5.951931	-3.83707	-1.15412
H	1.097465	109.1227	-58.2981	5.089244	-3.22213	0.254526
C	1.536126	111.5914	179.9199	7.184621	-3.66232	0.619748
H	1.097379	109.283	-57.8658	8.134377	-3.59398	0.074283
H	1.097672	109.2815	58.08896	7.275867	-2.9906	1.483085
C	1.531342	112.526	-179.846	6.973625	-5.09929	1.105165
H	1.094971	111.3358	60.28027	6.916504	-5.79924	0.26507
H	1.092887	110.7685	-179.992	7.800257	-5.41737	1.745402
H	1.095282	111.3591	-60.2088	6.049171	-5.19276	1.68507
C	1.481649	125.1727	-176.35	2.306915	0.80133	-2.36657
H	1.093283	106.3179	3.352406	2.327653	1.333613	-1.41184
H	1.093376	106.7632	118.8625	2.52695	1.516287	-3.164
C	1.533212	113.5926	-119.942	0.945812	0.142594	-2.61995
H	1.096322	106.112	-169.173	0.243625	0.961142	-2.81702
H	1.097597	109.9112	-55.0963	0.983441	-0.45014	-3.54297
C	1.536819	114.9811	69.97402	0.412984	-0.71324	-1.46002
H	1.095875	109.7933	57.31744	0.34109	-0.10407	-0.5519
H	1.097073	110.3187	-60.4086	1.107278	-1.53425	-1.24212
C	1.535035	110.2115	178.122	-0.97004	-1.27957	-1.81053
H	1.092777	110.9235	-57.8434	-1.67464	-0.47586	-2.03801
H	1.094518	110.9141	62.00884	-0.91372	-1.9345	-2.68566

C	2.490465	121.8407	-148.747	-2.69653	-1.70396	-0.06653
C	2.214539	65.69341	-79.7287	-1.14297	-3.27535	-0.21266
H	1.091429	94.75764	101.8762	-3.27277	-0.80408	-0.28872
H	1.077939	157.5208	-1.47829	-0.26729	-3.77824	-0.58982
C	1.362892	71.95102	-178.952	-2.02743	-3.64478	0.756225
H	1.078345	130.6571	179.2316	-2.05884	-4.5232	1.3809
N	1.341467	29.34919	-78.2967	-1.57662	-2.05903	-0.71402
N	1.337079	137.8965	-81.3717	-2.98936	-2.65225	0.829455
C	1.480813	124.9144	-178.485	-4.15419	-2.62411	1.743336
H	1.093891	107.125	127.59	-4.68278	-3.57394	1.62077
H	1.09286	106.1903	12.5272	-4.80782	-1.82743	1.379452
C	1.53084	112.8027	-109.672	-3.75701	-2.39485	3.203869
H	1.097482	109.6143	-58.1415	-3.06552	-3.18392	3.525832
H	1.097014	109.1986	58.558	-3.21457	-1.44482	3.285337
C	1.535996	111.5442	-179.623	-4.98483	-2.38016	4.126658
H	1.097468	109.3091	57.77103	-5.52714	-3.32876	4.024216
H	1.097896	109.2363	-58.1759	-5.67551	-1.59289	3.797229
C	1.531365	112.5474	179.8255	-4.61299	-2.15642	5.595247
H	1.093	110.8301	179.7431	-5.50616	-2.1465	6.225165
H	1.094956	111.3157	-60.5145	-3.9556	-2.95027	5.964784
H	1.09516	111.3363	59.88045	-4.09668	-1.20068	5.734407
C	6.071672	91.8001	124.1954	-6.73413	1.80652	-2.93698
C	1.533108	108.377	175.3905	-8.06903	1.719559	-2.18803
C	1.533993	114.8715	-3.6895	-8.08345	0.741151	-1.00666
C	1.547611	52.01126	-77.6531	-5.53863	2.250801	-2.06035
C	1.524199	114.6913	59.27113	-7.06189	1.043877	0.083277
H	1.097249	63.65333	71.11003	-6.49804	0.846544	-3.41306
H	1.098366	108.8223	118.5107	-8.33568	2.720122	-1.82173
H	1.09744	111.0565	-64.1065	-7.93193	-0.29093	-1.34758
H	1.095401	112.0596	44.04019	-7.0268	2.110691	0.329408
H	1.097762	109.739	-139.014	-5.83809	3.094424	-1.42497
H	1.094384	142.5939	-11.107	-6.83185	2.533872	-3.74882
H	1.094912	108.939	-126.206	-8.85526	1.435098	-2.89496
H	1.096337	108.925	179.1905	-9.07314	0.766892	-0.53571
H	1.091098	110.4481	102.804	-4.71271	2.591751	-2.68654
H	1.09098	110.8575	164.7179	-7.29489	0.495492	0.997181
C	1.512185	113.9421	-15.807	-4.96607	1.147801	-1.19879
O	1.343657	120.3007	-63.8595	-5.71263	0.584366	-0.23411
O	1.224735	122.4433	114.9473	-3.83077	0.71531	-1.35376
C	5.754353	124.8634	-145.163	5.052201	4.080888	2.287642
C	1.533189	133.385	144.3368	4.493138	5.507669	2.336759

C	1.534048	114.8539	5.639889	3.410276	5.81213	1.293679
C	1.547758	55.67368	-123.399	3.990581	2.974071	2.496145
C	1.524532	114.7398	59.50207	2.172208	4.926954	1.382273
H	1.097497	53.71432	60.00918	5.575806	3.907586	1.338799
H	1.09838	108.784	127.8617	4.082215	5.692227	3.338517
H	1.09727	111.0584	-63.8203	3.813061	5.742582	0.275382
H	1.095737	112.0012	44.03734	1.835174	4.794878	2.416489
H	1.097534	109.6286	-171.436	3.329734	3.249099	3.328143
H	1.094497	117.609	-30.3991	5.802841	3.968041	3.07614
H	1.094933	108.9165	-116.897	5.318054	6.216656	2.211343
H	1.096257	108.9176	179.4904	3.075237	6.848118	1.42118
H	1.091281	110.4718	70.52289	4.470138	2.030267	2.761009
H	1.090796	110.8392	164.816	1.347894	5.354266	0.809781
C	1.513285	113.7802	-48.3253	3.161408	2.683964	1.263935
O	1.336472	120.3372	-64.9769	2.344872	3.61683	0.764748
O	1.226708	122.3999	113.9545	3.216585	1.610814	0.672229

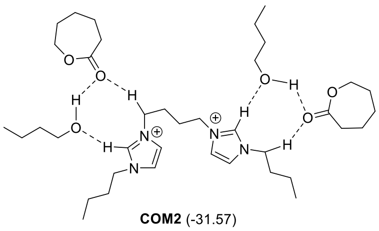

COM2 (-31.57)

Atom	Bond	Angle	Dihedral	X	Y	Z
C				-2.36446	-1.77124	-1.67612
H	1.093746			-2.53443	-1.12549	-0.80986
C	2.211842	160.9567		-2.68198	-2.81519	-3.60007
H	1.078409	157.0313	-0.29536	-3.2009	-3.09945	-4.50167
C	1.36239	72.3097	-178.992	-1.47449	-3.21092	-3.10869
H	1.077781	130.5435	178.4922	-0.75504	-3.91215	-3.49892
N	1.343563	72.10953	0.217364	-1.29063	-2.54468	-1.90817
N	1.3397	108.3747	-0.21278	-3.22182	-1.92247	-2.69438
C	1.485625	125.4697	179.8	-4.53714	-1.24368	-2.82192
H	1.092675	105.8014	-169.211	-5.04689	-1.73646	-3.65334
H	1.092582	107.6943	-53.5967	-5.10262	-1.44095	-1.9081
C	1.530452	112.7466	68.83139	-4.40518	0.262002	-3.06229
H	1.097618	109.6504	55.54418	-3.77903	0.44126	-3.94579
H	1.096104	108.4892	-62.1061	-3.90339	0.709544	-2.19664
C	1.535547	111.1033	177.4665	-5.78234	0.914597	-3.25062
H	1.097886	109.5366	-58.4777	-6.29788	0.445723	-4.09899

H	1.097029	108.7672	57.56235	-6.39386	0.710744	-2.36295
C	1.531286	112.5676	179.452	-5.69008	2.425717	-3.48049
H	1.095272	111.3393	60.37604	-5.10871	2.657365	-4.37936
H	1.093375	110.9712	-179.843	-6.68381	2.86347	-3.60838
H	1.095369	111.2757	-59.929	-5.21135	2.928169	-2.63303
C	1.478285	125.542	-175.572	-0.15439	-2.73764	-0.9824
H	1.092507	106.7324	-1.46573	-0.35305	-2.11545	-0.10663
H	1.093602	107.0195	113.5777	-0.17837	-3.78223	-0.65957
C	1.532924	113.8527	-124.585	1.210136	-2.40906	-1.59881
H	1.095471	107.1368	-165.583	1.973781	-2.8276	-0.93419
H	1.097699	109.2932	-51.1027	1.314358	-2.94076	-2.55347
C	1.536157	114.6896	73.69331	1.48646	-0.90927	-1.78334
H	1.096059	109.053	62.94194	1.500594	-0.42446	-0.80044
H	1.096844	111.2692	-56.3087	0.706056	-0.43246	-2.38889
C	1.535057	110.063	-178.17	2.855336	-0.70374	-2.44692
H	1.092622	110.2043	-55.4507	3.634877	-1.20281	-1.86635
H	1.092851	111.3449	64.22671	2.873275	-1.11753	-3.45825
C	2.501018	103.7958	-150.368	3.622916	1.504812	-1.55912
C	2.215285	65.26592	-114.46	3.231122	1.46769	-3.73917
H	1.092961	95.11258	64.95183	3.700256	1.171022	-0.52125
H	1.078284	157.2483	-1.97185	2.962018	1.045259	-4.69407
C	1.362626	72.04276	-179.719	3.643861	2.723321	-3.40783
H	1.078239	130.7436	179.2535	3.795447	3.598508	-4.01911
N	1.342718	28.98483	-114.124	3.224106	0.721302	-2.57399
N	1.338599	137.3619	-114.937	3.88395	2.724519	-2.04494
C	1.47996	125.4376	178.7843	4.317863	3.891874	-1.24539
H	1.093715	107.1689	130.5197	5.192433	4.31825	-1.74493
H	1.092529	106.6224	15.41953	4.644497	3.503898	-0.27771
C	1.531023	112.5651	-106.726	3.208775	4.933847	-1.07732
H	1.097431	109.4867	-59.0077	2.884035	5.285397	-2.0649
H	1.097163	109.0753	57.55992	2.33861	4.458696	-0.60742
C	1.535794	111.7322	179.3905	3.674574	6.128972	-0.23271
H	1.097568	109.3989	57.56954	4.556929	6.584181	-0.70057
H	1.097727	109.2668	-58.4642	3.997975	5.77143	0.753484
C	1.531331	112.4583	179.5596	2.580711	7.187101	-0.063
H	1.093031	110.806	179.9588	2.940162	8.023557	0.541858
H	1.095017	111.3174	-60.2883	2.262771	7.589685	-1.03042
H	1.095219	111.3121	60.10398	1.698132	6.771004	0.434416
C	6.280386	116.2754	-60.0019	-8.69999	-2.36736	1.7444
C	1.533056	141.4277	87.98893	-9.13476	-1.85545	3.12251
C	1.533815	114.8469	-22.5491	-8.06261	-1.06671	3.884732

C	1.547307	39.03108	148.0633	-8.26705	-1.25517	0.759647
C	1.524491	114.7294	59.8125	-7.56471	0.185509	3.17192
H	1.097305	72.27035	-12.8436	-7.88789	-3.09838	1.845273
H	1.098458	108.8132	99.67447	-10.0224	-1.21992	3.000483
H	1.09727	111.0545	-63.5161	-7.19724	-1.70032	4.116389
H	1.095672	112.0458	43.91916	-8.38638	0.765267	2.736903
H	1.097695	109.6898	171.8189	-8.98269	-0.42394	0.802693
H	1.094488	106.8344	-114.951	-9.53744	-2.90201	1.285333
H	1.095112	108.914	-145.078	-9.45247	-2.70696	3.733466
H	1.096354	108.9402	179.7353	-8.47517	-0.745	4.848212
H	1.091104	110.3908	53.67554	-8.26694	-1.63272	-0.26406
H	1.090617	110.9081	164.7954	-7.01968	0.833773	3.859051
C	1.511978	113.9775	-65.1748	-6.87098	-0.72815	1.003215
O	1.335383	120.6291	-64.4298	-6.57401	-0.08651	2.136068
O	1.228258	121.9995	114.6456	-5.96553	-0.8825	0.187766
C	3.640298	107.3422	170.146	-3.16323	1.170238	1.276395
H	1.100761	101.1093	60.35214	-2.95294	0.734858	2.265283
H	1.098165	74.02146	-44.4741	-4.0576	1.79844	1.383247
C	1.522837	145.07	-146.476	-1.9854	2.019612	0.817786
H	1.099835	108.8495	-88.5235	-1.10521	1.3712	0.697516
H	1.098029	108.5487	27.15887	-2.21409	2.426312	-0.17618
C	1.533818	113.0383	149.3367	-1.6566	3.162727	1.786174
H	1.097407	109.329	-57.3053	-2.54391	3.795268	1.916094
H	1.097721	109.5119	58.54731	-1.42951	2.749567	2.777497
C	1.53148	113.0009	-179.134	-0.48547	4.029106	1.313663
H	1.095993	111.1543	60.17712	-0.70198	4.492194	0.344192
H	1.094085	111.2707	-179.797	-0.27317	4.83212	2.025788
H	1.097199	111.0532	-59.84	0.427819	3.431125	1.20333
O	1.429541	42.33431	168.4605	-3.40704	0.136463	0.319604
H	0.982133	109.0284	-5.79245	-4.32218	-0.19991	0.43781
C	6.138738	113.9304	-43.366	7.288314	-4.29032	-0.17331
C	1.532977	139.6441	-76.5947	8.195552	-4.38752	1.058554
C	1.533585	114.8758	1.440759	7.970833	-3.29679	2.112917
C	1.547803	53.168	8.537067	5.7748	-4.37457	0.13953
C	1.523654	114.6791	59.8089	6.564739	-3.25807	2.698512
H	1.097393	56.99158	-159.668	7.492957	-3.36616	-0.72858
H	1.098397	108.8247	123.7197	8.051364	-5.36988	1.528287
H	1.097221	111.0919	-63.6018	8.20687	-2.30378	1.710279
H	1.095425	112.1386	44.13907	6.191408	-4.25864	2.942329
H	1.097852	109.6603	-179.388	5.590551	-5.18444	0.857478
H	1.09445	111.5253	104.513	7.526578	-5.11417	-0.85325

H	1.094977	108.8599	-121.065	9.240268	-4.35754	0.73199
H	1.096239	108.9583	179.6544	8.661159	-3.46146	2.948424
H	1.091248	110.3999	62.53001	5.211688	-4.60776	-0.76565
H	1.0904	111.036	165.2209	6.535246	-2.65455	3.606183
C	1.511854	114.023	-56.425	5.184012	-3.0891	0.672667
O	1.333325	120.584	-63.5277	5.592493	-2.58694	1.838314
O	1.230354	122.1128	115.4817	4.329763	-2.45582	0.053813
C	3.592101	104.0241	-174.377	3.414729	0.216014	2.389655
H	1.097973	44.19184	-160.383	4.394202	-0.10611	2.767028
H	1.100321	93.7441	88.53923	2.651715	-0.38616	2.905311
C	1.521868	150.927	-126.564	3.196402	1.692346	2.687746
H	1.099658	108.7367	36.58702	3.978271	2.274794	2.179137
H	1.098638	108.7893	152.5672	2.235974	2.002696	2.253849
C	1.534182	112.993	-85.1837	3.216349	2.010028	4.188544
H	1.097818	109.2541	-58.825	2.432577	1.429126	4.69199
H	1.097804	109.2554	56.85018	4.168769	1.672253	4.61747
C	1.531213	113.0623	178.9465	3.022421	3.498869	4.489139
H	1.095781	111.342	60.02686	2.062262	3.86145	4.105257
H	1.093503	111.19	-179.971	3.042262	3.690643	5.565511
H	1.095803	111.2608	-60.1551	3.814091	4.102972	4.031862
O	1.434555	76.0174	-21.0198	3.33912	-0.00865	0.97482
H	0.980559	108.3943	14.12056	3.724014	-0.8893	0.780357

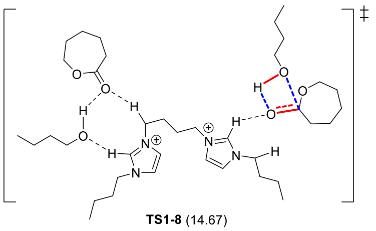

TS1-8 (14.67)

Atom	Bond	Angle	Dihedral	X	Y	Z
C				-2.75574	-1.61329	-2.2013
H	1.090819			-2.9209	-1.44185	-1.13678
C	2.21171	159.8474		-3.18943	-1.86996	-4.35484
H	1.078474	157.1512	6.663138	-3.78372	-1.87929	-5.25474
C	1.362575	72.21305	-172.016	-1.87747	-2.17874	-4.15476
H	1.077788	130.4192	178.3669	-1.126	-2.51901	-4.8484
N	1.342905	72.2599	0.261525	-1.62164	-2.00722	-2.80298
N	1.337787	108.5482	-0.1978	-3.71923	-1.52525	-3.12522
C	1.482628	124.8475	179.5268	-5.12163	-1.1295	-2.8517
H	1.09378	106.7236	-131.708	-5.75921	-1.86822	-3.34581

H	1.092779	105.9365	-16.4345	-5.25591	-1.2277	-1.77165
C	1.530916	112.6576	106.0321	-5.43982	0.287363	-3.33643
H	1.097771	109.8325	57.20813	-5.23262	0.368554	-4.4114
H	1.097503	109.12	-59.5379	-4.77407	0.99833	-2.83065
C	1.535506	111.4396	178.8137	-6.90536	0.657234	-3.06589
H	1.097436	109.2746	-58.2917	-7.5602	-0.06617	-3.56813
H	1.097778	109.2344	57.5033	-7.11011	0.56155	-1.99163
C	1.53134	112.6511	179.6758	-7.25207	2.073672	-3.53333
H	1.095022	111.3282	60.29986	-7.09438	2.187221	-4.61097
H	1.093096	110.8696	-179.954	-8.29984	2.306499	-3.32636
H	1.095414	111.3562	-60.0612	-6.63738	2.823585	-3.0237
C	1.478759	125.0122	-173.663	-0.36449	-2.34893	-2.1033
H	1.09243	106.6598	-6.86599	-0.51381	-2.09813	-1.05059
H	1.093877	107.1198	108.1525	-0.24278	-3.43389	-2.17129
C	1.530965	114.2889	-129.935	0.877636	-1.65065	-2.66311
H	1.09579	106.429	-170.514	1.741372	-2.11753	-2.17655
H	1.097627	109.5775	-56.1228	0.973136	-1.87253	-3.73383
C	1.535774	115.0588	69.08248	0.936768	-0.13529	-2.42063
H	1.096225	109.3304	58.60545	0.89969	0.061451	-1.34284
H	1.096724	111.2007	-60.5726	0.085639	0.374651	-2.8879
C	1.53474	109.6956	177.8007	2.250383	0.427241	-2.98045
H	1.092904	110.0198	-57.1224	3.101307	-0.08333	-2.52254
H	1.092778	111.2889	62.58196	2.313379	0.28602	-4.06224
C	2.50056	102.8631	-152.53	2.651621	2.436109	-1.54649
C	2.21545	65.26069	-117.532	2.364325	2.875766	-3.69879
H	1.094136	95.14967	61.53691	2.724948	1.875184	-0.60994
H	1.078317	157.2538	-1.52129	2.197534	2.665344	-4.74314
C	1.362598	72.05056	-179.181	2.588568	4.055798	-3.05543
H	1.078307	130.7309	179.226	2.647393	5.06364	-3.43429
N	1.342566	29.00043	-116.541	2.407325	1.876894	-2.74235
N	1.339039	137.3351	-118.812	2.767126	3.759394	-1.71561
C	1.479703	125.5721	-179.758	3.018704	4.74572	-0.64165
H	1.093705	107.0737	130.5222	3.845814	5.379104	-0.97467
H	1.092533	106.663	15.53088	3.36378	4.181101	0.227696
C	1.531079	112.7374	-106.789	1.780922	5.581037	-0.3035
H	1.097313	109.4991	-57.9239	1.433852	6.102755	-1.20431
H	1.097267	109.0908	58.61139	0.969509	4.910799	0.006957
C	1.535813	111.679	-179.484	2.072319	6.604399	0.803992
H	1.097482	109.3249	57.69991	2.896392	7.256733	0.488031
H	1.097884	109.3216	-58.2568	2.423071	6.078348	1.70154
C	1.531358	112.5375	179.6682	0.848701	7.456751	1.152293

H	1.092959	110.7859	-179.875	1.08811	8.175283	1.940299
H	1.094942	111.3168	-60.1402	0.497195	8.022415	0.283175
H	1.095272	111.3723	60.27018	0.018589	6.836924	1.507748
C	6.078073	112.6261	-47.9469	7.098053	-2.82104	-1.27993
C	1.533419	142.1658	-68.9931	7.951284	-3.17271	-0.05531
C	1.53422	114.8795	-0.49683	7.505005	-2.50964	1.254264
C	1.548249	52.12025	12.32775	5.603555	-3.20292	-1.14683
C	1.523713	114.7817	59.44727	6.082706	-2.84365	1.686933
H	1.097345	58.63953	-152.232	7.179495	-1.75106	-1.50944
H	1.098324	108.7996	121.7883	7.9455	-4.26279	0.078869
H	1.097285	111.0839	-63.9802	7.60271	-1.41819	1.197372
H	1.095326	112.1441	44.14082	5.85794	-3.91019	1.578698
H	1.097545	109.6585	177.6462	5.516442	-4.20958	-0.71828
H	1.094398	108.9798	109.92	7.494294	-3.3518	-2.15114
H	1.094904	108.8769	-122.999	8.991511	-2.8972	-0.25739
H	1.096224	108.9322	179.3295	8.170129	-2.83531	2.062504
H	1.091232	110.404	59.43431	5.127048	-3.22263	-2.12832
H	1.090393	110.9924	165.2448	5.915404	-2.56497	2.727753
C	1.511238	113.6799	-59.4317	4.800158	-2.22974	-0.31536
O	1.333328	120.5361	-64.5496	5.061479	-2.07081	0.982409
O	1.230719	122.2045	114.2031	3.902836	-1.53951	-0.79813
C	3.602559	104.8186	176.1079	2.84972	0.564174	2.051712
H	1.099989	68.92548	-117.445	3.902757	0.859526	2.169427
H	1.097859	64.99871	120.6037	2.720242	-0.39166	2.576031
C	1.522078	173.6429	158.4774	1.934981	1.617662	2.660101
H	1.100145	109.1564	143.8582	2.072907	2.568499	2.124187
H	1.098008	108.5947	-100.582	0.892787	1.315981	2.491456
C	1.53416	112.9634	21.80469	2.184434	1.833804	4.158334
H	1.097823	109.2139	-58.1539	2.045279	0.8827	4.688667
H	1.097811	109.4272	57.48974	3.23285	2.118332	4.316624
C	1.531162	113.0543	179.7358	1.269668	2.897827	4.771126
H	1.095944	111.2736	59.79811	0.213802	2.625337	4.661634
H	1.09342	111.237	179.7965	1.469831	3.025138	5.838503
H	1.095877	111.3338	-60.2706	1.412776	3.871837	4.289701
O	1.43549	76.54085	0.894478	2.529871	0.403011	0.66162
H	0.981411	108.765	1.828526	3.074789	-0.32613	0.294755
C	6.429063	109.7735	-9.2311	-6.10351	-0.8477	3.495693
C	1.53115	117.2839	84.18009	-5.71209	0.449055	4.20958
C	1.532527	114.3839	-60.7012	-4.22565	0.806375	4.102529
C	1.536319	2.619723	44.02389	-5.80789	-0.88153	1.988463
C	1.525097	114.4684	62.39096	-3.7374	1.058313	2.679832

H	1.095307	108.4031	-41.768	-5.60765	-1.7065	3.960762
H	1.098947	109.3402	61.3462	-6.30322	1.281279	3.802576
H	1.097133	111.1134	-62.0616	-3.59945	0.036639	4.570588
H	1.096881	110.7441	44.08667	-4.45234	1.681057	2.128284
H	1.100247	110.8806	91.48366	-6.1875	0.025055	1.493943
H	1.095016	105.0094	-155.107	-7.17934	-1.00673	3.623566
H	1.095174	109.0328	176.9157	-5.98588	0.371206	5.267117
H	1.09719	108.9927	-178.599	-4.03951	1.731333	4.662558
H	1.091906	109.5555	-25.587	-6.33269	-1.72319	1.531888
H	1.091868	110.4563	163.5204	-2.77845	1.580256	2.692291
C	1.518417	117.7125	-145.154	-4.3615	-1.04455	1.556058
O	1.347766	120.7532	-62.3539	-3.43812	-0.11592	1.874617
O	1.345829	116.3694	153.7644	-4.15786	-1.60432	0.349228
C	2.75626	120.2668	36.93852	-2.65974	-2.67531	2.984896
H	1.096225	137.9087	28.93802	-2.72729	-3.57438	3.608457
H	1.096176	84.20274	-81.1786	-2.57261	-1.81122	3.653747
C	1.529405	101.8478	169.432	-1.44443	-2.74879	2.059307
H	1.099707	108.6052	-87.6703	-1.5928	-3.57637	1.350461
H	1.09752	108.6456	28.92063	-1.39131	-1.82089	1.475597
C	1.533863	112.9267	150.8784	-0.12777	-2.95927	2.817485
H	1.097912	109.4191	-56.6865	0.001727	-2.1556	3.554199
H	1.097028	109.3955	59.18927	-0.18295	-3.89268	3.391222
C	1.532245	112.7968	-178.704	1.091245	-3.0008	1.890092
H	1.096837	111.5134	61.59143	1.215444	-2.05628	1.346483
H	1.094496	111.2747	-178.438	2.012689	-3.17924	2.453139
H	1.09676	111.0538	-59.0247	0.998529	-3.80798	1.153387
O	1.433326	33.43021	54.94313	-3.90285	-2.56896	2.27937
H	1.224257	118.9032	77.03026	-3.89041	-2.56996	1.055177

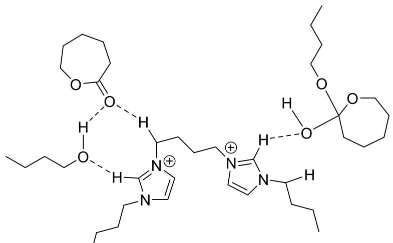

INT1-9 (-14.26)

Atom	Bond	Angle	Dihedral	X	Y	Z
C				-3.60759	-0.78196	-1.73307
H	1.085478			-3.55204	-0.65166	-0.65688
C	2.210648	161.1072		-4.39741	-0.81829	-3.79749
H	1.078519	157.1988	4.619172	-5.11175	-0.66834	-4.5915

C	1.363037	72.12069	-173.92	-3.14583	-1.35769	-3.81873
H	1.077963	130.4147	178.3438	-2.57829	-1.77466	-4.63484
N	1.341601	72.34357	0.211922	-2.66505	-1.32271	-2.5199
N	1.338074	108.6875	-0.09404	-4.66847	-0.47071	-2.48679
C	1.481656	125.0231	179.8569	-5.91831	0.145151	-1.98288
H	1.093603	106.8282	-132.906	-6.74732	-0.42576	-2.41041
H	1.092518	106.2383	-17.9724	-5.92367	-0.015	-0.90218
C	1.530882	112.7201	104.6732	-6.0294	1.630725	-2.33548
H	1.097549	109.5813	57.35039	-5.96475	1.756276	-3.42391
H	1.097599	109.1742	-59.2185	-5.17537	2.168489	-1.90402
C	1.535961	111.6274	178.9629	-7.34458	2.237174	-1.82391
H	1.097427	109.2637	-58.0814	-8.18954	1.684673	-2.25416
H	1.097729	109.3157	57.79765	-7.40928	2.099426	-0.73678
C	1.531309	112.5393	179.9405	-7.47677	3.724298	-2.16434
H	1.09498	111.3165	60.2367	-7.45409	3.888411	-3.24672
H	1.09298	110.8187	179.978	-8.42099	4.126427	-1.78838
H	1.095321	111.3617	-60.184	-6.66513	4.309323	-1.71858
C	1.48125	124.9214	-173.775	-1.39623	-1.91602	-2.03805
H	1.093042	106.2937	-2.30958	-1.38596	-1.77899	-0.95368
H	1.093858	106.6829	112.4822	-1.45577	-2.98978	-2.23809
C	1.531602	114.1483	-126.26	-0.14253	-1.3376	-2.70097
H	1.09573	106.364	-168.987	0.689085	-1.98895	-2.40979
H	1.097854	109.6796	-54.5795	-0.22908	-1.42166	-3.79217
C	1.53623	115.3116	70.70236	0.205258	0.104657	-2.30229
H	1.095918	109.3508	57.82088	0.336071	0.159517	-1.21559
H	1.096808	111.3174	-61.2416	-0.59672	0.79948	-2.57986
C	1.535225	109.7192	176.9526	1.517633	0.526702	-2.97792
H	1.092703	109.9048	-55.7861	2.311016	-0.1804	-2.72385
H	1.09287	111.2759	63.74141	1.416264	0.542658	-4.06596
C	2.501004	104.7488	-150.305	2.540099	2.180903	-1.40527
C	2.215308	65.24677	-114.394	1.92696	3.018177	-3.36247
H	1.093322	95.53826	64.51051	2.69092	1.470355	-0.58813
H	1.078337	157.2596	-1.2785	1.525697	3.015993	-4.36337
C	1.362624	72.04407	-178.99	2.481973	4.024517	-2.63036
H	1.078174	130.6824	179.1418	2.649313	5.061322	-2.87427
N	1.342925	28.97085	-113.202	1.971913	1.875591	-2.58315
N	1.338759	137.3294	-115.864	2.858114	3.48131	-1.41447
C	1.480786	125.3785	-177.993	3.517031	4.210625	-0.30692
H	1.094033	107.1195	123.1892	4.429532	4.656978	-0.71315
H	1.092329	106.4597	8.118524	3.812736	3.457472	0.426903
C	1.530845	113.0332	-114.049	2.617725	5.27397	0.328711

H	1.097289	109.7118	-58.7654	2.309876	6.001607	-0.43275
H	1.097039	109.2368	58.02914	1.702443	4.797553	0.701242
C	1.535989	111.3429	179.8409	3.337038	6.002738	1.47359
H	1.097523	109.3022	57.40207	4.261517	6.454589	1.091848
H	1.097773	109.2508	-58.5184	3.64258	5.271157	2.232891
C	1.531463	112.5886	179.3551	2.467517	7.084938	2.120239
H	1.093009	110.8147	179.9514	3.006226	7.58348	2.930126
H	1.094929	111.3271	-60.2972	2.178875	7.851019	1.393138
H	1.095368	111.4076	60.09385	1.550035	6.661133	2.54267
C	5.974278	113.776	-44.2019	5.79675	-3.63552	-2.739
C	1.533254	143.6026	-71.8495	6.784665	-4.33738	-1.7997
C	1.533928	114.872	0.540532	6.727851	-3.8721	-0.33915
C	1.548318	53.00536	9.432251	4.309356	-3.80469	-2.34361
C	1.523432	114.7107	59.437	5.375824	-4.06054	0.337134
H	1.097382	58.16781	-153.292	6.032725	-2.56647	-2.81447
H	1.098354	108.7925	122.8202	6.598785	-5.41932	-1.83508
H	1.097247	111.1102	-63.9766	7.016868	-2.81746	-0.24884
H	1.095195	112.2019	44.10046	4.936933	-5.03996	0.119043
H	1.097694	109.656	175.9666	4.117271	-4.84954	-2.06735
H	1.094468	107.5685	108.1996	5.914775	-4.0462	-3.7466
H	1.094876	108.8692	-121.968	7.800565	-4.19112	-2.18089
H	1.096217	108.9608	179.2977	7.459984	-4.4418	0.2449
H	1.091254	110.3942	57.87111	3.66171	-3.56698	-3.18912
H	1.090305	111.0634	165.2845	5.46165	-3.96014	1.419409
C	1.511717	113.8532	-61.091	3.869413	-2.89806	-1.21677
O	1.332615	120.4624	-63.8192	4.404315	-3.02127	-0.00245
O	1.230972	122.1181	114.9974	3.030508	-2.01198	-1.37921
C	3.589566	103.9167	175.9238	2.933809	-0.33185	1.8656
H	1.096053	31.48804	-170.481	3.678783	-1.13393	1.810792
H	1.098896	112.4853	102.6116	2.12977	-0.67355	2.532184
C	1.523936	130.1199	-111.264	3.56689	0.936565	2.424812
H	1.099687	108.7467	32.0027	4.381748	1.24939	1.755889
H	1.099455	108.7814	148.2559	2.816534	1.740116	2.416303
C	1.53466	113.1057	-89.9457	4.110321	0.760901	3.849244
H	1.097691	109.2371	-57.663	3.296276	0.43416	4.509165
H	1.097753	109.2613	58.02257	4.852069	-0.04831	3.856614
C	1.531281	113.0307	-179.851	4.742796	2.037399	4.410804
H	1.096104	111.2706	60.11269	4.015607	2.8565	4.452193
H	1.093385	111.2116	179.9299	5.118316	1.879523	5.425472
H	1.095747	111.3254	-60.1927	5.586282	2.368519	3.794725
O	1.440173	78.79983	-3.62747	2.389545	-0.09178	0.554019

H	0.979989	108.2256	9.140665	2.610341	-0.85883	-0.01455
C	5.201098	97.81787	36.38686	-3.22831	-4.04518	2.339347
C	1.537824	164.3599	-44.7293	-3.95879	-4.30282	3.667848
C	1.534457	115.2381	-43.2285	-3.91429	-3.14526	4.674151
C	1.53756	52.34298	-17.9259	-3.87769	-2.94152	1.488274
C	1.524872	114.9958	52.39484	-4.36303	-1.7957	4.124103
H	1.096782	72.84075	-150.672	-2.17451	-3.79787	2.516216
H	1.098628	108.4017	79.4631	-5.00629	-4.54851	3.44568
H	1.096894	110.293	-69.4286	-2.89753	-3.02118	5.066543
H	1.096108	112.551	51.17717	-5.33596	-1.84886	3.622056
H	1.094556	110.3367	148.4744	-4.96865	-3.00937	1.545479
H	1.095402	84.39848	100.219	-3.23492	-4.97355	1.757978
H	1.09551	109.0409	-165.604	-3.53416	-5.19436	4.142157
H	1.096931	109.2414	173.8892	-4.55148	-3.38844	5.533284
H	1.095195	110.4647	31.10215	-3.61492	-3.06755	0.432567
H	1.092637	110.4499	171.7199	-4.44265	-1.06231	4.93012
C	1.532739	114.4366	-88.9237	-3.45997	-1.51731	1.870916
O	1.385147	116.6407	-41.6126	-3.37298	-1.23817	3.224852
O	1.43694	106.5845	-164.118	-4.41379	-0.63233	1.261143
C	2.474069	132.6759	59.43245	-1.2662	-0.37589	1.945289
H	1.095164	133.2868	26.05827	-0.32191	-0.52842	1.411959
H	1.092634	90.80811	-90.2575	-1.12508	-0.68143	2.984797
C	1.529635	101.9965	158.7432	-1.67175	1.09731	1.874548
H	1.099248	109.4802	-78.1674	-1.9214	1.359367	0.836594
H	1.097072	109.6343	39.3492	-2.57251	1.261124	2.479
C	1.53509	111.9528	160.016	-0.56058	2.023718	2.387945
H	1.097417	109.1523	-56.7165	-0.2984	1.737059	3.414303
H	1.098404	108.9241	58.80015	0.343564	1.863602	1.785138
C	1.531622	113.0311	-178.617	-0.95361	3.503782	2.359568
H	1.094928	111.2789	60.1292	-1.83213	3.69368	2.984872
H	1.094591	111.3059	179.6973	-0.1443	4.137479	2.735823
H	1.096476	111.4703	-59.8024	-1.19585	3.835275	1.342861
O	1.420603	107.2205	79.2423	-2.181	-1.28577	1.297556
H	0.969137	105.9244	161.3765	-4.33476	0.210136	1.733619

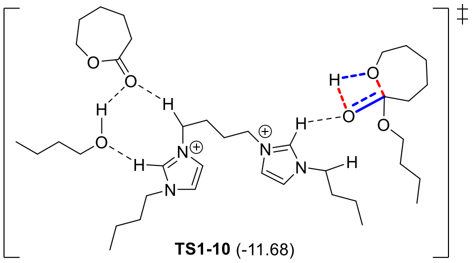

Atom	Bond	Angle	Dihedral	X	Y	Z
C				-2.80334	-0.23268	-2.43386
H	1.093294			-2.92836	-0.46422	-1.37271
C	2.212884	159.7778		-3.29359	0.440198	-4.48417
H	1.078386	157.2083	5.090669	-3.89113	0.86546	-5.27474
C	1.362654	72.19649	-173.48	-2.04181	-0.09756	-4.51029
H	1.077972	130.4471	178.4126	-1.35714	-0.23599	-5.33132
N	1.342808	72.21715	0.295973	-1.74891	-0.50717	-3.2187
N	1.338005	108.4785	-0.38584	-3.75041	0.344309	-3.18247
C	1.482208	124.6668	-179.449	-5.06354	0.805481	-2.67264
H	1.093995	107.029	-126.715	-5.83111	0.344923	-3.30157
H	1.092778	105.8738	-11.138	-5.15227	0.397194	-1.66289
C	1.530929	112.5312	110.8597	-5.1901	2.33117	-2.67319
H	1.097658	109.8334	57.95006	-5.03904	2.717032	-3.68963
H	1.097321	109.0708	-58.8064	-4.39362	2.757231	-2.05014
C	1.535786	111.5639	179.5243	-6.56208	2.78395	-2.15231
H	1.097502	109.2934	-58.1187	-7.35032	2.341514	-2.77476
H	1.097663	109.2296	57.79347	-6.71214	2.389712	-1.13894
C	1.53136	112.5833	179.8807	-6.71286	4.30782	-2.14019
H	1.095012	111.3242	60.22964	-6.60419	4.726731	-3.14605
H	1.093046	110.8563	179.9863	-7.6977	4.598454	-1.7655
H	1.095328	111.3427	-60.1528	-5.96044	4.777758	-1.49772
C	1.477979	125.108	-175.963	-0.53072	-1.22347	-2.78586
H	1.092295	106.5564	-2.68817	-0.65145	-1.42662	-1.71944
H	1.093728	107.2799	112.3965	-0.51657	-2.18481	-3.30727
C	1.531492	114.1514	-125.465	0.771588	-0.46332	-3.0535
H	1.09592	106.4717	-172.598	1.588843	-1.15291	-2.8135
H	1.097585	109.5329	-58.0841	0.856558	-0.24723	-4.12624
C	1.536133	114.9067	66.88234	0.950972	0.822069	-2.23173
H	1.096437	109.4716	58.17275	0.910836	0.581102	-1.16285
H	1.096803	111.0638	-60.8845	0.151156	1.541481	-2.44554
C	1.534847	109.9753	177.594	2.312779	1.457812	-2.54328
H	1.092757	110.0759	-58.7855	3.116634	0.754251	-2.3132
H	1.092787	111.2603	60.99926	2.391262	1.728985	-3.59898
C	2.500611	102.725	-154.237	2.807558	2.748876	-0.45968

C	2.215773	65.2496	-117.053	2.588972	3.983861	-2.28634
H	1.094357	95.16106	62.18693	2.831849	1.872721	0.195591
H	1.078307	157.2453	-1.81977	2.43199	4.192798	-3.3325
C	1.362631	72.03831	-179.459	2.865871	4.818513	-1.24545
H	1.078335	130.7518	179.2211	2.988881	5.889386	-1.21537
N	1.342662	28.98954	-116.384	2.556395	2.697413	-1.77764
N	1.338999	137.3217	-117.929	3.000122	4.02892	-0.11715
C	1.480047	125.5768	179.534	3.277969	4.521909	1.250438
H	1.093624	107.0145	132.6895	4.122498	5.212752	1.176147
H	1.092491	106.6891	17.69025	3.606509	3.658952	1.834307
C	1.531197	112.7327	-104.62	2.06324	5.195619	1.894729
H	1.097379	109.5011	-57.7162	1.729118	6.02907	1.263887
H	1.097227	109.0655	58.84486	1.235038	4.477109	1.936356
C	1.535797	111.6482	-179.325	2.383594	5.712065	3.305165
H	1.097471	109.3221	57.7966	3.223684	6.416235	3.251919
H	1.097905	109.3073	-58.1711	2.722261	4.875194	3.929942
C	1.53141	112.5188	179.7694	1.183004	6.395143	3.966379
H	1.092973	110.7976	-179.86	1.4423	6.753432	4.965871
H	1.094963	111.2972	-60.1312	0.84461	7.257317	3.38235
H	1.095216	111.36	60.29179	0.338211	5.705672	4.068621
C	5.988972	113.9464	-48.5366	6.896387	-2.39137	-2.33797
C	1.533201	142.1429	-73.5431	7.76947	-3.18285	-1.35716
C	1.533963	114.9106	2.652541	7.381111	-3.03326	0.119266
C	1.548342	53.95677	10.33952	5.393486	-2.76124	-2.29504
C	1.523838	114.7706	59.63253	5.961918	-3.47579	0.454146
H	1.097382	56.78523	-154.275	7.008859	-1.31308	-2.168
H	1.098232	108.8033	124.9259	7.73079	-4.24653	-1.62775
H	1.097294	111.1326	-63.8975	7.512014	-1.99841	0.459866
H	1.095339	112.1609	43.9804	5.700423	-4.42426	-0.0273
H	1.097496	109.6547	177.8252	5.282783	-3.85256	-2.25931
H	1.094381	108.9814	108.3567	7.249496	-2.57906	-3.35667
H	1.09493	108.8689	-119.874	8.812616	-2.87521	-1.48394
H	1.096238	108.9084	179.4401	8.057969	-3.64378	0.728251
H	1.091214	110.3882	59.62947	4.889763	-2.41057	-3.19728
H	1.09032	111.0214	165.1229	5.832722	-3.59386	1.530326
C	1.511351	113.7301	-59.2553	4.647396	-2.14123	-1.13611
O	1.334029	120.5099	-64.9113	4.945234	-2.47907	0.11959
O	1.23053	122.2214	113.9215	3.765299	-1.29887	-1.29901
C	3.586223	105.0175	174.6899	2.938699	-0.32088	2.163366
H	1.09982	65.73669	-117.426	4.022605	-0.18598	2.292036
H	1.097875	67.36835	119.93	2.721553	-1.38387	2.331371

C	1.521826	173.2429	-173.084	2.176773	0.527889	3.170848
H	1.100264	109.1531	115.7277	2.398117	1.590142	2.98862
H	1.097719	108.7322	-128.628	1.100489	0.401705	2.995696
C	1.53446	112.9227	-6.20928	2.517102	0.178149	4.625642
H	1.09773	109.1751	-58.4264	2.291239	-0.88116	4.804116
H	1.097906	109.4038	57.22521	3.597821	0.289058	4.78421
C	1.53117	113.0382	179.5107	1.760808	1.038716	5.641479
H	1.095509	111.3137	59.88524	0.676908	0.921316	5.534156
H	1.093411	111.1957	179.9192	2.024307	0.763791	6.666434
H	1.096072	111.3133	-60.2238	1.994129	2.102147	5.514788
O	1.436417	77.3197	1.903088	2.546872	0.051233	0.832464
H	0.981053	108.5289	2.970267	3.016753	-0.52846	0.195571
C	6.100708	99.95194	-35.0329	-6.13002	-3.48739	1.528853
C	1.536118	113.7276	144.0575	-6.8448	-2.96119	2.782591
C	1.536454	116.4649	-72.6114	-6.0073	-2.09389	3.734998
C	1.53807	9.691342	44.4413	-5.62039	-2.40053	0.567253
C	1.524678	115.2195	65.0367	-5.53958	-0.762	3.158862
H	1.095053	117.2918	13.07569	-5.29225	-4.13424	1.809662
H	1.09902	109.012	50.63494	-7.73132	-2.38876	2.475579
H	1.097033	110.9826	-58.6905	-5.12926	-2.64312	4.096768
H	1.098348	111.2217	42.12087	-6.3384	-0.2754	2.583116
H	1.094855	111.5088	43.57947	-6.34768	-1.58972	0.456159
H	1.095119	99.96665	-101.206	-6.83544	-4.11948	0.979175
H	1.095607	108.1725	165.2727	-7.22435	-3.82096	3.345713
H	1.096523	108.3532	-175.088	-6.61473	-1.8666	4.619155
H	1.097121	109.7362	-74.0892	-5.4773	-2.82822	-0.43289
H	1.094208	110.6298	162.2308	-5.23539	-0.08113	3.959595
C	1.517052	116.2025	167.8269	-4.27825	-1.7807	0.907722
O	1.447412	112.1303	-81.4088	-4.35796	-0.90874	2.335915
O	1.353616	117.9388	-167.115	-3.91726	-0.65138	0.25459
C	2.409997	141.5803	51.9448	-2.00261	-2.37537	1.432976
H	1.095223	105.2501	-97.475	-1.94203	-2.3455	2.526114
H	1.094381	83.24586	155.453	-1.7887	-1.37222	1.051393
C	1.521982	132.4915	42.20748	-1.05045	-3.41105	0.852294
H	1.096246	108.9881	-75.4566	-1.34138	-4.40469	1.212581
H	1.099088	108.8449	39.78659	-1.17497	-3.43512	-0.23945
C	1.534149	112.6283	162.2084	0.414986	-3.13201	1.210406
H	1.099429	110.0097	-56.6826	0.699336	-2.1248	0.873634
H	1.097878	109.0922	59.14626	0.522922	-3.12667	2.302952
C	1.531984	112.8123	-179.188	1.379101	-4.16095	0.611451
H	1.096506	111.2582	58.89883	1.303336	-4.18729	-0.48212

H	1.09441	111.1235	178.8932	2.416664	-3.92496	0.867398
H	1.094647	111.3136	-60.8569	1.163539	-5.16926	0.978979
O	1.353893	110.0309	53.16851	-3.34779	-2.75045	1.071574
H	1.191708	123.447	150.6029	-4.10436	-0.19329	1.417234

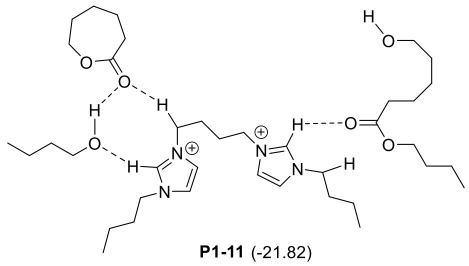

P1-11 (-21.82)

Atom	Bond	Angle	Dihedral	X	Y	Z
C				-2.46693	-0.51074	-2.70113
H	1.08649			-2.61958	-0.73003	-1.64801
C	2.210324	160.4066		-2.89179	0.083071	-4.78738
H	1.078388	157.1994	5.438484	-3.47356	0.451414	-5.61731
C	1.362815	72.21179	-173.262	-1.60769	-0.3706	-4.7369
H	1.077947	130.466	178.4716	-0.87316	-0.48001	-5.51823
N	1.342571	72.29701	0.279387	-1.35668	-0.73218	-3.42279
N	1.33745	108.6373	-0.32747	-3.41026	-0.01507	-3.50935
C	1.48335	124.9495	179.9237	-4.78199	0.358017	-3.08567
H	1.094011	106.826	-126.246	-5.47312	-0.17457	-3.74563
H	1.092611	105.9022	-10.7876	-4.89959	-0.03439	-2.07276
C	1.530737	112.5688	111.5256	-5.02271	1.868782	-3.13856
H	1.097664	109.8785	57.66092	-4.83897	2.24065	-4.15484
H	1.097308	109.1676	-59.1938	-4.30234	2.371629	-2.48107
C	1.535967	111.4452	179.1584	-6.45573	2.224259	-2.71514
H	1.097489	109.2789	-57.8171	-7.16661	1.702003	-3.36812
H	1.097539	109.2206	58.06558	-6.63646	1.847869	-1.70012
C	1.531369	112.5796	-179.771	-6.72673	3.730666	-2.76402
H	1.094997	111.3041	60.18463	-6.58897	4.127053	-3.77542
H	1.093053	110.855	179.9308	-7.75289	3.951537	-2.4591
H	1.095363	111.3732	-60.1783	-6.05517	4.279012	-2.09459
C	1.478189	125	-174.985	-0.12748	-1.37487	-2.91184
H	1.092331	106.5499	-4.6045	-0.28486	-1.54255	-1.84399
H	1.093828	107.2103	110.4818	-0.04952	-2.35355	-3.3941
C	1.530822	114.3111	-127.344	1.151782	-0.56943	-3.153
H	1.095938	106.2159	-172.465	1.982796	-1.21768	-2.85255
H	1.09751	109.658	-58.0572	1.276904	-0.38839	-4.22822

C	1.536094	114.9731	67.21657	1.245557	0.748706	-2.36987
H	1.096362	109.4006	56.47199	1.141121	0.543668	-1.29792
H	1.096709	111.1217	-62.6109	0.444964	1.439309	-2.66122
C	1.534706	109.7909	175.7805	2.611231	1.403867	-2.61689
H	1.092873	110.0211	-57.7853	3.41242	0.720531	-2.32445
H	1.092759	111.2936	62.02319	2.746338	1.653982	-3.67203
C	2.500523	102.575	-153.23	2.95495	2.747221	-0.53606
C	2.215551	65.26056	-117.986	2.85526	3.937379	-2.40214
H	1.093528	95.26244	60.79438	2.930763	1.88969	0.142073
H	1.078312	157.2442	-1.62968	2.767808	4.120699	-3.46115
C	1.362667	72.04048	-179.161	3.062257	4.797544	-1.36573
H	1.078357	130.7634	179.2443	3.181444	5.869221	-1.35323
N	1.342607	29.00055	-116.935	2.790694	2.663441	-1.86594
N	1.339023	137.3332	-119.311	3.12359	4.035567	-0.21247
C	1.479879	125.5865	-179.945	3.31284	4.561718	1.157708
H	1.093569	106.9666	133.9063	4.145488	5.269403	1.115618
H	1.092581	106.6955	18.97962	3.627159	3.718375	1.777151
C	1.531172	112.7888	-103.424	2.05042	5.221627	1.719228
H	1.097373	109.4937	-57.4848	1.732949	6.032348	1.051267
H	1.097242	109.0856	59.08223	1.236941	4.485435	1.73386
C	1.535787	111.6291	-179.09	2.282768	5.777553	3.131886
H	1.097445	109.3188	58.04254	3.106	6.502807	3.10578
H	1.097945	109.3109	-57.9114	2.609912	4.964204	3.792888
C	1.531347	112.541	-179.97	1.032091	6.442141	3.714255
H	1.092976	110.7936	-179.851	1.229685	6.829268	4.717094
H	1.094991	111.3251	-60.1189	0.701807	7.281787	3.093842
H	1.095242	111.358	60.30563	0.20136	5.732324	3.789093
C	6.126348	112.6847	-52.9538	7.288941	-2.39232	-1.50328
C	1.533204	142.7167	-50.8385	7.859173	-3.34934	-0.44987
C	1.534	114.8947	-6.21827	7.109198	-3.35781	0.888268
C	1.548528	48.77191	24.94622	5.816469	-2.67357	-1.89143
C	1.5235	114.7111	59.7697	5.641227	-3.75412	0.793128
H	1.097308	62.17521	-138.505	7.382351	-1.35199	-1.16701
H	1.098307	108.8211	116.0517	7.861942	-4.36782	-0.86094
H	1.097291	111.138	-63.7289	7.174282	-2.38133	1.384544
H	1.095315	112.1134	43.92269	5.489304	-4.61939	0.138953
H	1.097594	109.6795	175.8287	5.67138	-3.75214	-2.03412
H	1.094376	108.1798	122.3367	7.887463	-2.47356	-2.41587
H	1.0949	108.8562	-128.738	8.907943	-3.09217	-0.26891
H	1.096238	108.9292	179.5751	7.591173	-4.07626	1.561519
H	1.091219	110.3797	57.6016	5.571679	-2.1825	-2.83466

H	1.090318	111.0561	165.0745	5.236156	-3.9956	1.776184
C	1.511316	113.6575	-61.2108	4.814699	-2.15826	-0.88397
O	1.334563	120.5566	-64.8874	4.770122	-2.66623	0.349333
O	1.23034	122.2544	113.9073	4.027196	-1.25016	-1.14652
C	3.628686	103.5147	166.2731	2.724184	-0.28779	2.172652
H	1.099716	66.93399	-106.464	3.791464	-0.2244	2.430084
H	1.098256	66.95973	131.4593	2.413397	-1.32969	2.327608
C	1.521828	174.0599	-169.568	1.909791	0.634105	3.068668
H	1.100091	109.0775	122.5997	2.236604	1.672218	2.908313
H	1.097955	108.7004	-121.784	0.857925	0.588756	2.757176
C	1.534283	113.0377	0.62781	2.031164	0.285144	4.557802
H	1.097843	109.1945	-58.6347	1.69724	-0.74886	4.714599
H	1.097838	109.4406	57.04244	3.087911	0.311065	4.854216
C	1.531211	112.9935	179.2923	1.225103	1.22394	5.459763
H	1.095644	111.3218	59.56894	0.158519	1.197417	5.210501
H	1.093394	111.2065	179.626	1.326922	0.945358	6.512158
H	1.096015	111.2883	-60.5337	1.564399	2.261219	5.35887
O	1.436012	76.59749	12.73682	2.521576	0.08236	0.800037
H	0.980901	108.6338	-0.56046	3.054379	-0.5145	0.232547
C	6.141079	109.8874	-27.4481	-6.45344	-3.00193	1.775384
C	1.541049	116.969	125.6216	-6.92965	-2.23524	3.024482
C	1.546385	118.0979	-80.6908	-5.85737	-1.64533	3.969742
C	1.538058	7.261425	28.78253	-5.85202	-2.18134	0.621891
C	1.522506	115.5197	99.27541	-5.56993	-0.161	3.790403
H	1.094981	115.5394	-5.96658	-5.75117	-3.78898	2.069243
H	1.098651	109.1627	45.13333	-7.62362	-1.44085	2.717248
H	1.095536	110.4546	-25.5044	-4.92183	-2.20932	3.886776
H	1.100273	109.3603	55.94636	-6.50609	0.407258	3.896652
H	1.094189	111.2275	50.1447	-6.36978	-1.2246	0.504302
H	1.095891	100.3624	-119.275	-7.32895	-3.51868	1.366196
H	1.095621	106.0122	158.2488	-7.53458	-2.94347	3.601422
H	1.096553	108.815	-141.694	-6.19528	-1.76465	5.006084
H	1.09893	109.3145	-66.5594	-5.9973	-2.72247	-0.32347
H	1.100393	109.5834	173.5046	-4.88493	0.180203	4.581113
C	1.512095	119.132	173.4998	-4.37696	-1.84956	0.645456
O	1.433519	109.8975	-65.2602	-4.99103	0.071387	2.499725
O	1.229269	122.5642	-173.499	-3.85357	-1.10249	-0.17859
C	2.388397	146.2706	8.64543	-2.27391	-2.22259	1.714313
H	1.09252	128.2162	-38.3291	-2.07018	-2.34636	2.780509
H	1.092508	83.27262	-147.332	-2.0927	-1.18207	1.43493
C	1.524818	109.7344	101.6619	-1.43876	-3.19088	0.883655

H	1.096303	108.8288	-85.6115	-1.70952	-4.21713	1.158215
H	1.098289	109.055	30.0613	-1.70233	-3.07376	-0.17609
C	1.53486	112.3912	152.516	0.068783	-2.97996	1.08021
H	1.09973	110.1004	-58.7791	0.345681	-1.95197	0.804577
H	1.097914	109.0375	56.90708	0.308087	-3.07861	2.147176
C	1.532259	113.0112	178.3961	0.921453	-3.97155	0.281762
H	1.09708	111.4598	58.24147	0.708967	-3.90927	-0.79274
H	1.094391	111.1217	178.4156	1.989036	-3.77605	0.422247
H	1.09457	111.2551	-61.2947	0.724268	-5.00297	0.590611
O	1.333358	114.1357	10.52466	-3.68625	-2.50205	1.580882
H	0.965077	107.9566	170.1708	-4.96459	1.024847	2.352791

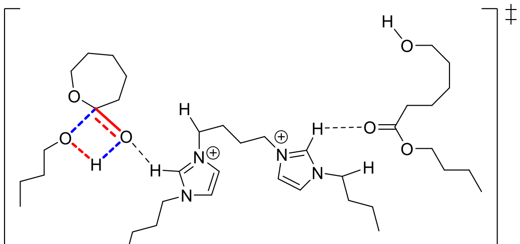

TS1-12 (18.02)

Atom	Bond	Angle	Dihedral	X	Y	Z
C				5.378052	-0.61158	-1.32214
H	1.094508			5.245696	0.097998	-0.49938
C	2.214379	162.5114		6.21884	-2.16773	-2.65441
H	1.078483	157.1388	0.829863	6.929656	-2.90101	-3.00106
C	1.36217	72.1731	-177.178	5.05024	-1.73485	-3.2044
H	1.078441	130.5416	178.3203	4.561579	-2.01619	-4.12369
N	1.342926	72.14512	0.152534	4.536866	-0.7655	-2.35759
N	1.33912	108.4059	-0.20901	6.40549	-1.45522	-1.48302
C	1.48088	125.3949	-179.313	7.545066	-1.60689	-0.54955
H	1.093701	107.0965	-128.819	8.460034	-1.52298	-1.14283
H	1.09244	106.5426	-13.7973	7.508723	-0.75028	0.127447
C	1.530889	112.7924	108.4299	7.50494	-2.92623	0.225912
H	1.097455	109.5802	58.51343	7.508526	-3.76739	-0.47896
H	1.097354	109.1016	-58.1453	6.563269	-2.98491	0.786266
C	1.536133	111.6269	-179.913	8.69662	-3.0547	1.186692
H	1.097363	109.2983	-57.8543	9.631512	-2.97857	0.617142
H	1.097664	109.2745	58.11816	8.691388	-2.20836	1.885659
C	1.53137	112.5201	-179.84	8.678773	-4.37003	1.970713
H	1.09498	111.3557	60.34695	8.722298	-5.23456	1.300141
H	1.092912	110.7686	-179.914	9.53657	-4.43082	2.64521

H	1.095266	111.3517	-60.132	7.771596	-4.46015	2.577768
C	1.478567	125.6084	-177.004	3.308391	0.022818	-2.5933
H	1.093093	107.6742	9.855153	3.306809	0.854793	-1.88431
H	1.093165	106.6116	125.6131	3.391477	0.436975	-3.60156
C	1.535028	113.6923	-113.115	2.016356	-0.79671	-2.46948
H	1.096137	106.3987	-164.756	1.222786	-0.19428	-2.92648
H	1.09757	109.645	-50.3892	2.096945	-1.7068	-3.07768
C	1.536726	114.4244	74.54727	1.620073	-1.14403	-1.02593
H	1.095627	109.4142	61.04598	1.450095	-0.21986	-0.46254
H	1.097088	110.1211	-56.5425	2.437426	-1.6813	-0.52905
C	1.53481	110.5835	-177.724	0.352148	-2.00871	-1.00762
H	1.09281	110.7885	-57.1572	-0.47031	-1.50197	-1.51851
H	1.09487	110.8058	62.11019	0.522993	-2.96041	-1.52127
C	2.500203	124.8402	-149.804	-1.34661	-2.0142	0.826834
C	2.217237	65.17333	-72.4011	0.576586	-2.99309	1.336012
H	1.093757	99.14477	109.605	-2.17035	-1.52153	0.302413
H	1.077589	157.5612	-1.33982	1.579906	-3.35623	1.185426
C	1.362242	71.93001	-179.083	-0.24709	-3.09225	2.416486
H	1.078407	130.6582	179.4874	-0.08951	-3.54909	3.380553
N	1.343711	28.85629	-70.8136	-0.12482	-2.31193	0.353384
N	1.33801	137.2058	-73.8353	-1.43899	-2.47919	2.078044
C	1.483185	124.8688	-179.979	-2.62399	-2.32141	2.955943
H	1.09382	106.9101	131.6155	-2.84077	-3.30697	3.377992
H	1.092526	105.8461	15.8097	-3.44672	-2.03287	2.297553
C	1.53092	112.2633	-106.104	-2.39538	-1.28357	4.057919
H	1.097857	109.8548	-57.551	-1.5218	-1.56544	4.660166
H	1.097527	108.9618	59.14625	-2.16264	-0.31642	3.594222
C	1.535947	111.6689	-179.29	-3.62395	-1.14383	4.969086
H	1.097543	109.3184	58.36215	-3.85488	-2.11786	5.419138
H	1.097499	109.246	-57.5781	-4.496	-0.87083	4.361217
C	1.531276	112.5336	-179.66	-3.41961	-0.10386	6.074308
H	1.093028	110.855	179.9366	-4.30919	-0.02599	6.704626
H	1.095076	111.3478	-60.2716	-2.57705	-0.36993	6.721209
H	1.095318	111.3266	60.10557	-3.22088	0.888576	5.655629
C	5.416871	106.3628	36.09212	4.394219	5.068682	-0.94931
C	1.540389	69.79059	-20.4738	5.376407	4.074827	-0.30096
C	1.54393	114.362	-103.483	5.072712	3.752471	1.178089
C	1.551403	47.56015	-179.531	3.122568	4.414373	-1.55067
C	1.527103	113.9844	147.8797	5.451199	2.332787	1.594367
H	1.095694	143.3047	-117.776	4.100809	5.840208	-0.22875
H	1.097166	108.834	18.96608	5.373403	3.143999	-0.88175

H	1.097884	110.8063	25.67426	4.007393	3.900156	1.39862
H	1.095676	110.851	57.92557	6.516547	2.145978	1.419316
H	1.093733	109.4366	-39.916	3.411307	3.720849	-2.34559
H	1.094499	107.875	84.57724	4.887978	5.591835	-1.7742
H	1.095052	109.1405	134.3544	6.392977	4.475313	-0.374
H	1.096089	110.0728	-90.0019	5.597945	4.457791	1.832356
H	1.09369	108.9485	-158.392	2.492172	5.195925	-1.98418
H	1.098049	110.1331	176.7164	5.263669	2.190735	2.666918
C	1.511238	114.0346	80.43171	2.297846	3.638184	-0.55007
O	1.439428	112.4363	-60.8268	4.738491	1.331962	0.844467
O	1.23266	124.6639	-97.5327	2.354726	2.417084	-0.39154
C	2.403064	143.7183	79.46919	0.678464	3.858336	1.21171
H	1.09184	128.448	-35.164	0.587758	4.652868	1.955085
H	1.092815	84.45127	-145.576	1.200179	3.005845	1.653656
C	1.523369	108.1641	103.5357	-0.68271	3.455914	0.658601
H	1.096219	108.9159	-84.0755	-1.12984	4.319188	0.152117
H	1.097265	109.0611	32.38908	-0.54473	2.674848	-0.09961
C	1.535086	111.7732	154.3378	-1.6219	2.956147	1.765235
H	1.099385	109.462	-56.6119	-1.1489	2.117625	2.296087
H	1.097183	109.2672	59.08751	-1.75681	3.749359	2.511173
C	1.53201	112.8861	-179.028	-2.99141	2.521922	1.233313
H	1.096286	111.6213	62.13261	-2.90555	1.680816	0.53545
H	1.094507	110.9583	-177.578	-3.64907	2.212453	2.051637
H	1.09503	110.9973	-58.1058	-3.48629	3.344236	0.706068
O	1.331189	111.7886	80.8094	1.518255	4.438073	0.174144
H	0.972715	108.3624	-35.3486	3.841521	1.663459	0.66634
C	6.036607	102.567	35.91699	-7.21391	-2.78207	-0.93778
C	1.531518	130.7305	9.816224	-7.23623	-3.19813	-2.41153
C	1.532309	114.4996	-63.2264	-6.45592	-2.26791	-3.34629
C	1.536782	15.94554	-7.30786	-5.81729	-2.66896	-0.30664
C	1.525007	114.462	62.65763	-4.96004	-2.19388	-3.05908
H	1.095516	102.1714	-121.367	-7.72937	-1.82506	-0.80147
H	1.099083	109.2884	58.83788	-6.82888	-4.21436	-2.50809
H	1.096681	111.2721	-61.8977	-6.87679	-1.25521	-3.34206
H	1.097836	110.4687	44.10915	-4.55386	-3.19751	-2.87745
H	1.099921	110.7915	115.9501	-5.22228	-3.57121	-0.51091
H	1.095289	95.8745	130.6263	-7.77948	-3.51914	-0.35768
H	1.095222	108.9552	174.2854	-8.2765	-3.25578	-2.74921
H	1.097017	108.9147	-178.369	-6.55653	-2.63251	-4.37605
H	1.092227	109.1881	-1.15098	-5.91829	-2.60108	0.778792
H	1.091852	110.2666	163.218	-4.43126	-1.76873	-3.91452

C	1.521697	117.5297	-120.379	-4.96457	-1.47022	-0.69583
O	1.354049	120.118	-64.827	-4.53965	-1.32473	-1.97322
O	1.353185	116.5062	154.1978	-4.03077	-1.07668	0.200976
C	2.726871	119.555	34.01789	-6.16385	0.925701	-1.20288
H	1.094938	95.27386	-54.7936	-6.84994	0.53615	-1.96211
H	1.09693	86.41842	-162.557	-5.22994	1.209225	-1.70355
C	1.524684	140.7662	77.07117	-6.79151	2.109576	-0.47545
H	1.096521	108.7359	-64.7888	-7.68907	1.763816	0.051045
H	1.098278	109.1527	51.14937	-6.09545	2.469967	0.293859
C	1.535547	112.3412	173.1829	-7.15123	3.257201	-1.43016
H	1.098684	109.5062	-57.6262	-6.25005	3.583917	-1.96703
H	1.097961	109.2776	58.23185	-7.84541	2.887346	-2.19622
C	1.531588	112.7042	-179.873	-7.7797	4.452397	-0.70746
H	1.095831	111.316	60.17686	-7.09702	4.868761	0.041831
H	1.093709	111.0987	-179.775	-8.02883	5.251552	-1.41137
H	1.095194	111.2153	-59.8464	-8.70193	4.163703	-0.19209
O	1.442173	32.45145	60.10381	-5.91295	-0.13359	-0.25693
H	1.206868	119.1196	80.16576	-4.86111	-0.15597	0.334401

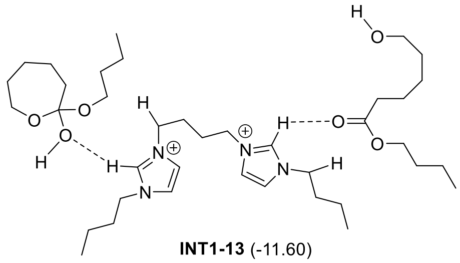

INT1-13 (-11.60)

Atom	Bond	Angle	Dihedral	X	Y	Z
C				-4.61737	1.168684	-1.09463
H	1.095007			-4.65097	0.411751	-0.30408
C	2.214305	161.9857		-5.16329	2.860857	-2.41435
H	1.078498	157.1675	3.693156	-5.75423	3.685488	-2.78032
C	1.362108	72.15817	-174.615	-4.00863	2.316597	-2.88962
H	1.078169	130.4464	178.3305	-3.41702	2.575217	-3.75308
N	1.342638	72.167	0.231955	-3.67922	1.263998	-2.05038
N	1.339245	108.4205	-0.31688	-5.52672	2.130857	-1.29679
C	1.480742	125.3419	-179.162	-6.71534	2.377845	-0.44898
H	1.093727	107.0651	-129.541	-7.57966	2.434843	-1.11676
H	1.092414	106.5322	-14.4767	-6.83811	1.492883	0.179625
C	1.530834	112.8501	107.7213	-6.5864	3.64446	0.401034
H	1.097507	109.5978	57.93511	-6.42017	4.510712	-0.25203

H	1.097323	109.1073	-58.752	-5.70192	3.556696	1.044546
C	1.536052	111.5753	179.4839	-7.83906	3.879236	1.258461
H	1.09746	109.2755	-57.9532	-8.71752	3.950715	0.604528
H	1.097652	109.2848	57.99774	-8.0053	3.007061	1.903841
C	1.531256	112.5475	-179.929	-7.73447	5.142724	2.117182
H	1.095017	111.3516	60.30725	-7.60644	6.036435	1.497544
H	1.09291	110.7661	-179.962	-8.63905	5.281125	2.714697
H	1.095271	111.364	-60.1789	-6.88573	5.087035	2.807229
C	1.480057	125.6414	-176.777	-2.53229	0.345481	-2.22774
H	1.093875	107.5551	3.740444	-2.63085	-0.45246	-1.48602
H	1.093582	106.6967	119.3099	-2.63893	-0.10241	-3.21968
C	1.534001	113.3462	-119.679	-1.17113	1.043953	-2.11576
H	1.095574	107.2503	-163.649	-0.42296	0.368211	-2.54458
H	1.097726	109.4277	-49.1074	-1.16748	1.94032	-2.74941
C	1.537449	114.8112	75.62726	-0.74474	1.400094	-0.6822
H	1.095931	109.1923	64.8809	-0.59712	0.4769	-0.11037
H	1.097223	110.7288	-52.825	-1.53091	1.972478	-0.17406
C	1.5358	109.6725	-174.628	0.560742	2.208308	-0.71679
H	1.093321	110.2271	-48.7672	1.294709	1.713589	-1.35859
H	1.094618	110.8208	70.90889	0.385408	3.213944	-1.11194
C	2.49654	123.7144	-140.524	2.418939	1.910237	0.923624
C	2.21331	65.49995	-75.2367	0.662917	3.001204	1.714142
H	1.09008	96.31276	106.1358	3.095599	1.390582	0.245123
H	1.077821	157.5064	-1.30581	-0.31152	3.461671	1.702223
C	1.362998	71.97924	-179.235	1.595917	2.92942	2.705163
H	1.078425	130.6887	179.3912	1.580514	3.309724	3.714188
N	1.342324	29.14437	-74.0672	1.192787	2.357147	0.609527
N	1.338826	137.707	-76.4594	2.683997	2.247523	2.191866
C	1.481662	125.4744	179.9652	3.925964	1.91528	2.928381
H	1.09363	107.0227	130.6628	4.281839	2.840727	3.389821
H	1.092428	106.5769	15.6258	4.661323	1.613235	2.179105
C	1.531067	112.6391	-106.702	3.714949	0.817549	3.974624
H	1.097436	109.5388	-57.9494	2.941394	1.132729	4.68641
H	1.097264	109.144	58.68628	3.340048	-0.086	3.47759
C	1.536211	111.6271	-179.492	5.013133	0.500469	4.732332
H	1.097419	109.3273	57.86345	5.385758	1.414136	5.212636
H	1.097566	109.3152	-58.1454	5.785315	0.191563	4.016117
C	1.531353	112.4411	179.797	4.821923	-0.59252	5.787731
H	1.092902	110.8096	179.9603	5.760336	-0.79712	6.309222
H	1.094951	111.3325	-60.2492	4.082362	-0.29601	6.538763
H	1.095392	111.3034	60.1789	4.483011	-1.52974	5.333161

C	5.402798	80.4136	70.2418	2.871328	-2.73278	-1.80181
C	1.538932	131.5907	148.3582	4.093047	-3.60793	-2.13321
C	1.534749	115.3351	-72.4415	5.042442	-3.03552	-3.19455
C	1.537842	18.33594	133.8184	3.227905	-1.41508	-1.09366
C	1.524411	114.7364	52.00963	5.509821	-1.60894	-2.92947
H	1.096788	101.6921	16.61469	2.293127	-2.51572	-2.70818
H	1.098912	108.3777	50.32991	4.653372	-3.78649	-1.2049
H	1.096669	110.2171	-69.594	4.561418	-3.04875	-4.18
H	1.096206	112.5892	52.10293	5.929426	-1.48317	-1.9246
H	1.094838	110.1705	128.5564	4.027351	-1.58261	-0.36463
H	1.095891	95.38979	-91.4198	2.209496	-3.30462	-1.14154
H	1.095615	108.9446	165.2336	3.745611	-4.59089	-2.47003
H	1.096716	109.2837	173.7066	5.928376	-3.67732	-3.27194
H	1.096075	110.3365	10.95658	2.364391	-1.03763	-0.53396
H	1.092641	110.6252	172.7508	6.276617	-1.31496	-3.65021
C	1.533378	114.3623	-108.9	3.658175	-0.28597	-2.03771
O	1.391525	115.9373	-37.6273	4.429423	-0.66764	-3.13126
O	1.418636	109.1092	79.49416	2.503501	0.301094	-2.61617
C	2.433931	138.3189	-160.37	4.879831	1.81585	-1.91966
H	1.09862	96.72039	-119.856	4.059997	2.460631	-2.26476
H	1.093609	86.54413	132.5905	5.427714	1.470556	-2.8009
C	1.522037	139.6223	14.3647	5.805921	2.567135	-0.97387
H	1.099991	109.6683	-70.0554	5.239689	2.908412	-0.09473
H	1.096844	108.8048	46.25865	6.572117	1.874421	-0.60487
C	1.535585	112.3774	168.2584	6.472732	3.77598	-1.64624
H	1.097216	109.1082	-58.7116	7.046444	3.432024	-2.51597
H	1.098213	109.4532	57.02022	5.698859	4.449457	-2.03818
C	1.531253	112.8547	179.3208	7.394386	4.549733	-0.69935
H	1.095115	111.2714	59.8416	8.19997	3.912213	-0.32004
H	1.093266	111.0918	179.725	7.856847	5.39985	-1.20794
H	1.095988	111.3512	-60.3835	6.842961	4.940239	0.163568
O	1.425008	106.0129	-162.202	4.333013	0.670336	-1.22486
H	0.969561	105.8025	-137.346	2.716256	0.434896	-3.55259
C	5.356621	104.5426	38.58355	-4.51442	-4.50862	-1.13125
C	1.540786	69.66801	-18.1244	-5.43798	-3.41285	-0.56526
C	1.544223	114.2983	-107.475	-5.39452	-3.27696	0.972363
C	1.551002	49.10022	-172.786	-3.06007	-4.04328	-1.40314
C	1.52702	114.2225	147.8909	-5.61879	-1.85516	1.482243
H	1.09556	146.7942	-114.421	-4.49824	-5.37552	-0.46157
H	1.0973	108.8296	15.02906	-5.16908	-2.45217	-1.02224
H	1.097482	110.7766	25.4902	-4.43521	-3.63444	1.367818

H	1.095841	110.8154	58.05633	-6.58416	-1.46619	1.13925
H	1.09361	109.561	-36.4266	-3.06317	-3.25772	-2.16398
H	1.094498	104.5085	87.97603	-4.9072	-4.86692	-2.08795
H	1.095053	109.1456	130.3937	-6.46871	-3.61752	-0.87323
H	1.096095	109.9877	-90.0299	-6.14813	-3.92982	1.427639
H	1.093811	108.9745	-155.003	-2.48041	-4.89036	-1.78113
H	1.097901	110.072	176.7213	-5.63239	-1.8463	2.580024
C	1.511454	113.728	83.78333	-2.36079	-3.48885	-0.18326
O	1.438631	112.6642	-60.696	-4.62359	-0.93197	1.005842
O	1.231101	124.6722	-98.9806	-2.2418	-2.29029	0.07154
C	2.405316	143.4618	77.04559	-1.26305	-4.11072	1.864612
H	1.091872	127.9949	-36.2001	-1.51292	-4.93278	2.538388
H	1.092635	84.04646	-146.246	-1.69752	-3.18354	2.245951
C	1.523085	108.9768	103.0126	0.243256	-3.98604	1.676766
H	1.096418	108.8693	-84.2426	0.620381	-4.9062	1.215021
H	1.097786	108.9086	32.2643	0.447754	-3.16546	0.976797
C	1.535454	111.8758	154.0813	0.966811	-3.73404	3.007398
H	1.098534	109.4771	-57.2718	0.563011	-2.8272	3.477895
H	1.097059	109.3424	58.56017	0.751557	-4.55742	3.699675
C	1.531272	112.7785	-179.627	2.482768	-3.59617	2.841081
H	1.097139	111.2539	59.83476	2.7356	-2.76071	2.176403
H	1.094145	111.1466	-179.824	2.971189	-3.42066	3.804302
H	1.094824	111.291	-60.0509	2.919645	-4.50331	2.411104
O	1.332936	111.6187	79.01237	-1.92027	-4.46141	0.614716
H	0.972558	108.6695	-36.7599	-3.76523	-1.38817	0.974775

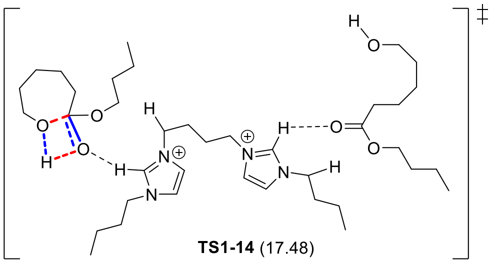

TS1-14 (17.48)

Atom	Bond	Angle	Dihedral	X	Y	Z
C				4.356223	-1.46413	-1.09174
H	1.094578			4.320559	-0.72978	-0.28083
C	2.21469	162.1439		4.992096	-3.14242	-2.38936
H	1.078503	157.1677	-0.44321	5.607777	-3.96242	-2.72358
C	1.36218	72.13005	-178.497	3.873378	-2.59114	-2.93717
H	1.078414	130.5547	178.4804	3.34012	-2.83904	-3.84114

N	1.342396	72.15138	0.088385	3.488918	-1.54601	-2.11306
N	1.33975	108.399	-0.06638	5.277628	-2.42515	-1.24133
C	1.480151	125.4199	-179.67	6.409008	-2.67951	-0.32148
H	1.093719	107.0387	-130.171	7.312472	-2.74313	-0.93461
H	1.09247	106.5851	-15.2136	6.500703	-1.79438	0.312269
C	1.530982	112.9676	107.0996	6.221398	-3.94407	0.520891
H	1.097411	109.5882	57.87869	6.089633	-4.81019	-0.14
H	1.097339	109.1206	-58.8026	5.300251	-3.85027	1.109824
C	1.535943	111.525	179.4352	7.419069	-4.18366	1.452179
H	1.097451	109.2784	-58.0707	8.33447	-4.26314	0.852076
H	1.097722	109.2864	57.87046	7.552425	-3.31009	2.103396
C	1.531345	112.5704	179.9474	7.255636	-5.4432	2.307683
H	1.094992	111.3481	60.27934	7.158453	-6.3384	1.68465
H	1.092922	110.7843	-179.982	8.122277	-5.5853	2.958237
H	1.09529	111.3555	-60.1796	6.367759	-5.37945	2.94586
C	1.480068	125.885	-177.554	2.346605	-0.63683	-2.3562
H	1.093708	107.6369	12.30068	2.465146	0.227834	-1.69703
H	1.093292	106.4402	127.5246	2.445116	-0.28491	-3.3866
C	1.533891	113.3839	-111.601	0.981534	-1.30949	-2.16403
H	1.095607	107.1847	-165.316	0.230288	-0.65204	-2.6154
H	1.097642	109.3658	-50.6066	0.95304	-2.24532	-2.73694
C	1.537077	114.8375	74.14809	0.587902	-1.5648	-0.70031
H	1.095801	109.1703	63.89973	0.481912	-0.60439	-0.18343
H	1.097402	110.796	-53.9635	1.371174	-2.12965	-0.17904
C	1.534424	109.4455	-176.03	-0.74351	-2.3262	-0.65474
H	1.094623	109.9377	-51.3711	-1.48834	-1.81288	-1.27112
H	1.094595	110.9588	69.13955	-0.62224	-3.34941	-1.02415
C	2.497139	124.0099	-142.663	-2.52081	-1.93087	1.054246
C	2.209643	65.61428	-74.4956	-0.76125	-3.0356	1.806628
H	1.084279	96.57462	106.6519	-3.21265	-1.416	0.397045
H	1.077756	157.4983	-1.00017	0.201908	-3.51784	1.770304
C	1.363398	72.01048	-179.019	-1.65137	-2.91276	2.832029
H	1.07837	130.7197	179.4196	-1.60321	-3.26221	3.85107
N	1.341196	29.17388	-73.0784	-1.31974	-2.41415	0.703991
N	1.338946	137.9375	-76.0546	-2.74425	-2.22246	2.341811
C	1.481666	125.5317	-179.425	-3.95134	-1.84439	3.113388
H	1.093645	107.0028	129.5196	-4.31685	-2.75265	3.600738
H	1.092583	106.5762	14.51867	-4.70292	-1.53353	2.383839
C	1.531059	112.7343	-107.82	-3.67789	-0.73718	4.134884
H	1.097394	109.5788	-58.064	-2.89186	-1.06242	4.82818
H	1.097315	109.1669	58.55477	-3.29328	0.147903	3.612568

C	1.536121	111.581	-179.608	-4.94341	-0.37296	4.925751
H	1.097432	109.2848	57.78417	-5.32621	-1.26937	5.429997
H	1.097577	109.3087	-58.1842	-5.72792	-0.05306	4.227986
C	1.531416	112.4726	179.7299	-4.69119	0.729016	5.958836
H	1.092901	110.7717	-179.894	-5.60757	0.964636	6.505785
H	1.094985	111.317	-60.1499	-3.93681	0.423011	6.691143
H	1.095215	111.3644	60.29757	-4.34232	1.651004	5.481633
C	5.189751	80.58245	67.81973	-2.79059	2.57356	-1.50911
C	1.537118	150.7736	97.68174	-3.44037	3.476533	-2.56985
C	1.534495	116.1392	-40.1726	-3.84734	2.793013	-3.88204
C	1.53579	39.87457	137.7308	-3.71251	1.512494	-0.89036
C	1.523615	115.519	62.16033	-4.89929	1.698394	-3.75318
H	1.096695	75.31385	-8.13957	-1.90708	2.071467	-1.92148
H	1.098542	108.9253	83.2323	-4.3231	3.962189	-2.132
H	1.098513	110.5823	-61.8654	-2.96655	2.377943	-4.39062
H	1.095245	111.9731	46.83396	-5.7423	2.011711	-3.1281
H	1.095201	111.7134	160.4846	-4.71819	1.906059	-0.70819
H	1.096073	97.31214	-113.427	-2.4337	3.217673	-0.69725
H	1.09593	108.337	-162.05	-2.74098	4.285655	-2.80912
H	1.096744	108.6439	-177.744	-4.25724	3.551442	-4.55999
H	1.097635	109.2501	43.67572	-3.32099	1.221996	0.093059
H	1.093797	110.9869	167.2452	-5.2971	1.420036	-4.73331
C	1.522382	115.5555	-73.9661	-3.82075	0.205324	-1.66318
O	1.448092	114.095	-74.5782	-4.3993	0.461234	-3.19068
O	1.334277	119.24	43.42818	-2.71518	-0.34968	-2.16317
C	2.42101	140.0815	-171.266	-5.10446	-1.84732	-1.66923
H	1.09536	90.05108	-143.058	-4.20782	-2.40838	-1.95395
H	1.09307	93.25294	108.8446	-5.64227	-1.58143	-2.58294
C	1.52033	138.4719	-18.5003	-5.99438	-2.63598	-0.7219
H	1.099978	109.7416	-58.7151	-5.43046	-2.89899	0.185172
H	1.096566	108.8633	57.6266	-6.82486	-1.99614	-0.40039
C	1.535712	112.1985	179.5007	-6.54127	-3.91619	-1.37027
H	1.097188	109.103	-58.481	-7.11015	-3.64851	-2.26946
H	1.098309	109.5122	57.27442	-5.7048	-4.54	-1.71299
C	1.531265	112.818	179.6126	-7.43233	-4.72672	-0.42483
H	1.094989	111.25	59.86673	-8.29761	-4.14174	-0.09606
H	1.093259	111.0563	179.7419	-7.80897	-5.62781	-0.91614
H	1.095959	111.3815	-60.3515	-6.88311	-5.04203	0.46963
O	1.377728	108.3454	-178.041	-4.71129	-0.61478	-1.00552
H	1.17518	128.2443	-28.9446	-3.31135	0.021274	-3.25263
C	5.518629	128.5037	-120.021	5.093803	4.001254	-0.88957

C	1.540167	46.66056	-57.6347	5.645192	2.828698	-0.05698
C	1.544684	114.4886	-109.115	5.085894	2.745342	1.380477
C	1.550767	71.10487	144.9644	3.75244	3.701759	-1.60785
C	1.527507	114.0245	146.4744	4.934896	1.319541	1.907325
H	1.09574	144.4478	-117.853	4.980388	4.893577	-0.26383
H	1.09756	108.6051	13.38359	5.429189	1.891582	-0.58594
H	1.097689	110.7488	24.20257	4.108231	3.24003	1.446653
H	1.095836	110.7575	58.20433	5.897257	0.795649	1.891382
H	1.093737	109.5332	-42.6783	3.886987	2.86567	-2.30003
H	1.094491	107.467	43.19965	5.810487	4.265252	-1.67352
H	1.095083	109.0818	128.6424	6.736451	2.906135	-0.00837
H	1.096042	110.0477	-91.4314	5.732535	3.302383	2.068132
H	1.093823	108.9184	-161.172	3.453493	4.583025	-2.18269
H	1.098143	110.0957	176.7644	4.588964	1.337014	2.949411
C	1.51149	113.9378	77.69282	2.623252	3.337657	-0.67138
O	1.438071	112.5793	-60.6212	4.033035	0.52993	1.112838
O	1.231653	124.984	-98.6795	2.254458	2.189093	-0.42286
C	2.391013	144.3988	78.30446	0.99949	4.213988	0.84927
H	1.095389	92.74403	-121.408	1.382998	3.618159	1.684606
H	1.094853	90.76036	130.616	0.208173	3.637203	0.359535
C	1.518686	137.1041	5.686016	0.521397	5.583547	1.298872
H	1.096333	109.0319	-59.6827	1.364795	6.130933	1.7359
H	1.096539	109.0009	56.39883	0.197078	6.154514	0.420686
C	1.535169	112.0994	178.4229	-0.62401	5.489999	2.316735
H	1.099248	109.3979	-57.9261	-1.46022	4.92977	1.874887
H	1.098503	109.4179	58.06352	-0.29181	4.909243	3.187986
C	1.531549	112.6324	-179.931	-1.1169	6.864966	2.777345
H	1.094959	111.26	60.16072	-1.49079	7.455532	1.934507
H	1.093644	111.1576	-179.751	-1.9286	6.770203	3.504134
H	1.094786	111.1642	-59.8213	-0.31141	7.435275	3.251139
O	1.330224	111.9652	79.82101	2.078367	4.41221	-0.10753
H	0.973456	108.2146	-36.6146	3.30699	1.105506	0.814197

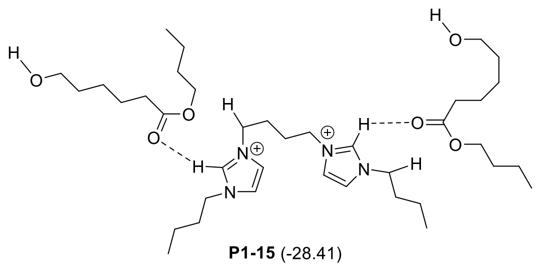

P1-15 (-28.41)

Atom	Bond	Angle	Dihedral	X	Y	Z
C				5.946898	-0.62845	-0.75387
H	1.090523			5.699267	-0.10122	0.168064
C	2.212309	163.6391		6.886853	-1.95669	-2.25272
H	1.078529	157.1349	4.133101	7.557761	-2.7128	-2.62876
C	1.362619	72.17648	-174.069	5.968091	-1.18432	-2.89774
H	1.078129	130.4371	178.1566	5.70335	-1.13747	-3.94181
N	1.342199	72.21158	0.251318	5.386602	-0.36427	-1.94457
N	1.338695	108.546	-0.30382	6.860084	-1.59351	-0.91772
C	1.480835	125.5259	-179.063	7.688067	-2.18619	0.157472
H	1.093524	106.9684	-132.663	8.718595	-2.20649	-0.20776
H	1.092562	106.6532	-17.7797	7.650408	-1.49205	1.000343
C	1.530832	112.8092	104.6163	7.215818	-3.58317	0.568414
H	1.097522	109.5305	58.01528	7.227581	-4.24642	-0.30595
H	1.097476	109.1064	-58.6112	6.17417	-3.52451	0.908975
C	1.536131	111.6334	179.6242	8.098117	-4.17576	1.677507
H	1.097368	109.2799	-58.1503	9.137767	-4.21933	1.329016
H	1.097644	109.3008	57.80176	8.091737	-3.50281	2.544635
C	1.531254	112.5295	179.8659	7.641546	-5.57288	2.106852
H	1.095035	111.3578	60.28461	7.673733	-6.27781	1.269511
H	1.092904	110.7597	-179.984	8.287742	-5.96623	2.895614
H	1.095306	111.3513	-60.2195	6.617133	-5.55616	2.494142
C	1.479803	124.8061	-175.624	4.396862	0.707366	-2.19322
H	1.091317	106.9366	-0.36191	4.224697	1.208703	-1.23929
H	1.093704	106.8804	115.2095	4.867818	1.423159	-2.87295
C	1.53286	114.0628	-123.412	3.07828	0.212261	-2.7981
H	1.096403	105.6588	-170.861	2.523057	1.115585	-3.07708
H	1.097649	109.9223	-56.9979	3.274646	-0.32224	-3.73649
C	1.536854	115.2574	68.4853	2.210229	-0.6464	-1.86477
H	1.095021	109.537	55.71788	2.008568	-0.09395	-0.94108
H	1.097027	110.3912	-62.8791	2.736887	-1.57032	-1.59554
C	1.535234	109.9202	176.1507	0.884152	-0.99351	-2.55613
H	1.092459	110.5668	-62.1621	0.315953	-0.08624	-2.77401

H	1.0944	111.1154	57.06975	1.05777	-1.50823	-3.5062
C	2.503589	119.3387	-153.678	-1.13862	-1.47131	-1.1604
C	2.217704	64.96939	-85.5507	0.190337	-3.21132	-1.51316
H	1.097371	97.12763	95.23939	-1.56569	-0.46086	-1.18919
H	1.078113	157.3725	-1.93016	1.033108	-3.75607	-1.90726
C	1.361852	71.95439	-178.942	-0.86696	-3.63276	-0.7654
H	1.078396	130.687	179.1236	-1.11059	-4.61017	-0.38036
N	1.343621	28.71646	-84.0964	0.005238	-1.85906	-1.74909
N	1.338509	137.0034	-87.2444	-1.68431	-2.53565	-0.55957
C	1.482143	125.612	-179.48	-2.94258	-2.53372	0.223708
H	1.093868	106.6492	133.6833	-3.52379	-3.39637	-0.1148
H	1.093032	107.3229	18.47759	-3.49553	-1.63177	-0.05095
C	1.531545	112.1661	-104.462	-2.68448	-2.59996	1.731895
H	1.09769	109.6155	-57.7848	-2.10203	-3.50021	1.966894
H	1.097233	109.0327	58.79054	-2.07146	-1.73951	2.028134
C	1.535217	111.7948	-179.545	-3.99327	-2.60879	2.534311
H	1.097949	109.3316	57.266	-4.61168	-3.45673	2.211697
H	1.097434	109.0068	-58.8249	-4.56236	-1.7002	2.299908
C	1.531481	112.5971	179.198	-3.75678	-2.69455	4.04499
H	1.093122	110.8807	179.772	-4.70524	-2.69487	4.588432
H	1.094952	111.3529	-60.456	-3.22123	-3.61112	4.313296
H	1.095293	111.3337	59.89593	-3.16728	-1.84448	4.40491
C	6.062233	114.019	100.7443	-5.00932	3.176506	-0.75274
C	1.545399	76.28047	48.85976	-4.72761	3.065976	-2.26822
C	1.54086	113.9469	-57.8408	-3.23757	3.19611	-2.6385
C	1.5295	97.71567	161.2563	-6.23635	2.382865	-0.3012
C	1.530273	113.8386	87.07527	-2.4701	1.873141	-2.58878
H	1.093868	45.13483	-89.756	-4.14095	2.842719	-0.17735
H	1.098853	110.1372	64.33618	-5.09994	2.106487	-2.65323
H	1.096975	110.2434	-36.6046	-2.74764	3.931191	-1.98813
H	1.101403	109.1203	62.16321	-2.90146	1.177892	-3.3261
H	1.095405	112.0435	-130.727	-7.12385	2.624177	-0.89621
H	1.094837	147.0145	-55.5926	-5.15656	4.228437	-0.48736
H	1.094842	108.853	179.499	-5.30492	3.834521	-2.79237
H	1.096969	109.7613	-153.617	-3.14184	3.574388	-3.66372
H	1.09789	111.0058	111.6477	-6.50346	2.62711	0.735308
H	1.096463	109.5737	179.5681	-1.42437	2.044293	-2.87056
C	1.515632	113.4769	-8.09688	-6.03516	0.881384	-0.34829
O	1.435911	114.7564	-61.6205	-2.42929	1.249885	-1.29582
O	1.232571	125.1614	13.80637	-4.94773	0.314472	-0.47209
C	2.393742	143.9136	-168.225	-7.16199	-1.22643	-0.21643

H	1.095302	93.66759	-120.646	-6.57874	-1.56378	0.647103
H	1.094662	90.32701	131.0457	-6.65352	-1.56224	-1.12581
C	1.518901	136.8227	7.188118	-8.59987	-1.7116	-0.15186
H	1.096304	109.0189	-60.6635	-9.07486	-1.30267	0.747613
H	1.096303	108.9961	55.51976	-9.1508	-1.3103	-1.01054
C	1.53519	111.9594	177.4179	-8.68871	-3.24418	-0.14144
H	1.098504	109.4255	-58.2068	-8.20371	-3.64438	-1.04217
H	1.098782	109.4006	57.87406	-8.12203	-3.63681	0.714155
C	1.531678	112.621	179.7935	-10.1327	-3.75014	-0.07141
H	1.094905	111.202	60.05999	-10.7158	-3.40367	-0.93093
H	1.093579	111.1134	-179.951	-10.1672	-4.84315	-0.06446
H	1.094927	111.1919	-59.9475	-10.6331	-3.39474	0.835319
O	1.326107	111.6784	-167.434	-7.18171	0.229021	-0.2126
H	0.981151	108.6841	61.87752	-3.34653	1.036042	-1.02089
C	5.339273	129.1136	23.22774	3.52196	5.191669	0.569638
C	1.540168	62.45483	9.668295	4.772225	4.366741	0.928036
C	1.544438	114.45	-104.672	4.651157	3.559345	2.239044
C	1.551169	54.99411	-150.002	2.443517	4.414016	-0.22932
C	1.526602	114.1077	147.0688	5.409793	2.234634	2.227836
H	1.095774	144.8788	-77.0091	3.069973	5.61313	1.474513
H	1.096728	108.9251	18.06181	4.997099	3.683691	0.099974
H	1.098015	110.8648	24.76266	3.599278	3.3477	2.472267
H	1.095188	110.9288	56.57045	6.470431	2.393089	2.005629
H	1.093667	109.4896	-40.7971	2.868434	4.070121	-1.17658
H	1.094515	108.3887	112.7355	3.809722	6.043478	-0.05452
H	1.094961	109.0025	133.2988	5.632653	5.039354	1.006747
H	1.096071	110.0341	-90.9062	5.01347	4.157348	3.083137
H	1.093796	108.8916	-159.424	1.608611	5.08695	-0.44492
H	1.098264	110.0636	175.6168	5.343916	1.754374	3.213328
C	1.511592	113.876	79.42261	1.905183	3.199412	0.491678
O	1.439978	112.2184	-61.8997	4.920041	1.33523	1.215535
O	1.23153	124.3467	-98.0674	2.290651	2.046568	0.294118
C	2.41699	142.9448	77.96814	0.383994	2.510293	2.238944
H	1.094181	116.1954	-69.5729	0.676174	2.765386	3.262072
H	1.091579	80.38698	-176.294	0.810547	1.540601	1.975705
C	1.521944	122.7778	73.35526	-1.12861	2.538757	2.07305
H	1.096411	108.792	-79.902	-1.48121	3.565889	2.224014
H	1.097455	109.486	36.80172	-1.38954	2.256314	1.045164
C	1.535793	111.4241	159.0768	-1.82052	1.60237	3.074602
H	1.099782	109.4604	-57.2839	-1.43545	0.580166	2.946764
H	1.097461	109.3798	58.34596	-1.55195	1.899505	4.096365

C	1.531557	112.931	-179.649	-3.34541	1.597849	2.931962
H	1.095792	111.3818	61.00987	-3.65267	1.25354	1.93808
H	1.094389	111.1058	-178.566	-3.80877	0.941444	3.675007
H	1.095133	110.9846	-59.1323	-3.75619	2.602092	3.080541
O	1.331718	111.5999	80.27915	0.985073	3.533168	1.394716
H	0.972549	108.4796	-37.4686	3.953524	1.428878	1.16143

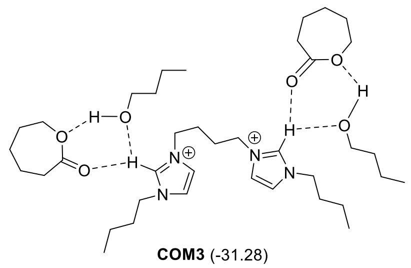

COM3 (-31.28)

Atom	Bond	Angle	Dihedral	X	Y	Z
C				3.686901	1.495699	-1.25763
H	1.085676			3.823928	0.752848	-0.47783
C	2.211024	163.0886		3.972582	2.758941	-3.04962
H	1.078473	157.0174	5.088556	4.442586	3.102754	-3.95736
C	1.362254	72.24816	-173.692	2.882044	3.227216	-2.3809
H	1.077679	130.4244	178.1495	2.239266	4.065853	-2.59283
N	1.343181	72.21321	0.370759	2.712924	2.420585	-1.26717
N	1.338688	108.5519	-0.3717	4.463124	1.683488	-2.33201
C	1.481934	125.7213	-178.686	5.634695	0.857546	-2.708
H	1.093675	106.385	-141.327	6.387915	1.549066	-3.09605
H	1.091429	106.5623	-26.2802	6.019515	0.424167	-1.78317
C	1.531302	112.5517	96.83158	5.288297	-0.21621	-3.74335
H	1.097781	109.715	57.12368	4.858563	0.255581	-4.63658
H	1.097685	109.1197	-59.5815	4.515484	-0.87755	-3.33068
C	1.535429	111.552	178.8873	6.52305	-1.03984	-4.1365
H	1.097627	109.2778	-58.016	7.295289	-0.36828	-4.53329
H	1.097521	109.2105	57.86573	6.949819	-1.50245	-3.23738
C	1.531268	112.6459	-179.988	6.204668	-2.12195	-5.17209
H	1.095127	111.3282	60.28386	5.811916	-1.68539	-6.09646
H	1.093107	110.8681	-179.952	7.102142	-2.6902	-5.42999
H	1.095398	111.3587	-60.1145	5.460435	-2.83055	-4.79275
C	1.480953	124.7713	-174.591	1.721206	2.613967	-0.18443
H	1.09333	106.3055	-8.03962	1.859689	1.78169	0.51092
H	1.094138	107.0195	107.3921	1.989084	3.539711	0.333623
C	1.532893	113.7811	-131.54	0.273947	2.706425	-0.68107

H	1.096369	106.0846	-169.946	-0.32299	3.027511	0.180673
H	1.097597	109.8693	-56.2509	0.183853	3.513256	-1.41974
C	1.535979	115.3587	68.96175	-0.31213	1.403926	-1.24608
H	1.095944	109.7712	58.76368	-0.28128	0.620633	-0.48019
H	1.097	110.4814	-58.8671	0.28949	1.047158	-2.09117
C	1.535812	110.3615	179.5589	-1.76362	1.622811	-1.69775
H	1.091823	111.9521	-56.325	-2.38908	2.006931	-0.88947
H	1.094314	110.7682	63.40536	-1.81003	2.346437	-2.51735
C	2.504124	115.8068	-148.398	-3.2822	-0.35678	-1.48378
C	2.215232	65.01192	-90.5032	-2.23671	-0.18534	-3.42924
H	1.086861	98.39321	90.32093	-3.64608	-0.16948	-0.47691
H	1.078122	157.2859	-2.09004	-1.59203	0.247705	-4.17704
C	1.361946	71.99949	-179.508	-3.02549	-1.29481	-3.47165
H	1.078171	130.7219	178.9933	-3.192	-2.00969	-4.26139
N	1.343017	28.67056	-89.9398	-2.40941	0.388936	-2.18081
N	1.338364	137.1103	-91.2221	-3.66869	-1.38354	-2.25032
C	1.48316	125.3177	-179.081	-4.63311	-2.43689	-1.85017
H	1.094145	106.8868	126.0449	-5.41073	-2.46464	-2.61938
H	1.092401	106.1666	10.13941	-5.08011	-2.09958	-0.91222
C	1.530787	112.3737	-111.774	-3.96714	-3.80573	-1.68866
H	1.097842	109.9549	-57.4056	-3.47007	-4.09329	-2.62433
H	1.09703	109.0053	59.37447	-3.18501	-3.73435	-0.92273
C	1.535533	111.57	-179.036	-4.98556	-4.88638	-1.29767
H	1.097648	109.3609	58.33104	-5.76921	-4.94227	-2.06422
H	1.097692	109.2463	-57.5477	-5.4872	-4.59106	-0.36705
C	1.531317	112.5702	-179.628	-4.34162	-6.26446	-1.12104
H	1.093154	110.8997	179.9655	-5.08953	-7.0116	-0.84282
H	1.09513	111.3346	-60.2332	-3.86371	-6.6025	-2.04658
H	1.095355	111.2976	60.08406	-3.57805	-6.24911	-0.33585
C	6.180108	141.2374	-37.8023	-6.90949	4.641064	-0.08409
C	1.53312	139.6525	-98.5644	-8.23573	4.384717	0.641042
C	1.533812	114.8988	-8.55316	-8.51375	2.914453	0.978105
C	1.547816	46.853	-21.8839	-5.65455	4.19242	0.703046
C	1.523517	114.7456	59.52489	-7.4714	2.257499	1.874223
H	1.097233	63.4085	170.6085	-6.91168	4.15849	-1.0695
H	1.09838	108.8145	113.7367	-8.25048	4.970465	1.570085
H	1.097255	111.0954	-63.9108	-8.61616	2.312779	0.066256
H	1.095285	112.2038	44.16045	-7.17849	2.90217	2.709839
H	1.097668	109.7246	-179.993	-5.7482	4.495844	1.753778
H	1.09449	111.1352	72.49041	-6.81175	5.715196	-0.27009
H	1.09497	108.8962	-131.024	-9.05613	4.769453	0.026316

H	1.096345	108.9342	179.3683	-9.47469	2.844984	1.501299
H	1.091275	110.3898	61.89321	-4.76181	4.676019	0.303012
H	1.090748	110.9099	165.1447	-7.84711	1.319782	2.285633
C	1.512443	114.0009	-56.6322	-5.37852	2.707363	0.62624
O	1.353651	119.4641	-64.2071	-6.2676	1.836468	1.15865
O	1.21837	123.4612	114.5178	-4.39546	2.233958	0.084117
C	6.054013	99.90262	-51.9333	8.714827	0.986698	2.502295
C	1.532837	142.0109	34.84165	8.517311	1.010533	4.022166
C	1.53384	114.876	-6.18325	7.060487	1.150007	4.481378
C	1.547435	48.7682	111.4555	7.978285	-0.16748	1.781236
C	1.524054	114.7726	60.02957	6.13471	0.031948	4.017028
H	1.097331	62.03647	-53.0902	8.410701	1.944009	2.060482
H	1.09837	108.8602	116.0957	8.936697	0.089568	4.449198
H	1.097371	111.0538	-63.401	6.635555	2.10897	4.158818
H	1.095241	112.1214	43.89117	6.592759	-0.95647	4.130055
H	1.097645	109.7313	176.887	8.116632	-1.10189	2.34032
H	1.094362	108.9433	-151.784	9.782148	0.876982	2.286848
H	1.094973	108.9148	-128.636	9.102663	1.834537	4.44329
H	1.096418	108.9354	179.8774	7.031776	1.1542	5.577412
H	1.091157	110.4088	58.80591	8.39493	-0.32081	0.78448
H	1.09086	110.8918	164.832	5.201584	0.039692	4.581999
C	1.511524	114.1336	-59.8067	6.502282	0.078948	1.568186
O	1.354721	119.7633	-64.5707	5.677266	0.186722	2.637299
O	1.218668	123.5015	114.5427	5.993394	0.205743	0.468137
C	3.586921	114.1549	-149.931	-4.79805	-1.20025	2.377155
H	1.09958	87.16763	171.3874	-5.78082	-1.43835	2.809068
H	1.099976	73.60047	62.25509	-4.3643	-0.38417	2.973657
C	1.521525	159.6487	-41.2583	-3.89793	-2.42549	2.437442
H	1.098143	108.6857	-81.6653	-4.33728	-3.21581	1.81432
H	1.099311	108.7281	34.1763	-2.92649	-2.17218	1.989532
C	1.534176	113.0308	156.3348	-3.69077	-2.94842	3.86479
H	1.098109	109.395	-57.6018	-3.26729	-2.14956	4.487953
H	1.097443	109.3013	58.17815	-4.66504	-3.19455	4.305915
C	1.53123	112.924	-179.813	-2.78094	-4.17865	3.92309
H	1.096311	111.2571	59.949	-1.78593	-3.9566	3.519922
H	1.093589	111.214	179.9562	-2.6519	-4.52701	4.951647
H	1.095428	111.2834	-60.1174	-3.19723	-5.00895	3.342349
O	1.434382	52.4467	-70.5535	-4.94862	-0.78847	1.011426
H	0.972729	107.9425	-5.8368	-5.46565	0.035417	1.002115
C	3.484936	102.4755	-142.105	2.796641	-1.43323	1.53161
H	1.099496	82.77966	149.8629	2.686611	-1.66937	2.599796

H	1.099303	71.88906	38.70342	3.667139	-1.99045	1.157173
C	1.521702	165.2384	-61.111	1.544134	-1.85777	0.778907
H	1.098921	108.5922	-84.7942	0.691429	-1.28181	1.164633
H	1.099185	108.7613	31.1692	1.659287	-1.58697	-0.28016
C	1.533931	112.8944	153.175	1.255105	-3.35933	0.900353
H	1.097456	109.3726	-57.8649	2.116562	-3.92727	0.526529
H	1.097411	109.3807	57.96098	1.151184	-3.62565	1.959873
C	1.531254	113.0113	-179.917	-0.00369	-3.78984	0.142181
H	1.096346	111.2244	59.59298	0.08077	-3.56474	-0.92748
H	1.093631	111.2164	179.5523	-0.17742	-4.86498	0.241788
H	1.096721	111.0591	-60.5978	-0.89131	-3.27283	0.526408
O	1.433695	57.61408	-90.7729	3.000655	-0.02489	1.357078
H	0.973001	106.9634	-4.76001	3.831499	0.199974	1.8108

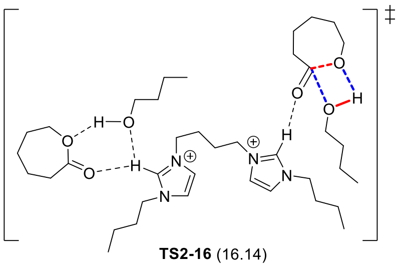

TS2-16 (16.14)

Atom	Bond	Angle	Dihedral	X	Y	Z
C				4.0873	-1.50863	-1.51571
H	1.087804			4.164778	-0.56039	-0.98831
C	2.213512	161.2458		4.583311	-3.52012	-2.29515
H	1.078429	157.1738	4.21956	5.161036	-4.42162	-2.42378
C	1.362228	72.07565	-174.259	3.40646	-3.13023	-2.85966
H	1.078036	130.4493	178.3208	2.778273	-3.62728	-3.5811
N	1.341647	36.41194	-179.709	3.109292	-1.87313	-2.35871
N	1.339421	108.5283	-0.1113	4.993381	-2.4937	-1.46358
C	1.479497	125.2715	-179.521	6.221054	-2.48244	-0.63798
H	1.093688	107.053	-131.093	7.049678	-2.76961	-1.29149
H	1.092351	106.414	-16.1156	6.380215	-1.4449	-0.33565
C	1.530986	112.9145	106.1629	6.129237	-3.40629	0.579383
H	1.097343	109.5438	58.13449	5.9284	-4.43262	0.247019
H	1.097414	109.1068	-58.4739	5.276536	-3.10055	1.198852
C	1.535949	111.6268	179.7101	7.420457	-3.37733	1.410677
H	1.097394	109.3378	-57.9664	8.266998	-3.67171	0.777447
H	1.097845	109.2665	57.98633	7.62064	-2.34718	1.733158
C	1.531445	112.5697	-179.959	7.352387	-4.29556	2.634417

H	1.094913	111.268	60.26745	7.190517	-5.33777	2.340417
H	1.093035	110.7985	179.9941	8.284295	-4.25283	3.20402
H	1.095289	111.3826	-60.1556	6.537874	-4.00525	3.306689
C	1.481067	125.5359	-175.596	1.964172	-1.0268	-2.76613
H	1.090995	106.6602	-0.01927	2.081252	-0.07266	-2.25021
H	1.093585	106.7381	115.8338	2.066336	-0.85498	-3.84129
C	1.532899	113.5779	-122.854	0.5991	-1.65572	-2.46477
H	1.096545	106.0645	-169.975	-0.14308	-1.02692	-2.97092
H	1.097971	109.8155	-56.1408	0.529966	-2.64183	-2.94263
C	1.536302	115.0888	69.03862	0.2447	-1.7597	-0.97353
H	1.095002	109.7922	57.20538	0.279485	-0.76638	-0.51402
H	1.097136	110.304	-60.1196	0.984785	-2.37635	-0.44843
C	1.535189	110.133	178.6464	-1.15294	-2.37181	-0.80398
H	1.091715	111.8945	-57.8981	-1.91785	-1.78964	-1.32151
H	1.094502	110.7716	61.8706	-1.18397	-3.38726	-1.2112
C	2.50586	117.2378	-150.082	-2.47623	-1.67028	1.205024
C	2.214882	64.9519	-87.6123	-1.12765	-3.39756	1.526806
H	1.08672	98.41928	93.2479	-3.02926	-0.85116	0.753189
H	1.078053	157.2694	-2.11662	-0.40934	-4.1595	1.27052
C	1.361942	72.01276	-179.438	-1.77953	-3.1493	2.696551
H	1.078221	130.7424	179.0212	-1.73062	-3.65109	3.649638
N	1.342794	28.6092	-86.9439	-1.57476	-2.46495	0.605903
N	1.338875	137.0536	-88.4165	-2.61527	-2.06972	2.475342
C	1.48289	125.4332	-179.006	-3.52165	-1.45544	3.475394
H	1.094142	106.8169	127.0679	-4.13052	-2.26451	3.889917
H	1.092179	106.2399	11.10133	-4.17195	-0.77521	2.921088
C	1.531039	112.3936	-110.779	-2.75852	-0.71747	4.578632
H	1.09785	109.9656	-56.4293	-2.05259	-1.40123	5.067929
H	1.097089	108.9741	60.4123	-2.1628	0.084455	4.125174
C	1.535618	111.4863	-178.085	-3.71418	-0.13026	5.627456
H	1.097543	109.3182	58.49413	-4.31174	-0.93897	6.067355
H	1.097691	109.2389	-57.3533	-4.42604	0.542416	5.13179
C	1.531347	112.5882	-179.486	-2.97729	0.624956	6.737258
H	1.093132	110.9147	-179.874	-3.68204	1.028916	7.468751
H	1.095122	111.3147	-60.0827	-2.28372	-0.03179	7.272926
H	1.095434	111.3238	60.24579	-2.40017	1.463676	6.332959
C	6.076632	139.3962	-35.0129	-6.55563	-2.37713	-3.5854
C	1.533123	140.0532	-113.489	-7.99583	-1.90387	-3.35667
C	1.534145	114.9374	-3.14536	-8.25481	-1.25722	-1.98978
C	1.547631	50.18117	-32.7335	-5.49321	-1.25617	-3.48606
C	1.524215	114.7848	59.66706	-7.41409	-0.01956	-1.69885

H	1.09735	60.0644	159.8806	-6.29991	-3.18157	-2.88421
H	1.098381	108.8089	119.1335	-8.26199	-1.186	-4.14423
H	1.097212	111.1239	-63.8432	-8.1045	-1.97815	-1.17643
H	1.095225	112.1522	44.03582	-7.36437	0.657315	-2.55843
H	1.097628	109.7507	-179.81	-5.84463	-0.36371	-4.01974
H	1.094665	111.0209	62.77306	-6.48075	-2.8104	-4.58788
H	1.09487	108.892	-125.631	-8.67272	-2.75547	-3.4805
H	1.096626	108.9271	179.4982	-9.30592	-0.94932	-1.93536
H	1.091252	110.3055	62.01813	-4.56217	-1.57389	-3.95835
H	1.090699	110.8942	164.9974	-7.82003	0.53637	-0.85282
C	1.512256	113.9716	-56.4793	-5.12202	-0.8854	-2.06773
O	1.353529	119.4857	-65.0852	-6.05324	-0.32689	-1.25969
O	1.218439	123.407	113.8124	-4.01787	-1.07981	-1.5906
C	5.677574	128.1031	161.3651	3.30601	2.732003	2.177727
C	1.535829	90.84585	27.72169	4.785526	2.679022	2.586379
C	1.536834	117.1839	0.970106	5.674561	1.648408	1.87272
C	1.545598	66.21293	144.5778	3.054049	3.132475	0.706329
C	1.529711	115.8218	64.37266	5.863375	1.858731	0.369347
H	1.096186	61.82009	-85.5866	2.811827	1.774093	2.377262
H	1.098842	108.7447	124.5636	5.2256	3.676549	2.44953
H	1.098357	110.6315	-59.3202	5.293023	0.631032	2.033228
H	1.100294	110.3457	42.74829	6.031357	2.923814	0.150194
H	1.091013	110.5877	-114.545	3.790276	3.867322	0.37727
H	1.09486	160.8012	-141.228	2.807297	3.474595	2.809044
H	1.0959	107.7743	-121.301	4.82823	2.481235	3.663437
H	1.096778	107.7274	-175.193	6.666706	1.683446	2.338919
H	1.09634	110.1392	127.0275	2.064843	3.594143	0.604865
H	1.09804	109.6881	159.8247	6.761073	1.324577	0.030954
C	1.501595	111.9877	10.59348	3.020508	1.938367	-0.20349
O	1.432365	112.745	-81.7065	4.770882	1.347152	-0.40293
O	1.196094	128.8926	59.6646	2.290125	0.991646	-0.17347
C	3.595183	110.7065	-146.712	-4.53127	2.01244	1.006695
H	1.099597	87.42334	165.222	-5.54182	2.393194	1.213892
H	1.100019	73.52288	56.15768	-4.25747	2.337705	-0.00784
C	1.521603	159.3866	-46.9678	-3.54727	2.580434	2.018823
H	1.098082	108.6997	-82.3691	-3.82655	2.224468	3.019363
H	1.099117	108.723	33.46589	-2.54831	2.17453	1.805813
C	1.534003	113.0349	155.6744	-3.4977	4.113623	2.012605
H	1.098241	109.4092	-57.3561	-3.23933	4.466336	1.005147
H	1.097371	109.3201	58.41032	-4.49843	4.5101	2.226091
C	1.531232	112.9057	-179.627	-2.50001	4.681767	3.025771

H	1.096397	111.287	60.20518	-1.4808	4.335127	2.818079
H	1.093662	111.2506	-179.674	-2.49076	5.775145	3.002624
H	1.0954	111.2247	-59.7655	-2.75291	4.374643	4.046368
O	1.433867	52.37348	-76.8556	-4.51887	0.580897	1.087344
H	0.972524	107.9988	-6.25086	-5.09853	0.235426	0.387023
C	2.828489	87.79635	-163.666	3.354104	3.72011	-2.37477
H	1.095791	133.1896	133.8971	4.004279	3.714074	-3.25681
H	1.096928	87.25406	22.46156	3.781571	4.431143	-1.65717
C	1.521903	104.0821	-88.6732	1.933151	4.110604	-2.75503
H	1.097074	108.6155	-86.3958	1.540588	3.363963	-3.45645
H	1.098472	109.4317	29.6535	1.29255	4.071948	-1.86352
C	1.535589	112.2868	151.6787	1.861962	5.51043	-3.38228
H	1.097864	109.4435	-58.1683	2.268725	6.247691	-2.6778
H	1.097616	109.2754	57.68151	2.509921	5.544151	-4.26759
C	1.531279	112.8385	179.6758	0.438308	5.913097	-3.77711
H	1.095667	111.3365	60.35775	-0.22741	5.928566	-2.90701
H	1.093325	110.9954	-179.767	0.423008	6.911045	-4.22345
H	1.095494	111.2704	-59.9204	0.016559	5.215599	-4.50904
O	1.447462	30.24942	163.6154	3.398391	2.374454	-1.84336
H	1.148485	119.3152	-94.2346	4.393085	1.958675	-1.44747

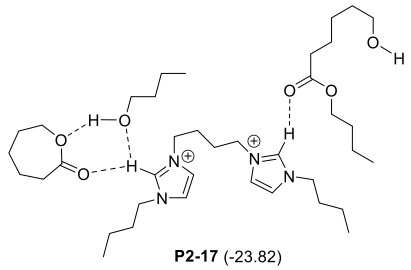

P2-17 (-23.82)

Atom	Bond	Angle	Dihedral	X	Y	Z
C				3.627398	-2.21793	-1.2686
H	1.084471			3.660646	-1.19005	-0.92446
C	2.210409	163.4664		4.105413	-4.34285	-1.64558
H	1.078531	157.1063	-0.07201	4.671405	-5.25917	-1.58866
C	1.362705	72.14774	-178.344	2.949744	-4.05109	-2.30608
H	1.078262	130.5737	178.2769	2.330357	-4.66481	-2.94039
N	1.342152	72.28891	0.127586	2.664108	-2.71945	-2.05722
N	1.338909	108.6748	-0.0198	4.515012	-3.18579	-1.00768
C	1.481574	125.6686	-178.699	5.715399	-3.04883	-0.15013
H	1.093752	106.443	-141.143	6.51134	-3.61832	-0.63845
H	1.094505	107.1365	-25.9664	6.002184	-1.99265	-0.16308

C	1.531755	112.3886	97.21759	5.475038	-3.5556	1.275239
H	1.097601	109.5488	55.93194	5.129396	-4.59681	1.241447
H	1.097678	109.1009	-60.6459	4.668919	-2.96858	1.734014
C	1.535465	111.7334	177.6136	6.744508	-3.46128	2.133844
H	1.097558	109.2668	-58.0927	7.546204	-4.04211	1.65998
H	1.097692	109.2786	57.81028	7.090702	-2.41984	2.155695
C	1.531405	112.5848	179.9431	6.525539	-3.96207	3.564389
H	1.095025	111.3009	60.12135	6.213517	-5.01165	3.575034
H	1.09304	110.8761	179.882	7.445626	-3.88452	4.149335
H	1.095369	111.3428	-60.2552	5.754687	-3.3773	4.077871
C	1.481499	125.4619	-176.531	1.53489	-1.9502	-2.62989
H	1.092816	106.4803	6.876685	1.719761	-0.9009	-2.38694
H	1.093246	106.6323	122.771	1.590706	-2.07326	-3.71476
C	1.533378	113.5131	-115.915	0.16275	-2.40636	-2.1196
H	1.09644	106.1935	-168.204	-0.57991	-1.92103	-2.76387
H	1.097759	109.8985	-54.0182	0.043746	-3.48556	-2.28158
C	1.536322	114.7414	71.00081	-0.12873	-2.05103	-0.65363
H	1.095816	109.684	56.99004	-0.03782	-0.96832	-0.51117
H	1.09721	110.3551	-60.3998	0.610448	-2.52091	0.007202
C	1.535122	110.3529	178.1749	-1.53986	-2.51215	-0.26289
H	1.091732	111.907	-57.7567	-2.3049	-2.07068	-0.90452
H	1.094485	110.7429	61.99724	-1.62979	-3.5996	-0.34808
C	2.50603	116.9829	-149.99	-2.72855	-1.17374	1.490916
C	2.215064	64.93894	-87.9209	-1.44708	-2.80639	2.264731
H	1.086884	98.49342	93.03465	-3.26663	-0.49318	0.836225
H	1.078028	157.2638	-2.18828	-0.77883	-3.65122	2.22169
C	1.361908	72.00554	-179.497	-2.02714	-2.18621	3.329487
H	1.078223	130.7485	178.9971	-1.95415	-2.38689	4.386353
N	1.342904	28.6001	-87.3035	-1.89597	-2.16157	1.12434
N	1.338838	137.0348	-88.6716	-2.8222	-1.17256	2.826474
C	1.482921	125.4921	-179.295	-3.64359	-0.23614	3.631138
H	1.094035	106.7819	128.4815	-4.25101	-0.84768	4.304916
H	1.092225	106.2826	12.47336	-4.30466	0.273969	2.927054
C	1.531015	112.3284	-109.412	-2.78766	0.763914	4.41299
H	1.097819	109.9563	-56.4213	-2.0757	0.227335	5.053606
H	1.097116	108.9644	60.39499	-2.19488	1.356105	3.704764
C	1.5356	111.5465	-178.049	-3.65322	1.692983	5.276535
H	1.09768	109.3243	58.40936	-4.24952	1.090013	5.973498
H	1.097532	109.2566	-57.4573	-4.3711	2.219248	4.634453
C	1.53134	112.5645	-179.563	-2.82045	2.710737	6.061183
H	1.093159	110.906	-179.64	-3.46194	3.352888	6.670382

H	1.095199	111.2943	-59.8468	-2.11456	2.212641	6.734293
H	1.095304	111.3191	60.46513	-2.2445	3.356413	5.389567
C	6.095722	139.1379	-34.8377	-7.07095	-3.05517	-2.76688
C	1.533112	139.5026	-114.778	-8.47921	-2.46679	-2.62186
C	1.533831	114.9234	-3.14814	-8.64381	-1.45854	-1.47775
C	1.547696	49.95996	-33.6801	-5.96306	-2.00409	-3.0182
C	1.523935	114.7645	59.74536	-7.74551	-0.23129	-1.57424
H	1.097332	60.18362	158.1327	-6.81137	-3.6511	-1.88279
H	1.09834	108.8338	119.1396	-8.75908	-1.98007	-3.56585
H	1.097291	111.1159	-63.7221	-8.47659	-1.93522	-0.50366
H	1.095261	112.1746	43.81968	-7.71312	0.178502	-2.58943
H	1.097594	109.7555	-179.322	-6.30507	-1.27582	-3.76477
H	1.094477	111.629	61.29224	-7.06373	-3.7471	-3.61486
H	1.094977	108.8907	-125.623	-9.19243	-3.28529	-2.47917
H	1.096319	108.9058	179.5763	-9.67884	-1.09722	-1.46885
H	1.091278	110.3427	62.55609	-5.0682	-2.48422	-3.41769
H	1.090711	110.8865	164.8084	-8.08883	0.55574	-0.90166
C	1.512238	114.0223	-55.925	-5.50919	-1.27798	-1.77175
O	1.353542	119.4696	-65.2046	-6.37746	-0.47182	-1.11732
O	1.218356	123.4549	113.7729	-4.39177	-1.38904	-1.29906
C	4.699623	116.3165	175.665	4.327144	1.891227	0.90203
C	1.542629	124.886	11.97669	5.612905	2.280915	1.660098
C	1.541096	116.7793	0.798776	6.748719	1.239557	1.681918
C	1.535999	77.46477	122.829	4.325251	2.293351	-0.5804
C	1.522919	118.0826	71.27049	7.513167	0.985323	0.38953
H	1.093709	31.8644	-58.2996	4.159217	0.813671	0.984927
H	1.098623	110.1067	126.3146	6.000721	3.235928	1.279939
H	1.098708	109.6891	-53.6547	6.363943	0.27985	2.053495
H	1.099229	110.1448	48.58232	7.820805	1.939049	-0.06221
H	1.098142	109.3047	-71.9507	5.24982	1.937594	-1.05424
H	1.096249	117.8931	-133.238	3.466658	2.369608	1.384188
H	1.095578	107.9049	-119.202	5.343141	2.472678	2.704486
H	1.096811	107.7369	-168.532	7.491937	1.571106	2.417238
H	1.092611	110.1347	170.3207	4.308287	3.381741	-0.67484
H	1.098105	109.2424	166.5762	8.424229	0.414941	0.614175
C	1.515532	112.977	45.30349	3.194833	1.657505	-1.36442
O	1.44395	110.3779	-73.0283	6.696388	0.240597	-0.53958
O	1.228346	121.9913	-25.9851	2.682419	0.595	-1.02186
C	3.573073	110.7936	-144.952	-4.68703	2.35619	0.265312
H	1.099499	84.46778	162.154	-5.69374	2.793985	0.326747
H	1.100086	75.30646	52.46283	-4.39507	2.347657	-0.79529

C	1.521593	161.3704	-56.9429	-3.70899	3.200314	1.069134
H	1.098039	108.6773	-75.3328	-4.01173	3.180032	2.124418
H	1.099075	108.8434	40.60485	-2.71347	2.73766	1.015659
C	1.534238	112.9504	162.7698	-3.63238	4.653321	0.582524
H	1.098181	109.3799	-57.9563	-3.34135	4.668165	-0.47629
H	1.097512	109.3058	57.81114	-4.63152	5.104999	0.629905
C	1.531299	112.9139	179.8089	-2.65135	5.50323	1.39499
H	1.096142	111.287	60.04649	-1.63406	5.098735	1.339804
H	1.093645	111.2159	-179.876	-2.62024	6.532509	1.026648
H	1.095436	111.2461	-60.0022	-2.93782	5.538955	2.4517
O	1.434644	52.98758	-77.6296	-4.70095	1.019144	0.785234
H	0.972646	107.848	-5.71995	-5.31774	0.495703	0.245212
C	2.462259	91.35026	156.0108	3.27241	3.454575	-3.04586
H	1.092496	126.0801	139.7062	3.169013	3.329695	-4.12626
H	1.09316	87.50653	29.4141	4.337694	3.55346	-2.82138
C	1.522843	109.8787	-83.3961	2.466753	4.652457	-2.56104
H	1.096673	108.5078	-82.1552	1.407667	4.471712	-2.78095
H	1.098148	109.7802	34.00139	2.550423	4.739414	-1.46955
C	1.536091	112.0006	156.1411	2.926507	5.96069	-3.2219
H	1.098013	109.468	-58.6151	3.989874	6.1266	-3.00428
H	1.097623	109.2867	57.28733	2.849122	5.862706	-4.3124
C	1.531415	112.6992	179.2813	2.114821	7.173857	-2.75862
H	1.095376	111.34	60.1429	2.200152	7.3209	-1.67652
H	1.093288	110.9655	-179.961	2.464591	8.087822	-3.24607
H	1.095229	111.2409	-60.1073	1.052554	7.055216	-2.99745
O	1.33639	120.8376	157.3596	2.75813	2.204703	-2.50275
H	0.967025	107.0518	179.293	7.221225	0.116596	-1.34227

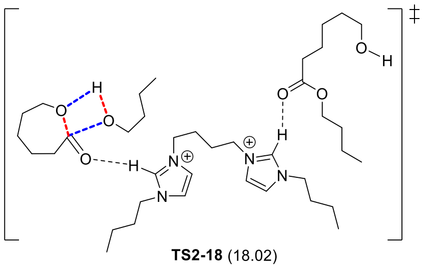

TS2-18 (18.02)

Atom	Bond	Angle	Dihedral	X	Y	Z
C				3.337354	-1.64726	-1.72307
H	1.084676			3.360999	-0.81499	-1.02788
C	2.210517	163.4895		3.864086	-3.44802	-2.89192
H	1.078553	157.1063	-0.16442	4.470819	-4.27704	-3.22036

C	1.362528	72.15292	-178.484	2.636934	-3.02527	-3.30648
H	1.078202	130.5338	178.2715	1.984055	-3.41278	-4.07206
N	1.342108	72.27223	0.168878	2.321616	-1.90096	-2.56281
N	1.339311	108.6479	-0.07876	4.286775	-2.5738	-1.90711
C	1.48148	125.8294	-178.843	5.563047	-2.66782	-1.16074
H	1.093714	106.3742	-141.667	6.323172	-2.97032	-1.88663
H	1.094425	107.1771	-26.4873	5.810813	-1.65916	-0.8158
C	1.531943	112.5212	96.73123	5.491145	-3.66325	0.001508
H	1.097614	109.6311	55.32322	5.170821	-4.64422	-0.37243
H	1.097714	109.1415	-61.3094	4.725293	-3.33012	0.713879
C	1.535484	111.6255	176.9697	6.844482	-3.80015	0.713861
H	1.097523	109.243	-58.5821	7.601701	-4.13073	-0.00856
H	1.09773	109.2688	57.26661	7.168604	-2.81318	1.068613
C	1.531383	112.6273	179.4052	6.794841	-4.77978	1.889871
H	1.095003	111.3322	60.22229	6.510356	-5.78433	1.559765
H	1.09307	110.8799	179.9732	7.771331	-4.85569	2.375162
H	1.095348	111.3299	-60.1651	6.071444	-4.45752	2.646596
C	1.481843	125.605	-176.284	1.109783	-1.06439	-2.72857
H	1.092825	106.552	4.471815	1.25336	-0.18075	-2.10181
H	1.093341	106.5768	120.2818	1.088929	-0.74948	-3.77537
C	1.532936	113.4339	-118.513	-0.18966	-1.79685	-2.37521
H	1.095553	106.7758	-165.802	-1.01193	-1.19109	-2.77165
H	1.097835	109.7827	-50.9897	-0.23106	-2.75704	-2.90584
C	1.536531	114.6417	74.17087	-0.42084	-2.00133	-0.87
H	1.095182	109.1194	61.14824	-0.47974	-1.02362	-0.38005
H	1.097076	111.0517	-56.7155	0.41698	-2.54198	-0.41249
C	1.535055	109.8511	-178.921	-1.73285	-2.76525	-0.64317
H	1.090201	109.6957	-49.4378	-2.53393	-2.29788	-1.21621
H	1.093879	111.3953	70.97564	-1.64432	-3.81087	-0.95206
C	2.498183	117.7247	-139.43	-3.19195	-2.03322	1.24788
C	2.214312	65.31785	-86.5764	-1.6198	-3.49024	1.803407
H	1.091363	97.46324	94.85133	-3.84662	-1.37427	0.674937
H	1.078064	157.2963	-2.05438	-0.79147	-4.16423	1.655657
C	1.362673	72.07181	-179.852	-2.33007	-3.19071	2.927097
H	1.078213	130.7144	179.1799	-2.23201	-3.55196	3.938251
N	1.342033	28.99888	-86.4179	-2.17152	-2.75934	0.76568
N	1.339243	137.4163	-86.8427	-3.30567	-2.28165	2.558956
C	1.480268	125.1018	179.8567	-4.30945	-1.66839	3.45758
H	1.093944	107.2928	124.6662	-4.85259	-2.48432	3.943363
H	1.092411	106.2818	9.441418	-5.01173	-1.1345	2.813277
C	1.530886	112.7864	-112.485	-3.68427	-0.72632	4.489691

H	1.097363	109.6659	-58.4727	-2.95326	-1.27582	5.096226
H	1.097205	109.139	58.15622	-3.13132	0.0641	3.966861
C	1.536054	111.5951	-179.957	-4.74879	-0.10667	5.407459
H	1.097467	109.3128	57.91877	-5.3025	-0.90774	5.913543
H	1.097732	109.2916	-58.0246	-5.48208	0.436397	4.797236
C	1.531309	112.5188	179.9009	-4.14565	0.838364	6.45055
H	1.092942	110.8074	-179.954	-4.92595	1.262082	7.087815
H	1.094997	111.3052	-60.2096	-3.43589	0.31425	7.099062
H	1.095266	111.3628	60.19165	-3.61531	1.670667	5.97555
C	5.112553	119.9392	-46.7177	-5.96927	-1.35627	-3.1343
C	1.535227	107.4653	-156.333	-7.17076	-1.84688	-2.31416
C	1.5364	117.0039	29.08108	-6.978	-1.961	-0.79418
C	1.541868	85.23108	-41.9344	-5.52023	0.084841	-2.81977
C	1.530198	115.853	63.85376	-6.70606	-0.64868	-0.05568
H	1.097092	29.88453	102.3204	-5.1161	-2.03602	-3.01749
H	1.098345	108.6015	152.3405	-8.02315	-1.18233	-2.50953
H	1.098976	110.5769	-60.1779	-6.1704	-2.66835	-0.55928
H	1.099644	110.3463	43.0164	-7.36946	0.144797	-0.42918
H	1.091659	111.6484	-142.293	-6.36955	0.728941	-2.58416
H	1.094681	132.0535	67.59798	-6.24419	-1.39446	-4.19321
H	1.095947	108.0713	-93.3772	-7.46577	-2.83007	-2.69812
H	1.096835	107.7978	-175.778	-7.89352	-2.39264	-0.3716
H	1.096924	110.3168	99.23363	-5.02126	0.526981	-3.69085
H	1.098436	109.4558	160.0143	-6.93701	-0.77439	1.010826
C	1.502613	113.3732	-17.1289	-4.49118	0.16363	-1.72766
O	1.434784	113.2118	-81.4089	-5.33485	-0.2326	-0.12826
O	1.204266	129.0403	62.72419	-3.37529	-0.28851	-1.70285
C	4.654118	114.9218	173.5359	4.094777	1.257212	1.833779
C	1.542672	124.9125	12.6028	5.431653	1.396117	2.590948
C	1.540496	117.0067	2.16587	6.610233	0.538363	2.092617
C	1.537159	78.16153	123.846	3.935407	2.233895	0.657535
C	1.523117	118.1643	71.46272	7.260373	0.910926	0.76657
H	1.093985	31.20464	-56.9918	3.975044	0.231603	1.472401
H	1.098576	109.977	127.7332	5.736619	2.451008	2.623656
H	1.09867	109.7101	-53.6058	6.307944	-0.51699	2.048772
H	1.099255	110.2048	48.38009	7.477677	1.988153	0.739687
H	1.098012	109.0485	-68.6449	4.794834	2.125529	-0.0172
H	1.09646	117.6735	-131.848	3.268966	1.4314	2.533721
H	1.095621	107.8118	-117.896	5.256774	1.112275	3.634613
H	1.096824	107.6518	-168.388	7.403626	0.592315	2.848024
H	1.091657	109.8358	173.7421	3.934402	3.261257	1.026644

H	1.098035	109.2157	166.36	8.215182	0.377412	0.669704
C	1.515882	112.6857	48.66614	2.707847	1.941968	-0.18257
O	1.443749	110.3636	-73.2769	6.39671	0.554451	-0.33408
O	1.227676	121.7596	-32.5602	2.289743	0.798508	-0.34029
C	2.65998	125.8192	-178.45	-3.4217	2.396321	-0.75463
H	1.096146	77.9603	-154.74	-2.64278	1.656236	-0.53763
H	1.094495	132.8656	100.5602	-3.61549	2.982284	0.149259
C	1.521091	108.2844	-45.8865	-3.03157	3.287572	-1.9239
H	1.097668	108.8665	-23.7418	-2.88399	2.661679	-2.81348
H	1.096173	108.8336	92.35539	-3.86477	3.964196	-2.1465
C	1.53513	112.5018	-145.467	-1.75735	4.097306	-1.64584
H	1.098528	109.1694	-58.0236	-1.90895	4.711284	-0.74761
H	1.098805	109.6418	58.11954	-0.92796	3.413825	-1.41708
C	1.531934	112.469	-179.624	-1.37027	5.001488	-2.82034
H	1.094694	111.2608	59.63161	-2.16638	5.717032	-3.04961
H	1.094441	111.3258	179.4277	-0.46617	5.576974	-2.59848
H	1.095361	111.3047	-60.3671	-1.17941	4.416747	-3.72668
O	1.448838	34.65856	53.00236	-4.65525	1.698162	-1.05471
H	1.121821	117.0835	89.96754	-5.08556	1.050852	-0.24582
C	2.461636	91.39118	150.0947	2.477624	4.318893	-0.77996
H	1.092422	126.3489	143.5118	2.150193	4.715507	-1.74374
H	1.093247	87.56577	33.22331	3.568362	4.384883	-0.74641
C	1.523045	109.7142	-79.5539	1.818882	5.075231	0.366197
H	1.096649	108.5986	-82.4698	0.732749	4.941521	0.294959
H	1.097912	109.9189	33.77798	2.125818	4.640507	1.326518
C	1.536378	111.9156	155.8613	2.167778	6.571265	0.341505
H	1.097906	109.4493	-58.2794	3.257426	6.693497	0.397394
H	1.097713	109.3015	57.58046	1.861566	6.9994	-0.62178
C	1.531427	112.7007	179.6275	1.506943	7.351145	1.481835
H	1.095356	111.3236	60.29191	1.823723	6.972181	2.459506
H	1.093229	110.9893	-179.804	1.770687	8.411078	1.435672
H	1.095325	111.2593	-59.9293	0.415184	7.277764	1.432698
O	1.33819	121.001	149.9767	2.063577	2.922833	-0.82566
H	0.967075	107.1316	179.9275	6.851775	0.810992	-1.14792

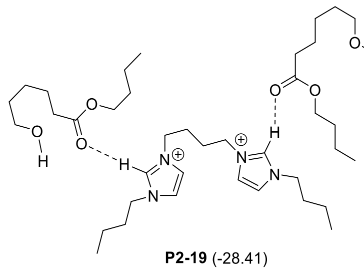

P2-19 (-28.41)

Atom	Bond	Angle	Dihedral	X	Y	Z
C				4.096637	-1.10294	-1.84638
H	1.084561			4.004837	-0.34142	-1.07961
C	2.210402	163.424		4.828564	-2.77034	-3.09935
H	1.078551	157.1117	0.05357	5.499597	-3.55078	-3.42168
C	1.362889	72.14617	-178.229	3.651474	-2.33425	-3.63015
H	1.078305	130.5546	178.287	3.115264	-2.65859	-4.50766
N	1.342149	72.29809	0.147548	3.206338	-1.29377	-2.83244
N	1.338925	108.6822	-0.05179	5.091579	-1.98739	-1.98977
C	1.481781	125.5572	-178.129	6.253291	-2.13227	-1.08141
H	1.093649	106.4962	-142.328	7.116996	-2.35359	-1.71473
H	1.094581	107.1377	-27.0296	6.419975	-1.15716	-0.61291
C	1.531647	112.2423	95.99143	6.040118	-3.23375	-0.0387
H	1.097656	109.552	56.29283	5.818068	-4.1816	-0.54578
H	1.097739	109.0283	-60.2593	5.160583	-2.98552	0.569434
C	1.535682	111.8595	178.0718	7.267556	-3.40856	0.867476
H	1.09748	109.2964	-58.4456	8.142411	-3.65018	0.25046
H	1.097719	109.2776	57.49282	7.492699	-2.45413	1.360795
C	1.531354	112.5299	179.587	7.067076	-4.49987	1.922883
H	1.095031	111.3051	60.12253	6.875225	-5.47256	1.457943
H	1.093037	110.8857	179.8835	7.955968	-4.60294	2.55056
H	1.095368	111.318	-60.2646	6.221363	-4.26838	2.579398
C	1.48148	125.3848	-176.861	1.994585	-0.47373	-3.06474
H	1.092926	106.4171	7.107528	2.052245	0.366745	-2.36849
H	1.093229	106.6973	122.9958	2.06708	-0.08822	-4.08517
C	1.533151	113.4973	-115.672	0.686107	-1.25345	-2.89017
H	1.096376	106.1651	-169.794	-0.11078	-0.60018	-3.26468
H	1.097848	109.8901	-55.5294	0.68718	-2.13589	-3.54329
C	1.536182	114.7542	69.46335	0.368204	-1.66088	-1.44353
H	1.095536	109.7633	57.19674	0.334963	-0.77014	-0.8066
H	1.097264	110.1684	-60.3709	1.162292	-2.30747	-1.0494
C	1.53447	110.2204	178.1693	-0.9795	-2.39201	-1.38241
H	1.092184	110.9143	-59.4182	-1.78547	-1.7557	-1.75444

H	1.094943	110.8417	60.98718	-0.95493	-3.30536	-1.9858
C	2.499861	122.3027	-151.001	-2.44081	-2.33263	0.644993
C	2.213654	65.30495	-77.4391	-0.70769	-3.70171	0.793919
H	1.082321	95.95377	103.4478	-3.15387	-1.62139	0.248637
H	1.077719	157.429	-2.02736	0.180496	-4.22129	0.473538
C	1.362203	71.92881	-179.159	-1.41597	-3.78889	1.954235
H	1.078333	130.765	179.2791	-1.25372	-4.38988	2.834741
N	1.342085	28.93493	-76.0427	-1.36057	-2.78358	-0.01145
N	1.337669	137.527	-78.813	-2.49415	-2.9301	1.840629
C	1.481608	125.4439	-179.849	-3.52257	-2.68481	2.87858
H	1.093885	106.6402	135.7266	-3.81985	-3.66492	3.262783
H	1.093002	106.1474	20.56318	-4.37976	-2.24791	2.35992
C	1.531438	112.4908	-102.24	-3.01341	-1.78199	4.00595
H	1.097798	109.7928	-57.1277	-2.11808	-2.22496	4.461296
H	1.097411	109.1572	59.56438	-2.7085	-0.81599	3.583844
C	1.535506	111.4896	-178.791	-4.08666	-1.56735	5.082903
H	1.097623	109.2877	58.14167	-4.39224	-2.54014	5.489187
H	1.097591	109.2525	-57.7294	-4.98199	-1.13277	4.620045
C	1.531394	112.6009	-179.843	-3.6051	-0.66405	6.221901
H	1.093155	110.8998	-179.928	-4.3889	-0.53118	6.972229
H	1.09514	111.2988	-60.1422	-2.73204	-1.0913	6.726439
H	1.095272	111.3253	60.18818	-3.32642	0.328622	5.852357
C	4.981412	80.58176	134.3527	-5.87483	-1.47569	-2.86038
C	1.550255	102.0283	175.0064	-7.18617	-1.47047	-2.03356
C	1.545168	114.247	-46.357	-7.31095	-2.64242	-1.0343
C	1.552254	86.17746	-70.2221	-5.20222	-0.0874	-3.03285
C	1.530952	113.8626	97.95125	-6.91509	-2.27887	0.399206
H	1.094331	29.99382	65.50809	-5.13998	-2.14038	-2.3959
H	1.0976	110.2953	76.05987	-7.28196	-0.52895	-1.4776
H	1.09655	109.8753	-25.4521	-6.70875	-3.49166	-1.37864
H	1.098125	109.9068	68.83998	-7.65232	-1.58615	0.826424
H	1.091514	110.8886	-150.379	-5.92868	0.659218	-3.35877
H	1.094277	134.6318	41.26003	-6.07232	-1.87941	-3.8581
H	1.093976	108.7821	-169.227	-8.03651	-1.48166	-2.72172
H	1.09639	110.2967	-142.068	-8.34658	-3.00021	-0.99515
H	1.093383	109.2816	90.83676	-4.42461	-0.15556	-3.79846
H	1.094317	109.9695	-173.408	-6.91402	-3.17617	1.025609
C	1.515152	108.693	-25.206	-4.50604	0.279129	-1.73798
O	1.441732	113.806	-54.318	-5.59672	-1.70625	0.511425
O	1.22169	121.4216	73.10067	-3.47171	-0.27185	-1.39286
C	4.716706	116.565	176.0962	4.282188	1.682715	1.955332

C	1.542803	124.9703	11.77312	5.504869	1.830327	2.884581
C	1.540827	116.6795	-0.12516	6.749189	0.994126	2.528794
C	1.535906	77.20018	122.4185	4.249133	2.690232	0.796529
C	1.523061	118.0842	71.4456	7.557014	1.396969	1.30207
H	1.093591	32.07824	-58.5786	4.243263	0.66704	1.55181
H	1.098521	110.1351	125.3625	5.788304	2.888092	2.971447
H	1.0987	109.6882	-53.5179	6.468473	-0.06347	2.429633
H	1.09926	110.1409	48.91322	7.768126	2.475516	1.325332
H	1.098041	109.3457	-72.4618	5.21727	2.680733	0.278538
H	1.096136	117.8935	-133.794	3.365333	1.811177	2.542179
H	1.095558	107.9408	-120.111	5.201029	1.527716	3.892725
H	1.096877	107.774	-168.391	7.440167	1.043514	3.379234
H	1.092684	110.1688	169.7966	4.095276	3.70148	1.180799
H	1.098109	109.2357	166.8694	8.520351	0.870019	1.314233
C	1.51567	112.922	44.79771	3.218536	2.342333	-0.25898
O	1.44427	110.4331	-72.6973	6.840288	1.061483	0.093903
O	1.228239	122.0085	-25.4501	2.833569	1.189815	-0.43806
C	2.466363	92.86579	-111.739	-6.08506	2.113519	-1.26399
H	1.094258	97.34047	40.9487	-6.99752	1.596934	-1.57698
H	1.094712	90.77166	-68.0513	-5.69695	2.686576	-2.11215
C	1.519525	134.5924	170.4698	-6.34859	3.011718	-0.06702
H	1.0975	108.9018	-63.2809	-6.68545	2.392932	0.774489
H	1.096321	108.8994	52.79231	-5.40565	3.478424	0.241163
C	1.53573	112.0941	174.9592	-7.39784	4.090266	-0.37406
H	1.097851	109.4296	-58.3458	-7.05774	4.699912	-1.22138
H	1.097847	109.5037	57.70119	-8.33197	3.611745	-0.69605
C	1.531483	112.5327	179.6955	-7.67669	4.996923	0.828289
H	1.095214	111.2635	59.89947	-6.76879	5.516176	1.153217
H	1.093234	110.9594	179.8097	-8.42347	5.756023	0.580782
H	1.095238	111.2889	-60.3098	-8.0562	4.422388	1.68001
O	1.349013	120.858	-100.458	-5.07587	1.134355	-0.86407
H	0.967296	108.4913	87.23838	-5.66479	-0.75077	0.376964
C	2.461923	91.31792	156.5986	3.108091	4.685931	-1.00491
H	1.092535	126.0224	139.3916	3.045086	5.028312	-2.04049
H	1.093167	87.44639	29.16886	4.14784	4.786338	-0.68264
C	1.522884	110.0239	-83.6291	2.150679	5.470852	-0.1181
H	1.096661	108.503	-81.9298	1.126745	5.29235	-0.4679
H	1.098079	109.8341	34.24156	2.201099	5.092115	0.911367
C	1.536183	111.9608	156.3952	2.457705	6.975974	-0.13227
H	1.098	109.4635	-58.6657	3.487675	7.142506	0.20983
H	1.097633	109.2784	57.22551	2.415143	7.344799	-1.16521

C	1.531406	112.6992	179.2393	1.492731	7.784186	0.73998
H	1.095336	111.3264	59.95261	1.535306	7.461193	1.785745
H	1.093302	110.9744	179.8571	1.737923	8.849257	0.711448
H	1.095239	111.2402	-60.2699	0.458684	7.671489	0.39706
O	1.336396	120.8115	157.9522	2.739412	3.279123	-1.08288
H	0.967038	107.01	178.6839	7.384835	1.355833	-0.64906

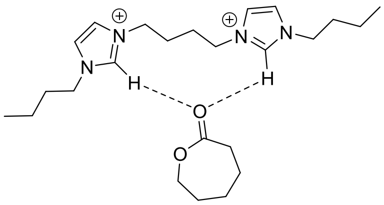

COM4 (-22.94)

Atom	Bond	Angle	Dihedral	X	Y	Z
C				-3.12222	-0.59948	-0.20743
H	1.08597			-2.14779	-0.89236	0.172107
C	2.211725	162.392		-5.12238	0.326796	-0.38934
H	1.078508	157.2102	-4.01	-5.98493	0.898198	-0.0849
C	1.362716	72.16417	178.0786	-4.87839	-0.36812	-1.53588
H	1.078779	130.6756	178.7301	-5.48995	-0.51581	-2.4122
N	1.343963	72.21697	-0.03296	-3.62539	-0.94129	-1.40585
N	1.338643	108.5397	0.089736	-4.01634	0.169476	0.425992
C	1.482412	125.775	-179.979	-3.85064	0.758588	1.776191
H	1.093701	106.9793	-125.559	-4.71626	0.448456	2.368396
H	1.092691	106.5458	-10.6115	-2.96505	0.290927	2.213211
C	1.531017	112.8428	111.7236	-3.71475	2.283279	1.746815
H	1.097046	109.5978	58.24828	-4.59996	2.722489	1.270353
H	1.097325	109.2217	-58.4102	-2.85221	2.557211	1.12624
C	1.536321	111.5075	179.6508	-3.55349	2.861739	3.160909
H	1.097275	109.2721	-57.7275	-4.41501	2.56697	3.7732
H	1.09805	109.3262	58.23005	-2.67007	2.416284	3.637192
C	1.531488	112.5125	-179.653	-3.424	4.387742	3.158973
H	1.094669	111.3472	60.40508	-4.31051	4.862328	2.726327
H	1.092833	110.71	-179.933	-3.30982	4.767202	4.17743
H	1.095277	111.4374	-60.0801	-2.5524	4.714797	2.58194
C	1.478326	125.9625	-179.045	-2.96561	-1.80115	-2.41122
H	1.092886	107.1613	35.27405	-2.40885	-2.56681	-1.86515
H	1.091678	106.4343	150.0188	-3.76446	-2.3049	-2.95878
C	1.534624	114.1552	-87.9032	-2.04739	-1.04372	-3.37985

H	1.096569	106.0792	-171.331	-1.75801	-1.7666	-4.15197
H	1.096171	109.807	-56.6161	-2.61373	-0.25486	-3.88833
C	1.535692	113.327	68.05267	-0.79249	-0.47042	-2.70539
H	1.094975	109.0986	49.66773	-0.3115	-1.25758	-2.11547
H	1.096082	110.2556	-67.2294	-1.06861	0.325298	-2.00398
C	1.527518	111.7474	171.2042	0.196416	0.084831	-3.72866
H	1.094653	110.7352	-59.6618	0.515686	-0.69834	-4.42363
H	1.093894	110.9915	60.09133	-0.25691	0.88918	-4.31528
C	2.531345	88.29979	-175.085	1.810242	0.511087	-1.82561
C	2.214945	64.0496	-170.626	2.376904	1.368852	-3.78753
H	1.085057	98.77493	9.486402	1.278979	-0.01254	-1.03763
H	1.078765	157.2813	0.197263	2.283505	1.598869	-4.83734
C	1.361796	71.99645	-178.943	3.360259	1.672286	-2.89566
H	1.07855	130.5901	179.9229	4.280049	2.220533	-3.02482
N	1.344066	27.69772	-169.064	1.414645	0.645172	-3.10313
N	1.338722	136.1231	-172.138	2.989151	1.128471	-1.68005
C	1.481661	125.449	179.6412	3.747118	1.245581	-0.41234
H	1.093871	106.9158	129.2589	4.768608	0.91424	-0.62049
H	1.092539	106.3074	14.11693	3.296309	0.532277	0.281641
C	1.53131	112.5834	-108.363	3.731425	2.668668	0.152891
H	1.097087	109.5643	-57.9933	4.14896	3.362554	-0.58724
H	1.097217	109.2087	58.55842	2.692635	2.977612	0.324221
C	1.536028	111.6789	-179.464	4.533575	2.769329	1.458955
H	1.097558	109.292	57.46946	5.564677	2.439862	1.277532
H	1.098213	109.3073	-58.5318	4.112781	2.074469	2.19799
C	1.531524	112.5098	179.3888	4.542911	4.18902	2.033372
H	1.092898	110.7804	-179.998	5.120333	4.228414	2.960442
H	1.094685	111.2864	-60.3102	4.99346	4.898544	1.332005
H	1.095104	111.4119	60.0968	3.528777	4.536392	2.25724
C	5.604659	129.817	-146.85	0.527805	-4.31223	1.867423
C	1.533051	150.9573	-95.7368	1.932178	-4.67915	2.360724
C	1.533727	114.8554	-26.0945	3.065005	-3.8301	1.770701
C	1.549379	43.06089	-48.9636	0.110255	-2.84946	2.161598
C	1.523287	114.7309	59.32829	2.951779	-2.33626	2.046417
H	1.09747	72.5139	163.4045	0.435969	-4.50619	0.79114
H	1.097971	108.8386	96.24721	1.957077	-4.59387	3.455094
H	1.0973	111.201	-64.3523	3.150034	-3.97981	0.686992
H	1.09492	112.3246	43.92454	2.672008	-2.1248	3.083655
H	1.097346	109.6642	159.0375	0.395521	-2.58316	3.187208
H	1.094201	97.29608	58.59677	-0.20095	-4.96354	2.359336
H	1.094597	108.835	-148.561	2.1221	-5.7331	2.134313

H	1.096149	108.9085	178.995	4.01766	-4.16101	2.200221
H	1.091592	110.1856	41.25046	-0.97408	-2.74575	2.090652
H	1.090532	110.9668	165.0537	3.894602	-1.82872	1.839656
C	1.511677	113.7437	-77.851	0.694079	-1.84384	1.195659
O	1.336937	120.0811	-65.2103	2.015201	-1.64297	1.154581
O	1.230767	122.8168	113.4737	0.004207	-1.19821	0.406971

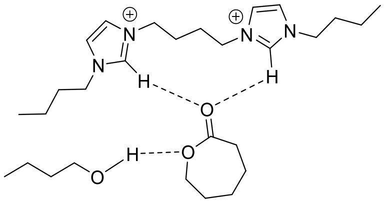

COM5 (-13.74)

Atom	Bond	Angle	Dihedral	X	Y	Z
C				-0.49561	2.533362	-1.27256
H	1.084527			-0.36986	1.870845	-0.42317
C	2.211614	161.9658		-0.13764	4.112692	-2.77882
H	1.078325	157.3204	-3.97925	0.371582	4.93366	-3.25786
C	1.36308	72.0166	178.1049	-1.31538	3.498459	-3.08483
H	1.078678	130.6515	178.7825	-2.02132	3.687835	-3.87813
N	1.341642	36.40045	179.9419	-1.52393	2.515837	-2.13409
N	1.339824	108.6445	0.00095	0.359216	3.495333	-1.64541
C	1.480186	125.5846	-179.902	1.624387	3.837376	-0.95744
H	1.093914	107.2177	-123.455	1.574117	4.898354	-0.69582
H	1.092692	106.3957	-8.33264	1.634309	3.264282	-0.02715
C	1.530859	112.9111	113.7122	2.863689	3.533607	-1.80324
H	1.096922	109.6816	58.68567	2.814356	4.093448	-2.74525
H	1.097214	109.1988	-57.9876	2.867828	2.468774	-2.0678
C	1.536183	111.5395	-179.907	4.1574	3.897862	-1.05928
H	1.097365	109.2989	-57.9025	4.133867	4.961477	-0.79024
H	1.098314	109.2746	58.02687	4.200421	3.341691	-0.11317
C	1.531377	112.5461	-179.868	5.412567	3.606004	-1.88662
H	1.094679	111.3014	60.26153	5.415012	4.177175	-2.82047
H	1.092913	110.803	179.9425	6.314629	3.87611	-1.33183
H	1.095277	111.4089	-60.1138	5.484116	2.54364	-2.14332
C	1.480135	125.6813	-179.326	-2.69118	1.6082	-2.06684
H	1.093892	107.6874	34.8591	-2.9198	1.438496	-1.01065
H	1.091668	106.2337	151.2377	-3.52341	2.146925	-2.5239
C	1.534274	113.6443	-87.1262	-2.46834	0.265658	-2.7753

H	1.096304	105.769	-171.765	-3.44363	-0.23501	-2.77851
H	1.096787	110.3201	-56.7578	-2.19502	0.42956	-3.82476
C	1.537773	113.3137	68.34286	-1.43545	-0.62996	-2.0712
H	1.096046	108.4414	50.61401	-1.70187	-0.69844	-1.01023
H	1.096962	109.9244	-67.391	-0.43815	-0.17894	-2.14406
C	1.525751	110.7916	171.832	-1.40482	-2.01945	-2.70071
H	1.094283	110.5701	-61.1511	-2.3794	-2.50741	-2.60297
H	1.094347	111.2102	58.71243	-1.16053	-1.96613	-3.76611
C	2.535979	88.64411	-179.84	0.391163	-2.66974	-1.03255
C	2.211534	63.95907	-177.738	-0.16798	-4.21754	-2.5099
H	1.081811	98.83443	2.626265	0.434947	-1.74806	-0.46782
H	1.078762	157.2012	-0.40808	-0.69935	-4.65232	-3.34196
C	1.361786	72.07789	-179.647	0.793569	-4.73985	-1.69929
H	1.078504	130.6295	179.7377	1.256217	-5.71407	-1.69602
N	1.342017	27.54063	-177.165	-0.40708	-2.92356	-2.08106
N	1.340095	136.1226	-178.282	1.129104	-3.76031	-0.78366
C	1.48053	125.8701	178.8498	2.162436	-3.88687	0.269037
H	1.093706	107.0487	128.9013	1.935936	-4.79615	0.833035
H	1.092916	106.5704	13.9504	2.026827	-3.037	0.942689
C	1.531445	112.775	-108.412	3.584622	-3.92483	-0.29777
H	1.097018	109.4744	-57.7088	3.67473	-4.7595	-1.00393
H	1.09709	109.2527	58.82635	3.771111	-3.00678	-0.86874
C	1.536028	111.6264	-179.095	4.633214	-4.07883	0.814041
H	1.097397	109.3024	57.62636	4.425148	-4.99271	1.38485
H	1.098477	109.3452	-58.3435	4.537687	-3.24471	1.522394
C	1.531572	112.4903	179.6014	6.063205	-4.12886	0.267839
H	1.092875	110.7622	179.7645	6.785211	-4.2344	1.081438
H	1.094632	111.2918	-60.5657	6.200642	-4.978	-0.40913
H	1.095073	111.4091	59.84343	6.312653	-3.21619	-0.28353
C	5.553647	112.0395	-6.73362	2.226169	1.047466	3.806724
C	1.532766	114.6326	-81.9216	1.480948	1.654154	5.000854
C	1.53348	114.8721	36.27103	0.121783	2.278807	4.663156
C	1.549482	84.84761	32.57087	1.455848	-0.08337	3.079596
C	1.522627	114.8197	59.9691	-0.89171	1.31619	4.059347
H	1.097547	26.20271	-168.674	2.492463	1.827384	3.081862
H	1.098088	108.8574	158.6402	1.335166	0.875348	5.761121
H	1.097339	111.2103	-63.7366	0.235529	3.135017	3.986311
H	1.094937	112.3741	43.88918	-0.9138	0.353392	4.580326
H	1.097497	109.6067	-158.856	1.011745	-0.76131	3.819639
H	1.094217	124.3388	139.9871	3.171371	0.622674	4.158086
H	1.094634	108.8193	-86.1815	2.116939	2.414934	5.464484

H	1.096117	108.9381	179.6462	-0.32597	2.67462	5.582024
H	1.091649	109.9753	82.89804	2.143306	-0.66718	2.464567
H	1.090378	111.0028	165.3138	-1.8958	1.740686	4.082469
C	1.51198	113.9007	-35.5784	0.383454	0.41395	2.136872
O	1.345409	119.8032	-65.1741	-0.68461	1.070134	2.625523
O	1.226096	123.0399	113.4435	0.444027	0.271388	0.9206
C	3.758263	117.1251	-128.681	-4.07762	-0.15035	1.566054
H	1.099613	88.07994	-171.528	-4.50764	0.738491	2.049995
H	1.100004	77.44998	80.02056	-3.80514	-0.86114	2.360113
C	1.521015	156.2687	-31.0089	-5.10245	-0.78183	0.636304
H	1.098553	108.8155	-74.6744	-5.34887	-0.06483	-0.15868
H	1.098773	108.7168	41.36221	-4.64573	-1.65402	0.148456
C	1.53498	112.9099	163.4148	-6.38637	-1.20658	1.362466
H	1.097884	109.3076	-57.7186	-6.13318	-1.9117	2.165003
H	1.097577	109.2758	58.07464	-6.83131	-0.33159	1.853468
C	1.531237	112.8968	-179.84	-7.4163	-1.84582	0.426889
H	1.095705	111.3211	60.1894	-7.01526	-2.74642	-0.05129
H	1.09323	111.0704	-179.958	-8.31741	-2.1358	0.973746
H	1.095446	111.3319	-60.1073	-7.71906	-1.15274	-0.36556
O	1.438742	48.19895	-51.9675	-2.90949	0.212701	0.808659
H	0.970004	108.3717	-6.02817	-2.24855	0.573419	1.420174

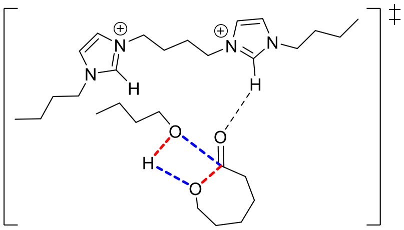

TS3-20 (29.29)

Atom	Bond	Angle	Dihedral	X	Y	Z
C				-2.15815	-2.11098	-0.97993
H	1.090047			-1.56299	-1.95551	-0.08003
C	2.213151	162.5236		-3.8675	-2.33988	-2.36693
H	1.078521	157.1726	1.602194	-4.90072	-2.36995	-2.67476
C	1.362355	72.17827	-176.372	-2.72456	-2.56702	-3.0727
H	1.078569	130.5945	178.505	-2.58031	-2.83764	-4.10675
N	1.3428	72.14468	0.066206	-1.66711	-2.41845	-2.19131
N	1.339361	108.4469	-0.03778	-3.49381	-2.05887	-1.06479
C	1.481102	125.7572	-179.741	-4.42014	-1.7454	0.047558
H	1.093679	107.0289	-128.067	-5.16566	-2.54501	0.078509

H	1.092642	106.6202	-13.1081	-3.83422	-1.80347	0.967989
C	1.531041	112.8356	109.2287	-5.08356	-0.37351	-0.10038
H	1.097234	109.569	58.08709	-5.63192	-0.33093	-1.0498
H	1.097438	109.1506	-58.5214	-4.30576	0.399188	-0.14868
C	1.536201	111.5924	179.573	-6.04522	-0.08235	1.061666
H	1.097292	109.2895	-57.9135	-6.80899	-0.86895	1.105889
H	1.097927	109.3304	58.0391	-5.49498	-0.13379	2.010365
C	1.531406	112.5062	-179.852	-6.7237	1.284485	0.932643
H	1.094816	111.328	60.35746	-7.31401	1.350788	0.01299
H	1.092854	110.7769	-179.932	-7.39934	1.462767	1.772912
H	1.095347	111.3741	-60.08	-5.98785	2.095735	0.91898
C	1.477961	125.5925	-178.177	-0.23827	-2.61012	-2.51699
H	1.092455	107.0498	12.62473	0.299602	-2.68147	-1.5688
H	1.092905	106.9834	128.3667	-0.15386	-3.57173	-3.02945
C	1.533702	113.8181	-109.364	0.35611	-1.48973	-3.37935
H	1.096893	105.6235	-174.794	1.377838	-1.8103	-3.617
H	1.096427	110.3583	-60.4754	-0.17341	-1.42391	-4.33718
C	1.537089	113.414	64.78137	0.386787	-0.12542	-2.67199
H	1.094251	108.8631	43.05569	0.724302	-0.26896	-1.64103
H	1.096294	110.5542	-74.4918	-0.61938	0.307552	-2.62705
C	1.530488	111.1296	164.025	1.323176	0.84816	-3.39151
H	1.094825	110.7623	-57.8418	2.337514	0.440174	-3.44903
H	1.092792	111.252	61.836	0.985427	1.040939	-4.41276
C	2.523049	93.2346	-162.471	1.471255	2.397265	-1.40553
C	2.213	64.45688	-145.954	1.539786	3.378201	-3.38806
H	1.082915	97.96074	33.37423	1.40064	1.648906	-0.62599
H	1.078577	157.316	0.826484	1.531416	3.448695	-4.4643
C	1.362487	72.02085	-177.784	1.68008	4.34306	-2.43636
H	1.078433	130.64	179.9196	1.807532	5.409743	-2.53102
N	1.343982	28.12879	-142.807	1.406494	2.168971	-2.72839
N	1.338664	136.5458	-149.094	1.635872	3.710999	-1.20798
C	1.48185	125.6713	-178.367	1.722637	4.382426	0.110182
H	1.093635	106.9332	128.656	2.62344	5.002348	0.093204
H	1.09276	106.5939	13.70737	1.875905	3.594882	0.852076
C	1.531053	112.7904	-108.67	0.479852	5.215876	0.434173
H	1.097134	109.5863	-57.7881	0.326793	5.968018	-0.34977
H	1.097239	109.2025	58.83705	-0.40351	4.565094	0.425234
C	1.536295	111.5444	-179.217	0.608194	5.913971	1.796668
H	1.097354	109.2746	57.92361	1.501486	6.551317	1.794407
H	1.098024	109.332	-58.0248	0.77015	5.159509	2.57783
C	1.531403	112.4917	179.8496	-0.62277	6.757367	2.141059

H	1.092873	110.7434	-179.856	-0.50028	7.242174	3.112825
H	1.094775	111.3148	-60.1772	-0.78742	7.543427	1.397061
H	1.095201	111.4161	60.27267	-1.52833	6.143091	2.186876
C	5.645811	116.8722	30.3398	0.983221	0.04826	3.705167
C	1.535094	136.8505	-64.6951	-0.1026	-0.69227	4.498341
C	1.53661	116.9073	23.14596	-1.21134	-1.3787	3.68551
C	1.546204	69.22907	39.50541	1.819055	-0.85147	2.765695
C	1.52911	115.6549	64.57109	-0.75786	-2.53119	2.788682
H	1.096196	40.71739	-128.27	0.547666	0.868427	3.122703
H	1.098324	108.6893	146.4887	0.379585	-1.44364	5.138074
H	1.098422	110.6683	-59.202	-1.74749	-0.64703	3.066047
H	1.099141	110.5768	41.56965	-0.02093	-3.15889	3.309294
H	1.091108	110.5543	-168.633	1.97102	-1.83522	3.212542
H	1.094531	111.2238	140.8681	1.673319	0.507542	4.419885
H	1.095546	107.9484	-99.2678	-0.56941	0.024984	5.182342
H	1.096497	107.7879	-175.194	-1.94499	-1.78202	4.393598
H	1.096467	110.211	73.09144	2.810921	-0.41276	2.604539
H	1.097432	109.9341	159.0817	-1.61141	-3.17894	2.551549
C	1.503276	112.0634	-43.4968	1.211019	-0.95529	1.3948
O	1.439143	112.823	-82.8565	-0.23719	-2.08293	1.524126
O	1.199687	128.0434	59.18413	0.97819	-0.06922	0.620255
C	2.822508	87.45885	-166.576	2.965874	-3.11374	0.917152
H	1.095365	133.1207	130.0409	2.817195	-4.12865	0.532883
H	1.095861	86.66762	19.07578	3.070944	-3.18588	2.005576
C	1.521409	104.2806	-92.0049	4.191105	-2.46882	0.28662
H	1.097436	108.7257	-87.0032	4.034843	-2.40074	-0.7975
H	1.098523	109.4463	29.16154	4.293156	-1.43917	0.655616
C	1.536262	112.2202	151.0828	5.47825	-3.25305	0.583821
H	1.097671	109.3726	-58.168	5.618248	-3.32485	1.670157
H	1.097552	109.273	57.66457	5.366854	-4.28297	0.221198
C	1.531283	112.7764	179.7278	6.718991	-2.6184	-0.05069
H	1.095476	111.3411	60.06975	6.878566	-1.59941	0.318446
H	1.093004	110.9367	179.8815	7.616091	-3.19822	0.181009
H	1.095406	111.3253	-60.3283	6.6283	-2.5703	-1.14128
O	1.45221	30.43833	160.6673	1.758339	-2.38272	0.57599
H	1.153641	120.8845	-94.5881	0.735145	-2.69499	1.007793

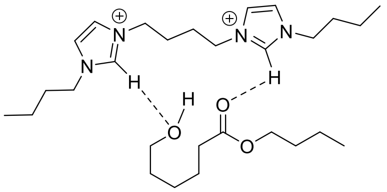

P3-21 (-15.33)

Atom	Bond	Angle	Dihedral	X	Y	Z
C				4.40397	0.345355	-0.66591
H	1.087625			4.039691	1.358788	-0.51365
C	2.209472	163.4871		5.542337	-1.54202	-0.51193
H	1.078604	157.1879	8.887049	6.243834	-2.24905	-0.09793
C	1.363226	72.19039	-170.169	4.755808	-1.61893	-1.62272
H	1.077684	130.2558	178.4134	4.663275	-2.40167	-2.35767
N	1.342487	72.31187	0.362268	4.0448	-0.43307	-1.69902
N	1.338187	108.7004	-0.355	5.312426	-0.30794	0.068027
C	1.482165	125.728	-178.946	5.947127	0.195986	1.309006
H	1.093421	106.8489	-132.958	7.021089	0.015145	1.211682
H	1.092696	106.6645	-18.1545	5.795035	1.277971	1.321683
C	1.531029	112.8667	104.3384	5.384308	-0.45883	2.573326
H	1.097352	109.5012	58.03271	5.518661	-1.54628	2.513525
H	1.097379	109.176	-58.6139	4.303743	-0.27439	2.624328
C	1.536337	111.5436	179.5668	6.070057	0.077147	3.839348
H	1.097346	109.3028	-58.0196	7.150365	-0.10277	3.77055
H	1.097656	109.3136	57.96722	5.941324	1.166114	3.88861
C	1.531382	112.4908	-179.983	5.523302	-0.56427	5.117933
H	1.094956	111.3456	60.37142	5.673538	-1.64886	5.115438
H	1.092835	110.7226	-179.932	6.029292	-0.16217	5.999171
H	1.095199	111.3768	-60.1493	4.451216	-0.37272	5.233702
C	1.481951	124.0187	-174.124	3.150009	0.00563	-2.79587
H	1.092956	106.1815	-15.2551	2.661353	0.917727	-2.44395
H	1.094403	107.1724	99.87923	3.786606	0.27131	-3.6455
C	1.530957	114.4313	-139.004	2.132091	-1.04724	-3.24211
H	1.096733	105.3479	-173.856	1.633357	-0.61576	-4.11841
H	1.097262	109.7058	-60.8739	2.657677	-1.93557	-3.61443
C	1.537974	116.1519	64.75293	1.077605	-1.4553	-2.19956
H	1.096198	110.1229	53.38429	0.566841	-0.56623	-1.81184
H	1.096212	110.7666	-65.1305	1.548382	-1.95829	-1.34689
C	1.53507	109.3064	174.3678	0.060983	-2.40368	-2.85031
H	1.09455	110.6492	-60.9876	-0.45323	-1.90809	-3.67977
H	1.092642	110.576	57.77747	0.563535	-3.28381	-3.25858
C	2.510375	98.16886	-161.661	-1.78171	-2.16396	-1.16242

C	2.216591	64.73673	-133.842	-1.32239	-4.24488	-1.77238
H	1.0908	97.84118	44.49019	-1.74324	-1.07933	-1.05314
H	1.078391	157.2891	0.071475	-0.81869	-5.03832	-2.30122
C	1.362091	72.01203	-177.649	-2.36916	-4.28309	-0.90168
H	1.078425	130.6938	179.6157	-2.93891	-5.11686	-0.52325
N	1.343689	28.48057	-130.531	-0.96503	-2.91452	-1.92082
N	1.337559	136.6776	-137.283	-2.64025	-2.97686	-0.53699
C	1.480694	125.1184	-177.84	-3.68826	-2.54026	0.413537
H	1.093644	106.8071	134.2281	-4.60589	-3.06666	0.13619
H	1.092558	106.4067	19.25326	-3.84587	-1.47465	0.231026
C	1.531188	112.7144	-103.315	-3.31283	-2.81135	1.873022
H	1.097296	109.5552	-57.2157	-3.10767	-3.88095	2.006907
H	1.097262	109.1208	59.39142	-2.38369	-2.278	2.110151
C	1.535846	111.6129	-178.78	-4.429	-2.37681	2.834352
H	1.097501	109.2993	58.2066	-5.354	-2.91042	2.581063
H	1.098064	109.2964	-57.7542	-4.63902	-1.30906	2.68758
C	1.53144	112.5347	-179.848	-4.07367	-2.63526	4.301408
H	1.092984	110.8332	-179.922	-4.88615	-2.31769	4.95992
H	1.094857	111.2834	-60.2061	-3.89486	-3.69963	4.485442
H	1.095213	111.3702	60.17348	-3.17227	-2.08748	4.596188
C	5.850534	99.79391	30.73903	-0.83514	3.343804	0.568864
C	1.534172	93.44157	1.853849	0.33224	3.547301	-0.40556
C	1.53421	111.8364	-122.141	1.62435	3.93534	0.32497
C	1.535473	61.63199	118.3311	-2.17848	3.00836	-0.0949
C	1.520049	114.7016	-174.562	2.790751	4.279626	-0.58691
H	1.095795	63.54515	-108.65	-0.58615	2.556149	1.288846
H	1.094442	109.841	0.359726	0.491017	2.636018	-0.99051
H	1.09861	110.1933	61.77732	1.927631	3.132844	1.011239
H	1.097442	110.1146	53.27695	2.490621	5.049306	-1.30933
H	1.098604	110.3741	-124.697	-2.35285	3.664692	-0.95847
H	1.096152	157.7279	-176.566	-0.97623	4.263309	1.148649
H	1.098988	109.1892	117.201	0.073016	4.342164	-1.11885
H	1.097595	109.4948	-54.4445	1.439739	4.816627	0.952638
H	1.095506	109.9788	119.2842	-3.00061	3.213152	0.599583
H	1.097594	110.1575	172.1547	3.626756	4.677898	0.002316
C	1.516753	114.064	-2.40632	-2.29769	1.568544	-0.5567
O	1.448693	109.9038	-67.1907	3.224829	3.096056	-1.30067
O	1.225131	123.0778	-8.6095	-1.35547	0.785496	-0.55464
C	2.472655	90.26177	171.4477	-4.69402	1.915748	-1.05775
H	1.092371	124.9702	138.2435	-5.21015	1.535747	-1.94233
H	1.092012	86.28639	29.03043	-4.43048	2.958531	-1.24654

C	1.522929	111.797	-83.1549	-5.55965	1.763335	0.185938
H	1.097448	108.8811	-84.3816	-5.78357	0.699304	0.334571
H	1.097996	109.8985	32.13518	-5.00244	2.094958	1.07202
C	1.536828	111.8448	154.142	-6.86948	2.559087	0.07203
H	1.097674	109.3315	-58.4687	-6.6374	3.620167	-0.08651
H	1.097486	109.3102	57.42415	-7.41847	2.228789	-0.81903
C	1.531461	112.6678	179.4929	-7.76064	2.409061	1.308435
H	1.095267	111.3589	60.08997	-7.25299	2.764412	2.211554
H	1.093029	110.8685	179.9202	-8.6817	2.986932	1.196966
H	1.095256	111.3237	-60.3003	-8.04287	1.363424	1.471457
O	1.343325	119.7545	170.7101	-3.48334	1.100301	-0.98038
H	0.967144	106.7441	168.5528	3.832996	3.394349	-1.99097
